# Direct conversion of amino acids to oxetanol bioisosteres *via* photoredox catalysis†

**DOI:** 10.1039/d3sc00936j

**Published:** 2023-09-15

**Authors:** Avelyn Mae V. Delos Reyes, Christopher S. Nieves Escobar, Alberto Muñoz, Maya I. Huffman, Derek S. Tan

**Affiliations:** a Pharmacology Graduate Program, Weill Cornell Graduate School of Medical Sciences, Memorial Sloan Kettering Cancer Center New York New York 10065 USA tand@mskcc.org; b Tri-Institutional PhD Program in Chemical Biology, Memorial Sloan Kettering Cancer Center New York New York 10065 USA; c Chemical Biology Program, Sloan Kettering Institute, Memorial Sloan Kettering Cancer Center New York New York 10065 USA; d Tri-Institutional Chemical Biology Summer Program, Memorial Sloan Kettering Cancer Center New York New York 10065 USA; e Tri-Institutional Research Program, Memorial Sloan Kettering Cancer Center New York New York 10065 USA

## Abstract

Carboxylic acids are an important structural feature in many drugs, but are associated with a number of unfavorable pharmacological properties. To address this problem, carboxylic acids can be replaced with bioisosteric mimics that interact similarly with biological targets but avoid these liabilities. Recently, 3-oxetanols have been identified as useful carboxylic acid bioisosteres that maintain similar hydrogen-bonding capacity while decreasing acidity and increasing lipophilicity. However, the installation of 3-oxetanols generally requires multistep *de novo* synthesis, presenting an obstacle to investigation of these promising bioisosteres. Herein, we report a new synthetic approach involving direct conversion of carboxylic acids to 3-oxetanols using a photoredox-catalyzed decarboxylative addition to 3-oxetanone. Two versions of the transformation have been developed, in the presence or absence of CrCl_3_ and TMSCl cocatalysts. The reactions are effective for a variety of *N*-aryl α-amino acids and have excellent functional group tolerance. The Cr-free conditions generally provide higher yields and avoid the use of chromium reagents. Further, the Cr-free conditions were extended to a series of *N*,*N*-dialkyl α-amino acid substrates. Mechanistic studies suggest that the Cr-mediated reaction proceeds predominantly *via in situ* formation of an alkyl-Cr intermediate while the Cr-free reaction proceeds largely *via* radical addition to a Brønsted acid-activated ketone. Chain propagation processes provide quantum yields of 5 and 10, respectively.

## Abstract 2

Carboxylic acids can be converted directly to bioisosteric 3-oxetanols by leveraging the unique reactivity of photoredox catalysis. Cr-mediated and Cr-free variants of the reaction have been developed, both having quantum yields greater than 5.

## Graphical abstract

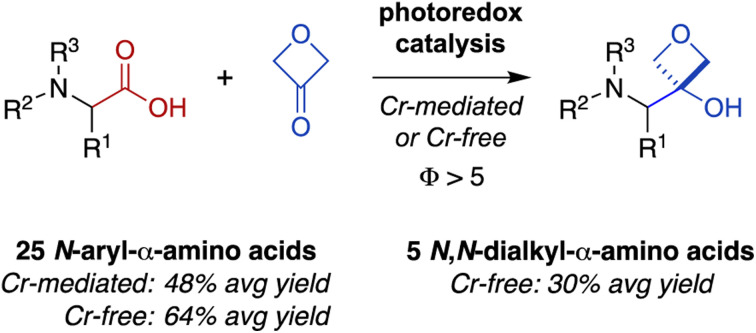

## Introduction

**Fig. 1:**
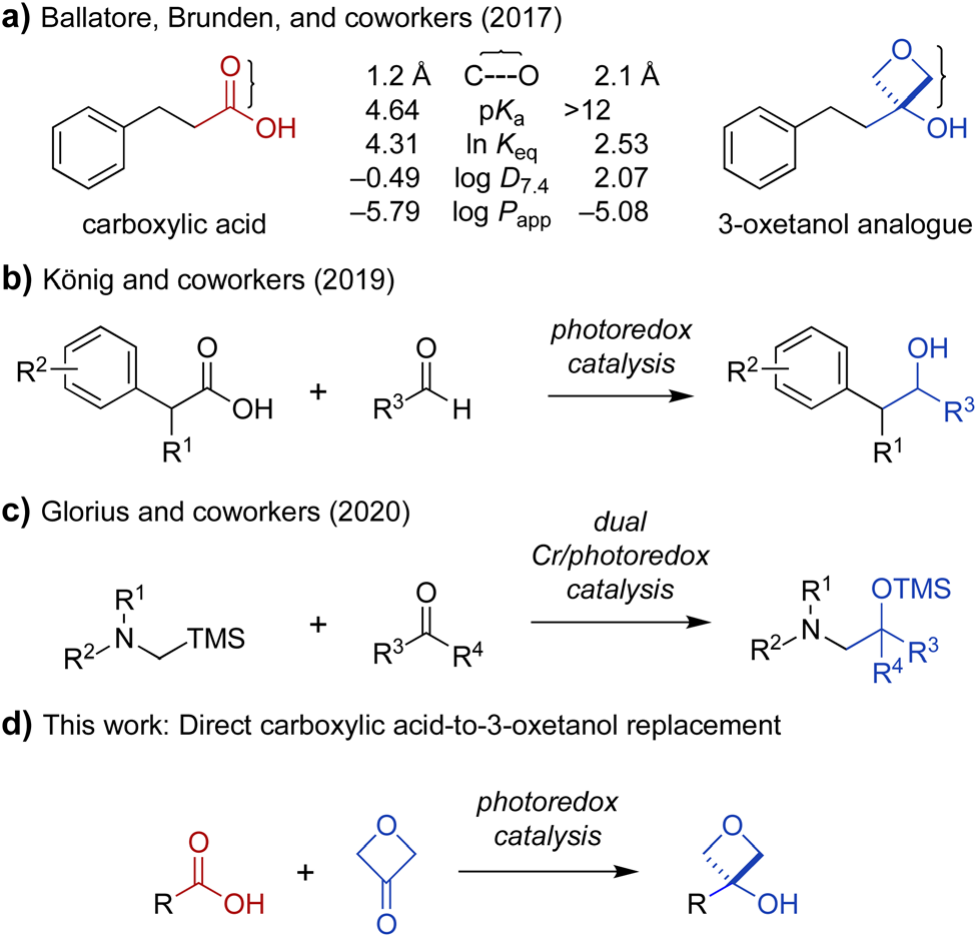
(a) Physicochemical and pharmacological characteristics of carboxylic acids and 3-oxetanol bioisosteres (C⋯O = distance from carbonyl carbon to oxygen; ln *K*_eq_ = hydrogen-bonding equilibrium constant determined by colorimetric assay for blue-shift of a fluorescent pyrazinone; log *D*_7.4_ = 1-octanol/water distribution coefficient, pH 7.4; log *P*_app_ = apparent permeability coefficient in parallel artificial membrane permeability assay).^10,13^ (b) Decarboxylative coupling of carboxylic acids and aldehydes under photoredox catalysis.^14^ (c) Addition of α-silyl amine-derived nucleophiles to aldehydes and ketones under photoredox catalysis.^15^ (d) Proposed direct transformation of carboxylic acids to 3-oxetanol bioisosteres.

The carboxylic acid moiety is an important structural feature found in many drugs and other bioactive compounds.^1^ However, it is also associated with several pharmacological liabilities, including limited permeability across biological membranes, high plasma protein binding, rapid renal clearance, and conversion to chemically reactive metabolites associated with toxicity.^2–7^ Indeed, small-molecule drugs containing carboxylic acid moieties have been withdrawn from the market at a much higher rate (39%)^8^ than their prevalence would predict (13%).^1^ One approach to circumvent undesired pharmacological properties associated with a given chemical group is to replace it with a bioisostere, a structural mimic that can induce a similar biological response.^9^ Several carboxylic acid bioisosteres have been reported, including hydroxamic acids, phosphonic acids, tetrazoles, and isothiazoles.^5^ Recently, 3-oxetanols have also been identified as promising carboxylic acid bioisosteres that can accommodate similar hydrogen-bonding interactions with biological targets while being less acidic, non-anionic under physiologic conditions, and more lipophilic to provide increased membrane permeability (Fig. 1a).^10^ While several synthetic approaches to 3-oxetanols have been reported,^10–13^ they require multistep *de novo* synthesis, presenting an obstacle to broad exploration of this promising class of bioisosteres. In contrast, a method for direct conversion of carboxylic acids to 3-oxetanols would provide expedient access to this motif, facilitating its investigation in medicinal chemistry campaigns. Herein, we report a new synthetic approach that enables direct conversion of α-amino acids to corresponding 3-oxetanols using visible light photoredox-catalyzed decarboxylative addition to 3-oxetanone. The reaction can be carried out in the presence or absence of CrCl_3_ and TMSCl cocatalysts, with the Cr-free conditions generally providing higher yields and avoiding the use of chromium reagents. The reactions provide broad substrate scope and functional group compatibility across *N*-aryl α-amino acid substrates. In addition, the Cr-free reaction was extended to a series of *N*,*N*-dialkyl α-amino acid substrates. Mechanistic studies suggest that the Cr-mediated reaction proceeds primarily *via* a Nozaki–Hiyama–Kishi reaction manifold, while the Cr-free reaction proceeds largely *via* 1,2-radical addition. The reactions have quantum yields of 5 and 10, respectively, indicative of chain propagation mechanisms.

Oxetanes have been investigated widely as bioisosteric replacements for *gem*-dimethyl groups,^13,16–19^ and have also attracted attention as carbonyl bioisosteres.^13,17–23^ Recently, the use of 3-oxetanols as carboxylic acid bioisosteres has been explored by Ballatore, Brunden, and coworkers.^10^ Comparison of the physicochemical properties of hydrocinnamic acid and its 3-oxetanol analogue, indicate that the latter is more lipophilic and membrane permeable (log *D*_7.4_: −0.49 *vs.* 2.07, log *P*_app_: −5.79 *vs.* −5.08) (Fig. 1a).^10^ A 3-oxetanol analogue of ibuprofen was also evaluated and shown to have inhibitory activity against the cyclooxygenase (COX) pathway in a cell-based assay.^10^ This work provided important proof of concept for the use of 3-oxetanols as effective carboxylic acid bioisosteres. However, access to 3-oxetanols generally requires multistep *de novo* synthesis. Examples include addition of organometallic reagents to 3-oxetanone,^10,16^ Paternò–Büchi reaction of silyl enol ethers and aldehydes,^11^ and ring contraction of pentofuranose sugars.^12^ This lack of direct synthetic access from carboxylic acid substrates presents an obstacle to the broad exploration of 3-oxetanols as carboxylic acid bioisosteres. To address this problem, we sought to develop a method for direct conversion of carboxylic acids to the corresponding 3-oxetanol analogues.

Photoredox catalysis has emerged as an indispensable tool in synthetic organic chemistry. This mode of catalysis relies on photosensitive catalysts that convert light into chemical energy through single-electron transfer (SET) events with organic substrates, generating reactive radical intermediates under mild conditions, which can then engage in a variety of chemical transformations.^24,25^ With this in mind, we noted recent work by König and coworkers demonstrating photocatalytic decarboxylative activation of phenylacetic acids using the organic dye 4CzIPN (1,2,3,5-tetrakis(carbazole-9-yl)-4,6-dicyanobenzene, 2,4,5,6-tetrakis(9*H*-carbazol-9-yl) isophthalonitrile) for benzylation of aldehydes (Fig. 1b).^14^ More recently, Glorius and coworkers reported a dual Cr/photoredox catalytic system to convert trimethylsilylmethylamines to α-amino carbanion equivalents for addition to aldehydes and ketones (Fig. 1c).^15^ Inspired by these reports, we envisioned that carboxylic acids could be activated under photoredox catalysis for Nozaki–Hiyama–Kishi-type addition^26^ to 3-oxetanone to form the corresponding 3-oxetanol analogues (Fig. 1d), facilitating access to these understudied bioisosteres.

## Results and discussion

### Development of photoredox-catalyzed reaction for direct conversion of *N*-aryl α-amino acids to 3-oxetanol analogues

Photon-induced oxidative decarboxylation of α-amino acids is well known^27,28^ and the synthetic utility of the resulting α-amino radicals has been demonstrated.^29–32^ Rueping and coworkers have reported Ir photoredox-mediated decarboxylative couplings of *N*-aryl amino acids with enones^33^ and Zeng, Zhong, and coworkers,^34^ and Peng and coworkers^35^ have separately reported related couplings with aldehydes and ketones. With this in mind, we selected *N*-phenyl glycine (1a) as an initial substrate because it is readily oxidized (*E*_1/2_ = +0.42 V *versus* standard calomel electrode [SCE] in CH_3_CN)^33,36^ and commercially available. Unfortunately, treatment of *N*-phenyl glycine (1a) and 3-oxetanone (2) under conditions similar to those reported by Glorius for dual Cr/photoredox catalysis with 4CzIPN, did not afford any of the 3-oxetanol product 3a (Table 1, entry 1).^15^ However, addition of CsOAc, a base commonly used in decarboxylative photoredox platforms,^37^ resulted in a 7% yield of the desired product (entry 2). Previous studies have used TMSCl as an oxophilic additive to facilitate release of Cr back into the catalytic cycle,^28,38^ and inclusion of TMSCl resulted in an increased yield of 22% (entry 3). Carrying out the reaction in DMF instead of DMA slightly increased the yield to 25% (entry 4).

**Table 1:** Discovery and optimization of the carboxylic acid-to-3-oxetanol transformation

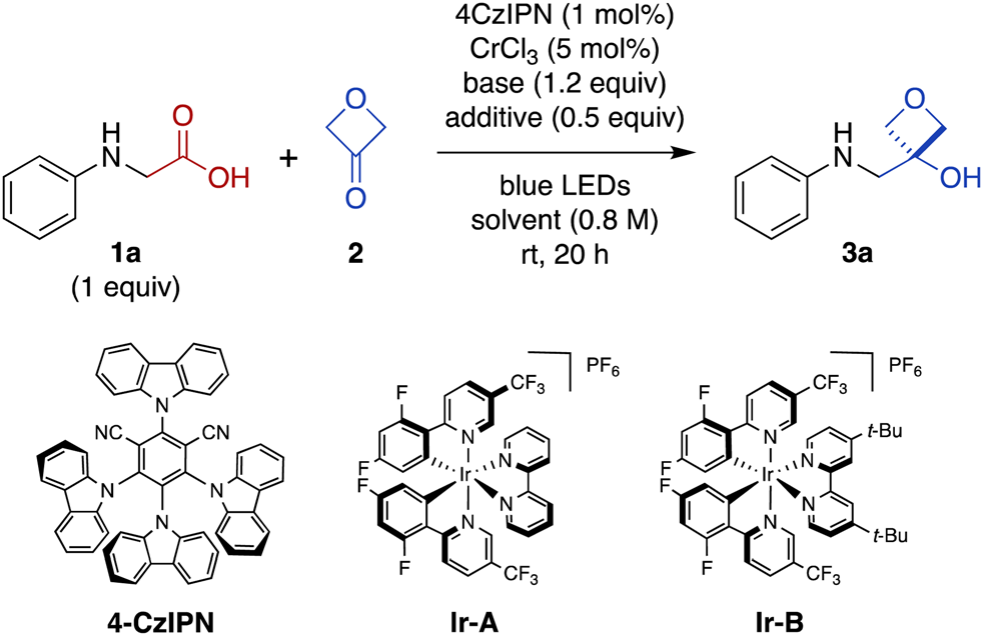					
Entry	2 (equiv.)	Base	Additive	Solvent	Yield^a^ (%)
1	0.5	—	—	DMA	0
2	0.5	CsOAc	—	DMA	7
3	0.5	CsOAc	TMSCl	DMA	22
4	0.5	CsOAc	TMSCl	DMF	25
5^b^	0.5	CsOAc	TMSCl	DMF	24
6^c^	0.5	CsOAc	TMSCl	DMF	17
7	0.5	KHCO_3_	TMSCl	DMF	24
8	0.5	CsOPiv	TMSCl	DMF	32
9	0.5	CsOPiv	TMSCl	THF	38
10	0.5	CsOPiv	TMSCl	CH_3_CN	40
11	0.5	CsOPiv	TESCl	CH_3_CN	37
12	1.0	CsOPiv	TMSCl	CH_3_CN	60
13	2.0	CsOPiv	TMSCl	CH_3_CN	55
14^d^	1.0	CsOPiv	TMSCl	CH_3_CN	50
15^d^	1.0	CsOPiv	—	CH_3_CN	47
16^e^	1.0	CsOPiv	TMSCl	CH_3_CN	0

a Yields based on ^1^H-NMR analysis of crude reaction product in the presence of an internal standard, relative to *N*-phenyl glycine (theoretical maximum 50% for entries 1–11).

b Photocatalyst: Ir-A = [Ir{dF(CF_3_)_2_ppy}_2_(bpy)]PF_6_ = [2,2′-bipyridine-*N*1,*N*1′]bis[3,5-difluoro-2-[5-(trifluoromethyl)-2-pyridinyl-*N*]phenyl-*C*]iridium(iii) hexafluorophosphate.

c Photocatalyst: Ir-B = [Ir{dF(CF_3_)ppy}_2_(dtbpy)]PF_6_ = [4,4′-bis(1,1-dimethylethyl)-2,2′-bipyridine-*N*1,*N*1′]bis[3,5-difluoro-2-[5-(trifluoromethyl)-2-pyridinyl-*N*]phenyl-*C*]iridium(iii) hexafluorophosphate.

d In absence of CrCl_3_.

e In absence of blue LED light. 4CzIPN = 1,2,3,5-tetrakis(carbazole-9-yl)-4,6-dicyanobenzene, 2,4,5,6-tetrakis(9*H*-carbazol-9-yl) isophthalonitrile; DMA = *N*,*N*-dimethyl acetamide, DMF = *N*,*N*-dimethyl formamide; TES = triethylsilyl; THF = tetrahydrofuran; TMS = trimethylsilyl.

We also evaluated alternative photocatalysts Ir-A and Ir-B,^24^ but these reactions provided somewhat lower yields (Table 1, entries 5 and 6). We then tested other bases (entries 7 and 8), solvents (entries 8–10), and silyl chlorides (entries 10 and 11) (see ESI Table S1† for complete details).

In these initial experiments, we used 3-oxetanone as the limiting substrate, by analogy to the conditions reported by Glorius.^15^ Next, we investigated alternative stoichiometric ratios (entries 11–13 and ESI Table S1†), and found that the reaction was most effective with equimolar amounts of the two substrates, providing a serviceable 60% yield (entry 12). Interestingly, the reaction also proceeded in the absence of CrCl_3_ (Table 1, entry 14), as well as in the absence of both CrCl_3_ and TMSCl (entry 15), albeit in lower yields; the mechanistic implications of this finding are discussed below. In contrast, control reactions performed in the absence of light (entry 15) or photocatalyst (see ESI Table S1†) did not afford any of the desired product.

### Substrate scope and functional group tolerance of the Cr-mediated carboxylic acid-to-3-oxetanol transformation

**Fig. 2:**
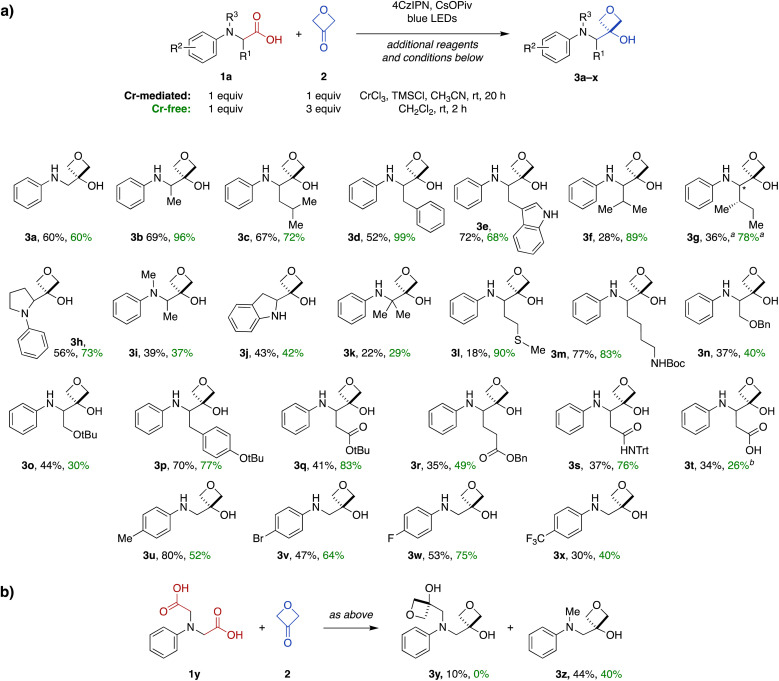
(a) Scope of the carboxylic acid-to-3-oxetanol transformation for *N*-aryl α-amino acid substrates under Cr-mediated (black yields) and Cr-free reaction conditions (green yields). (b) Conversion of diacid 1y to mono- (3y) and di-oxetanol (3z) products. Cr-mediated reaction conditions: 1 mol% 4CzIPN, 5 mol% CrCl_3_, 0.5 equiv. TMSCl, 1.2 equiv. CsOPiv, 0.8 M in CH_3_CN based on amino acid substrate 1, blue LED light, rt, 20 h. Cr-free reaction conditions: 2 mol% 4CzIPN, 1.2 equiv. CsOPiv, 0.5 M in CH_2_Cl_2_ based on amino acid substrate 1, blue LED light, rt, 2 h. ^*a*^diastereomeric ratio = 1 : 1.4. ^*b*^Reaction carried out in isopropanol instead of CH_2_Cl_2_.

Next, we investigated the scope of the Cr-mediated reaction using other *N*-aryl α-amino acid substrates (Fig. 2). A variety of other substrates were tolerated, including systems derived from alanine (3b), leucine (3c), phenylalanine (3d), tryptophan (3e), valine (3f) and isoleucine (3g). The reaction was also effective in a proline-derived system containing a tertiary amine (3h), as well as a corresponding acyclic *N*-methyl alanine-derived system (3i) and an indoline-derived system (3j). Notably, the reaction also proceeded efficiently with an α,α-dimethylglycine-derived substrate to form 3-oxetanol 3k having two adjacent quaternary carbons.

A wide range of functional groups were tolerated in other substrates, including a methionine thioether (3l), Boc-protected lysine side chain (3m), serine benzyl and *t*-butyl ethers (3n and 3o), a tyrosine aryl ether (3p), protected aspartate and glutamate esters (3q and 3r), and an asparagine *N*-trityl amide (3s). Notably, a free carboxylic acid was also tolerated in an aspartate-derive system (3t), with transformation to the 3-oxetanol occurring regiospecifically at the main-chain carboxylate (34%).

We also investigated the influence of electronics of the aromatic ring using a variety of electron-donating and -withdrawing substituents (3u–x), but no clear reactivity trends were apparent across this series.

Finally, reaction of a symmetrical diacid substrate 1y was evaluated in the presence of 2 equiv. 3-oxetanone (2) (Fig. 2b). The major product was mono-oxetanol 3z with protodecarboxylation observed at the second site, while only 10% of the di-oxetanol 3y was recovered.

### Development of Cr-free carboxylic acid-to-3-oxetanol transformation

We were intrigued by the discovery above that the photoredox-catalyzed decarboxylative addition reaction also proceeded effectively in the absence of CrCl_3_ and TMSCl (Table 1, entry 14). Thus, we investigated further optimization of this Cr-free reaction using *N*-phenyl valine (1f), because the Cr-mediated reaction provided a low yield for this substrate (Figure 2, 28%). Omitting CrCl_3_ and TMSCl from the Cr-mediated reaction conditions afforded only a 9% of the desired product (Table 2, entry 1; see ESI Table S2† for complete details). We noted that there was little difference in yield when the reaction time was shortened from 20 h to 2 h (entry 2), facilitating further evaluation of reaction conditions. Investigation of various stoichiometric ratios (entries 2–5) showed that increasing 4CzIPN catalyst loading to 2 mol% and 3-oxetanone to 3 equiv. provided a modest increase in yield to 13%. Evaluation of other solvents (entries 5–8) afforded a dramatic increase in yield in CH_2_Cl_2_ (89%). Bases other than CsOPiv (entries 9 and 10) and photocatalysts other than 4CzIPN (entries 11 and 12) were far less effective. The reaction also remained dependent upon light (entry 13). We note that König and coworkers have previously reported photocatalyzed decarboxylative additions of arylacetic acids to aldehydes under similar conditions.^14^ However, application of the literature conditions (4CzIPN, Cs_2_CO_3_, DMA, LED, rt, 16 h) to our substrate 1f did not afford any of the desired 3-oxetanol product 3f.

**Table 2:** Optimization of the Cr-free carboxylic acid-to-3-oxetanol transformation

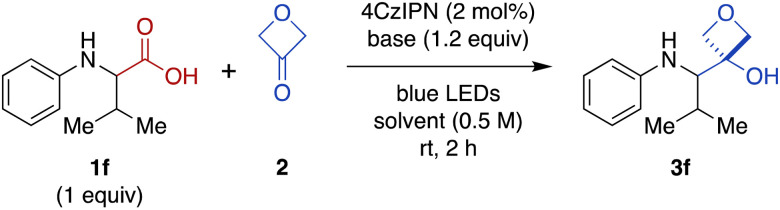				
Entry	2 (equiv.)	Base	Solvent	Yield^a^ (%)
1^b^^,^^c^	1.0	CsOPiv	CH_3_CN	9
2^b^	1.0	CsOPiv	CH_3_CN	8
3^b^	2.0	CsOPiv	CH_3_CN	10
4^b^	3.0	CsOPiv	CH_3_CN	11
5	3.0	CsOPiv	CH_3_CN	13
6	3.0	CsOPiv	DCE	57
7	3.0	CsOPiv	CH_2_Cl_2_	89
8	3.0	CsOPiv	i-PrOH	78
9	3.0	Na_2_CO_3_	CH_2_Cl_2_	24
10	3.0	DIPEA	CH_2_Cl_2_	21
11^d^	3.0	CsOPiv	CH_2_Cl_2_	43
12^e^	3.0	CsOPiv	CH_2_Cl_2_	30
13^f^	3.0	CsOPiv	CH_2_Cl_2_	0

a Yields based on ^1^H-NMR analysis of crude reaction product in the presence of an internal standard, relative to *N*-phenyl valine (1f).

b 1 mol% 4CzIPN.

c 20 h reaction time.

d Photocatalyst: Ir-A = [Ir{dF(CF_3_)_2_ppy}_2_(bpy)]PF_6_ = [2,2′-bipyridine-*N*1,*N*1′]bis[3,5-difluoro-2-[5-(trifluoromethyl)-2-pyridinyl-*N*]phenyl-*C*]iridium(iii) hexafluorophosphate.

e Photocatalyst: Ir-B = [Ir{dF(CF_3_)ppy}_2_(dtbpy)]PF_6_ = [4,4′-bis(1,1-dimethylethyl)-2,2′-bipyridine-*N*1,*N*1′]bis[3,5-difluoro-2-[5-(trifluoromethyl)-2-pyridinyl-*N*]phenyl-*C*]iridium(iii) hexafluorophosphate.

f In absence of blue LED light. DCE = 1,2-dichloroethane; DIPEA = *N*,*N*-diisopropylethylamine.

Next, we investigated the substrate scope of the Cr-free reaction across the panel of *N*-aryl α-amino acid substrates (Fig. 2). In most cases, the Cr-free reaction provided higher yields of the 3-oxetanol products compared to those observed with the Cr-mediated reaction, in some cases dramatically so (*e.g.*, 3d, 3f, 3g, 3l, 3q, 3s). Across the entire panel (3a–x), the average yield was 64% for the Cr-free reaction compared to 48% for the Cr-mediated reaction. In the case of the diacid substrate 1y, the Cr-free reaction provided the mono-oxetanol 3z exclusively. Overall, the Cr-free reaction provides significant advantages over the original Cr-mediated reaction with respect to efficiency (time, yield) and elimination of toxic and reactive reagents (CrCl_3_, TMSCl).

**Fig. 3:**
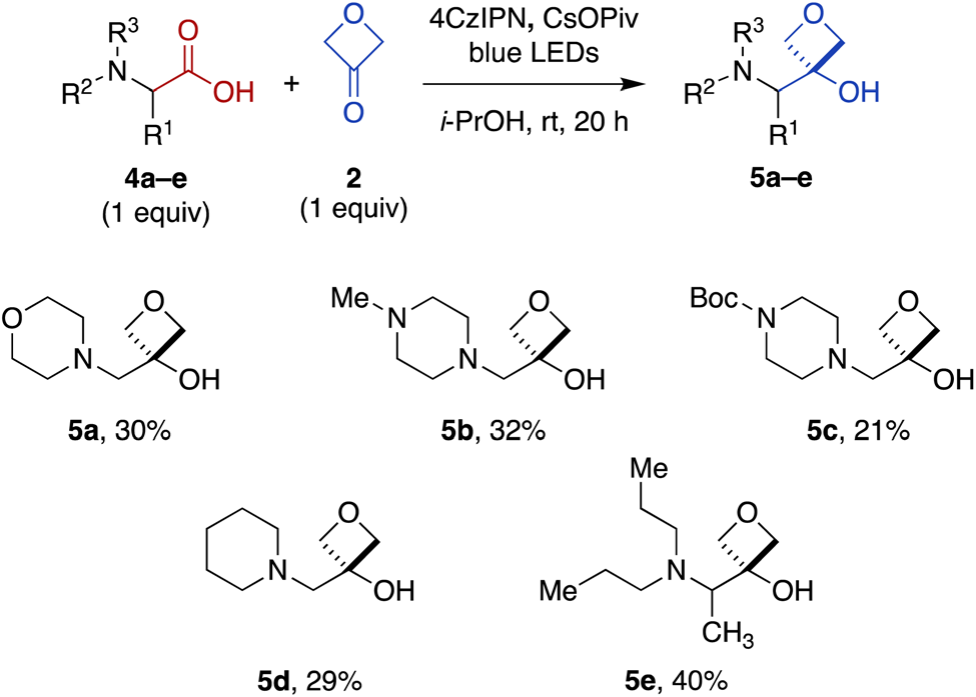
Direct carboxylic acid-to-3-oxetanol transformation for cyclic and acyclic *N*,*N*-dialkyl α-amino substrates under modified Cr-free reaction conditions.

To expand the scope of this transformation beyond *N*-aryl α-amino acid substrates, we investigated Cr-free reactions of other amino acids. In preliminary experiments, we found that exposure of primary (phenylalanine), secondary (*N*-trityl glycine), and *N*-acylated (*N*-Boc-glycine, *N*-Cbz-proline, *N*-phthaloylglycine) α-amino acids to the reaction conditions did not afford any of the desired 3-oxetanol products (not shown). However, morpholine acetic acid was converted to the desired product, albeit with some bis and tris modification observed by MS, presumably at the ring carbons α to the amine. Selectivity for monofunctionalization was improved by decreasing 3-oxetanone stoichiometry from 3 equiv. to 1 equiv. With other slight modifications (changing solvent from CH_2_Cl_2_ to i-PrOH to improve solubility; increasing reaction time to 20 h), the desired 3-oxetanol 5a was obtained in 30% yield (Fig. 3). The reaction was also effective for systems containing *N*-methylpiperazine (5b), *N*-Boc-piperazine (5c), piperidine (5d), and an acyclic tertiary amine (5e), demonstrating tolerance of heteroatoms, protecting groups, and both cyclic and acyclic substrates.

### Mechanistic investigations

Next, we probed the mechanisms of the Cr-mediated and Cr-free transformations. We considered three possible mechanisms *a priori*: (1) addition of an α-amino carbanion (or Nozaki–Hiyama–Kishi alkyl-Cr intermediate) to 3-oxetanone,^15,26^ (2) addition of an α-amino radical to 3-oxetanone,^39^ or (3) radical–radical recombination of an α-amino radical and 3-oxetanone-derived radical.^34^

**Fig. 4:**
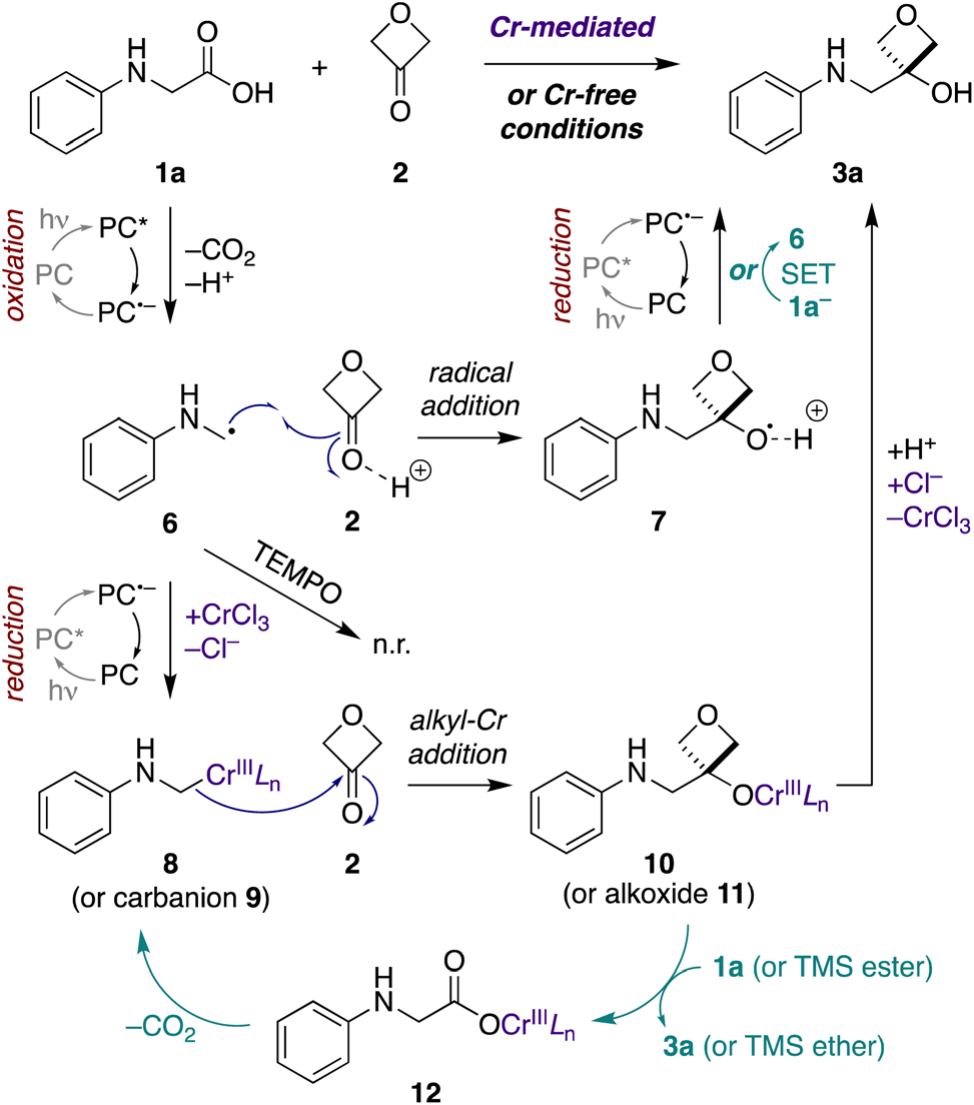
Possible mechanisms for Cr-mediated and Cr-free photoredox-catalyzed decarboxylative addition to 3-oxetanone (2). Initial photocatalytic oxidative decarboxylation of α-amino acid carboxylate 1a^−^ forms α-amino radical 6. Under Cr-mediated conditions (purple), reduction to alkyl-Cr species 8 predominates, with nucleophilic addition to 3-oxetanone (2) forming Cr alkoxide 10. Chain propagation (teal) may occur by conversion of Cr-alkoxide 10 to Cr-carboxylate 12*via* either direct proton–Cr exchange or σ-bond metathesis of the corresponding TMS ester of 1a, followed by decarboxylation to regenerate alkyl-Cr species 8. Under Cr-free conditions, direct radical addition of α-amino radical 6 to Brønsted-acid activated 3-oxetanone (2) predominates, forming radical cation 7. The reaction may terminate by photocatalyzed reduction of 7 to the product 3a. Alternatively, chain propagation (teal) may occur through an SET event between 7 and carboxylate 1a^−^ to furnish the product 3a and regenerate α-amino radical 6 (teal). A minor pathway in the Cr-mediated reaction may involve this same radical addition (6 + 2 → 7), while a minor pathway in the Cr-free reaction may involve a free carbanion/alkoxide mechanism (9 + 2 → 11). *L* = ligand, n.r. = no reaction, PC = photocatalyst, PC* = excited state, PC˙^−^ = radical anion state.

First, to assess the reactivity of each of the substrates and reagents to photoactivated 4CzIPN, we conducted fluorescence quenching studies with *N*-phenylglycine (cesium salt) (1a), 3-oxetanone (2), CsOPiv, TMSCl, and CrCl_3_.^40^ Stern–Volmer analysis revealed that the quenching constant of the carboxylate 1a was substantially greater than that of the other reagents in both CH_3_CN and CH_2_Cl_2_.^40^ This supports a pathway in which the carboxylate substrate 1a reacts with the excited photocatalyst to undergo oxidative decarboxylation, forming an α-amino radical intermediate 6 (Fig. 4). Consistent with this mechanism, no product formation was observed when either transformation was carried out in the presence of TEMPO (1 equiv.).

Next, to assess the possibility of a radical–radical recombination pathway (not shown), we measured the standard reduction potential (*E*_1/2_) of 3-oxetanone (2) using differential pulse voltammetry (DPV).^40^ We determined an *E*_1/2_ value of −2.51 V *vs.* SCE in CH_3_CN. In contrast, the redox potentials of 4CzIPN (*E*_1/2_[*P*˙^+^/P*] = −1.04 V; *E*_1/2_ [*P*/*P*˙^−^] = −1.21 V *vs.* SCE in CH_3_CN)^41^ are too small to drive reduction of 3-oxetanone (2) to the corresponding ketyl radical. Accordingly, radical–radical recombination pathways were ruled out for both reaction conditions.

In contrast, in the Cr-mediated reaction, the reduction potentials of 4CzIPN˙^−^ are sufficient to reduce Cr^III^L_n_ to Cr^II^L_n_ (*E*_1/2_ [Cr^III^/Cr^II^] = −0.51 V *vs.* SCE in DMF).^42^ This reduced Cr^II^L_n_ can then intercept the α-amino radical 6 to generate alkyl-Cr intermediate 8, a step that has been extensively investigated in Nozaki–Hiyama–Kishi reaction manifolds,^43^ which may then add to 3-oxetanone (2) to form Cr alkoxide 10. The reaction may then terminate by protonation to form oxetanol 3a. Alternatively, it is possible that α-amino radical 6 may undergo direct addition to Brønsted acid-activated 3-oxetanone (2) to form radical cation 7, and there is precedent for such 1,2-additions.^39,44^ Subsequently, photocatalyzed reduction of the radical cation 7 would form oxetanol 3a, also completing the photocatalytic cycle.

Thus, to investigate these two possibilities, we carried out deuterium quenching experiments using the parent substrate *N*-phenyl glycine (1a) with 3-oxetanone (2) and/or methanol-*d* (CH_3_OD) (Table 3). We anticipated that, in the presence of methanol, the alkyl-Cr intermediate 8, but not the corresponding α-amino radical species 6, would be quenched to form the proto(deutero)decarboxylation products 13 (ESI Figure S1†). Under the standard Cr-mediated reaction conditions, we observed 60% of the 3-oxetanol product 3a and 14% protodecarboxylation product 13a (Table 3, entry 1). When 3-oxetanone was omitted and replaced by methanol-*d*, the yield of the proto/deuterodecarboxylation products 13a, b increased to 59% (combined), with 80% deuterium incorporation (entry 2), consistent with the alkyl-Cr addition pathway. Interestingly, when both 3-oxetanone (2) and methanol-*d* were included in the reaction, yields of both the 3-oxetanol product 3a and the protodecarboxylation products 13a, b were decreased (entry 3), suggesting that additional undesired reaction pathways become active under these conditions.

**Table 3:** Competition experiments under Cr-mediated and Cr-free reaction conditions

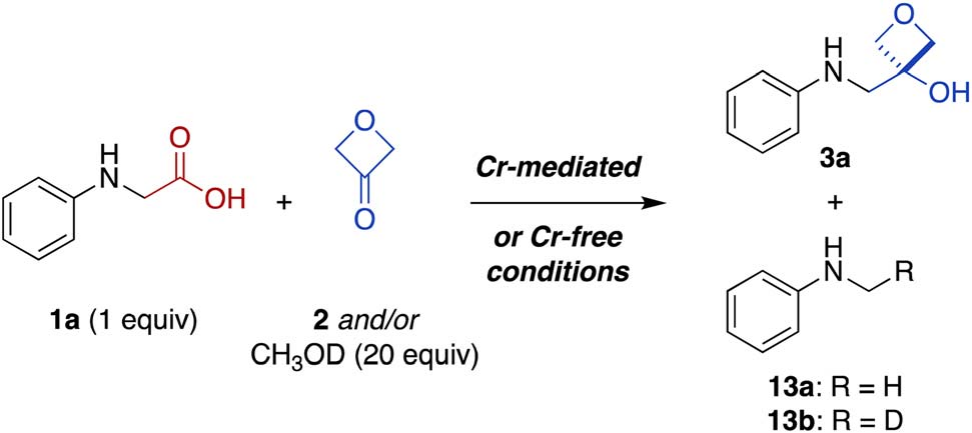					
Entry	Conditions^a^	Electrophile	Quencher	3a^b^ (%)	13a + 13b (%)
1	Cr-mediated	2 (1 equiv.)	—	60	14
2	Cr-mediated^c^	—	CH_3_OD	—	59 (80)^d^
3	Cr-mediated^c^	2 (1 equiv.)	CH_3_OD	48	6 (57)^d^
4	Cr-free	2 (3 equiv.)	—	60	5
5	Cr-free^c^	—	CH_3_OD	—	47 (55)^d^
6	Cr-free^c^	2 (3 equiv.)	CH_3_OD	100	—

a Cr-mediated reaction conditions: 1 mol% 4CzIPN, 5 mol% CrCl_3_, 0.5 equiv. TMSCl, 1.2 equiv. CsOPiv, 0.8 M in CH_3_CN based on amino acid substrate 1a, blue LED light, rt, 20 h. Cr-free reaction conditions: 2 mol% 4CzIPN, 1.2 equiv. CsOPiv, 0.5 M in CH_2_Cl_2_ based on amino acid substrate 1a, blue LED light, rt, 2 h.

b Yields based on ^1^H-NMR analysis of crude reaction product in the presence of an internal standard, relative to *N*-phenyl glycine (1a).

c Amino acid substrate 1a was deuterium exchanged with CH_3_OD prior to the reaction.

d Percent deuterium incorporation (13b: R = D) shown in parentheses.

In the Cr-free reaction, quenching with methanol-*d* also resulted in formation of the proto/deuterodecarboxylation products 13a, b (entry 5), consistent with formation of an α-amino carbanion intermediate 9 (Fig. 4 and ESI Figure S1†). In contrast, when both 3-oxetanone (2) and methanol-*d* were included in the reaction, the yield of the 3-oxetanol product 3a increased to 100% (entry 6). This is contrary to expectation if the standard Cr-free reaction proceeds solely *via* a carbanion intermediate. Notably, Glorius and coworkers have proposed that photoredox-initiated intermolecular radical trapping by ketones and aldehydes may be promoted by Brønsted-acid activation of the carbonyl compound.^39^ Thus, the increased yield observed under these conditions (entries 3 and 6) may be attributed to such activation of 3-oxetanone by methanol. Unfortunately, the improved yield observed in Cr-free reaction in the presence of methanol did not prove generalizable to other *N*-aryl α-amino acid substrates (not shown).

The contrasting results in these competition experiments, in which the reaction conditions are significantly perturbed by omission of the electrophile or addition of a cosolvent, make it difficult to draw definitive conclusions regarding the predominant pathways under the standard Cr-mediated and Cr-free reaction conditions, and suggest that both are possible.

Lastly, we investigated the quantum yields of these transformations. Photon flux of the light source was determined using standard ferrioxalate actinometry.^40^ The quantum yield was then calculated by determining the amount of product formed in 3 min under the standard reaction conditions, and dividing by the photon flux. We observed quantum yields of 5.2 for the Cr-mediated reaction and 10.3 for the Cr-free reaction, indicative of chain propagation mechanisms under both conditions.

In the context of the Cr-mediated reaction, the reduction potentials of carboxylic acid 1a (*E*_1/2_ [1a^+^/1a] = +0.42 V *vs.* SCE in CH_3_CN)^36^ and Cr^III^L_n_ (*E*_1/2_[Cr^III^/Cr^II^] = −0.51 V *vs.* SCE in DMF)^42^ indicate that direct oxidative decarboxylation of 1a by Cr(iii) would be thermodynamically unfavorable, making chain propagation *via* a redox mechanism unlikely.

An alternative possibility is that the alkyl-Cr species 8 is regenerated *via* a cycle in which the Cr-alkoxide intermediate 10 reacts with a new equivalent of the carboxylic acid substrate 1a to form Cr-carboxylate 12, which then undergoes metal-mediated decarboxylation to form alkyl-Cr species 8.^45,46^ Formation of Cr-carboxylate 12 could occur either *via* direct proton–Cr exchange with carboxylic acid 1a, or *via* σ-bond metathesis with the corresponding TMS ester, as postulated by Glorius and coworkers in related propagation reactions with trimethylsilylmethylamines,^15^ with subsequent desilylation of the resultant TMS ether to the product 3a. Consistent with the latter hypothesis, when TMSCl was omitted from the reaction, the quantum yield dropped to 1.6, indicating an important role in the propagation cycle.

In the Cr-free reaction, chain propagation may occur *via* SET between radical cation 7 and carboxylate 1a^−^ (*E*_1/2_ [1a˙/1a^−^] = +0.42 V *vs.* SCE in CH_3_CN)^36^ to regenerate α-amino radical 6 and furnish 3-oxetanol product 3a. This electron transfer event should be thermodynamically favorable, based on the computationally determined redox potential of an alkoxy radical cation-to-alcohol conversion by Glorius and coworkers.^39^

Taken together, these results suggest that Cr-mediated reaction proceeds predominantly *via* the alkyl-Cr addition pathway (8 + 2 → 10), because omission of TMSCl results in a large decrease in quantum yield (5.2 to 1.6), indicating the importance of the Cr-based chain propagation cycle (10 → 12 → 8) compared to the SET chain propagation cycle (7 → 6) (ESI Fig. S2†). In contrast, the Cr-free reaction cannot involve the Cr-based chain propagation cycle (and the free carboxylate analogue of 12 would not decarboxylate spontaneously to form carbanion 9). Thus, the high quantum yield in that reaction (10.3) must be attributed to the SET propagation cycle, which can only arise from the radical addition pathway (6 + 2 → 7). Thus, while both reaction manifolds may be operative to some extent under both conditions, it appears that the Cr-mediated reaction proceeds mainly *via* the alkyl-Cr pathway and the Cr-free reaction proceeds mainly *via* the radical addition pathway.

## Conclusions

In summary, by leveraging photoredox catalysis, we have successfully developed a method for direct conversion of α-amino acids to bioisosteric 3-oxetanols, thus avoiding the lengthy *de novo* synthesis approaches that have been used previously to access such motifs. Mechanistic investigations support a pathway involving initial oxidative decarboxylation to an α-amino radical species, which can then undergo direct radical addition to 3-oxetanone, or intermediate reduction to an α-amino alkyl-Cr or carbanion species followed by nucleophilic addition to 3-oxetanone, with the dominant reaction manifold dictated by the presence or absence of Cr. Notably, in both cases, chain propagation provides quantum yields >5. This methodology is applicable to a wide range of *N*-aryl α-amino acids, a motif which has been reported to have a variety of potential therapeutic applications in infectious disease, inflammation, neurodegeneration, and metabolic and gastrointestinal diseases.^47–49^ The substrate scope of the Cr-free reaction also includes *N*,*N*-dialkyl α-amino acid substrates. Efforts to expand the substrate scope further to other carboxylic acids are under active investigation in our lab. This direct conversion of carboxylic acids to 3-oxetanols should facilitate further investigation of these attractive bioisosteres in medicinal chemistry.

## Author contributions

A. M. V. D. R., C. S. N. E., A. M., and D. S. T. conceptualized the experiments; A. M. V. D. R. and C. S. N. E. performed the experiments with assistance from M. I. H.; A. M. V. D. R. and D. S. T. prepared the manuscript; A. M. V. D. R., C. S. N. E., A. M., and D. S. T. edited the manuscript.

## Conflicts of interest

There are no conflicts to declare.

## Supplementary Material

SC-014-D3SC00936J-s001
