# Supplementary material for: Direct conversion of amino acids to oxetanol bioisosteres via photoredox catalysis
Source: Chem Sci. 2023 Sep 15;14(38):10524–31. doi: 10.1039/d3sc00936j (PMC10548506; doi:10.1039/d3sc00936j)
Supplement: SC-014-D3SC00936J-s001 [file SC-014-D3SC00936J-s001.pdf]

Electronic Supplementary Material (ESI) for Chemical Science.
This journal is © The Royal Society of Chemistry 2023

Direct Conversion of Amino Acids to Oxetanol Bioisosteres via Photoredox Catalysis

Avelyn Mae V. Delos Reyes,[a] Christopher S. Nieves Escobar,[b]
Alberto Muñoz,[c] Maya I. Huffman,[d] and Derek S. Tan [a,b,c,d,e*]

[a]*Pharmacology Graduate Program, Weill Cornell Graduate School of Medical Sciences,*
[b]*Tri-Institutional PhD Program in Chemical Biology*,
[c]*Chemical Biology Program, Sloan Kettering Institute,*
[d]*Tri-Institutional Chemical Biology Summer Program, and*
[e]*Tri-Institutional Research Program,*
Memorial Sloan Kettering Cancer Center,
New York, New York 10065, United States

Supporting Information

A. Supplementary Tables S1 and S2 and Supplementary Figures S1 and S2 S2
B. Materials and Methods S8
C. Synthesis of *N*-Aryl-α-Amino Acids (**1c–g,l–t,x**) S10
D. Conversion of *N*-Aryl-α-Amino Acids (**1**) to 3-Oxetanols (**3**) S15
E. Conversion of *N*,*N*-Dialkyl-α-Amino Acids (**4**) to 3-Oxetanols (**5**) S24
F. Mechanistic Studies
  1. Deuterium quenching and competition experiments S26
  2. Differential pulse votammetry (DPV) measurements of 3-oxetanone S27
  3. Stern–Volmer fluorescence quenching analysis S28
  4. Quantum yield determination S30
G. Supporting Information References S33
H. ^{1}H-NMR and ^{13}C-NMR Spectra S34

A. SUPPLEMENTARY TABLES S1 & S2 AND SUPPLEMENTARY FIGURES S1 & S2

Supplementary Table S1. Complete Data on Optimization of Cr-mediated Reaction

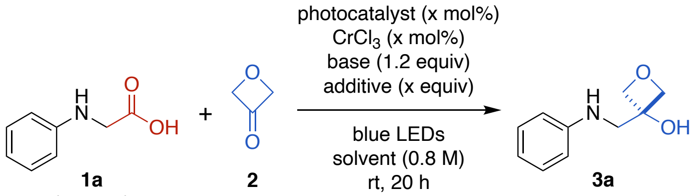

1a (1 equiv) **2** (0.5 equiv) **3a**

entry	photocatalyst	base	additive	Cr catalyst	solvent	yield (%)[a]
1	4CzIPN (1 mol%)	–	–	$CrCl_3$ (5 mol%)	DMA	0
2	4CzIPN (1 mol%)	CsOAc	–	$CrCl_3$ (5 mol%)	DMA	7
3	4CzIPN (1 mol%)	CsOAc	TMSCl (0.5 equiv)	$CrCl_3$ (5mol%)	DMA	22
4	4CzIPN (1 mol%)	CsOAc	TMSCl (0.5 equiv)	$CrCl_3$ (5 mol%)	DMF	25
5	4CzIPN (2.5 mol%)	CsOAc	TMSCl (0.5 equiv)	$CrCl_3$ (5 mol%)	DMF	22
6	4CzIPN (5 mol%)	CsOAc	TMSCl (0.5 equiv)	$CrCl_3$ (5 mol%)	DMF	25
7	[Ir(dF(CF$_3$)ppy)$_2$(dtbbpy)] [PF$_6$] (1 mol%)	CsOAc	TMSCl (0.5 equiv)	$CrCl_3$ (5 mol%)	DMF	17
8	[Ir(dF(CF$_3$)$_2$ppy)$_2$(bpy)] [PF6] (1 mol%)	CsOAc	TMSCl (0.5 equiv)	$CrCl_3$ (5 mol%)	DMF	24
9	4CzIPN (1 mol%)	CsOAc	TMSCl (0.5 equiv)	$CrCl_3$ (10 mol%)	DMF	25
10	4CzIPN (1 mol%)	CsOAc	TMSCl (0.5 equiv)	$CrCl_3$ (25 mol%)	DMF	0
11	4CzIPN (1 mol%)	CsOAc	TMSCl (0.5 equiv)	$CrCl_3$ (0.5 equiv)	DMF	0
12	4CzIPN (1 mol%)	CsOAc	TMSCl (0.25 equiv)	$CrCl_3$ (5 mol%)	DMF	18
13	4CzIPN (1 mol%)	CsOAc	TMSCl (1 equiv)	$CrCl_3$ (5 mol%)	DMF	0
14	4CzIPN (1 mol%)	CsOAc	TMSCl (2 equiv)	$CrCl_3$ (5 mol%)	DMF	0
15	4CzIPN (1 mol%)	NaOAc	TMSCl (0.5 equiv)	$CrCl_3$ (5 mol%)	DMF	29
16	4CzIPN (1 mol%)	LiOAc	TMSCl (1 equiv)	$CrCl_3$ (10 mol%)	DMF	25

entry	photocatalyst	base	additive	Cr catalyst	solvent	yield (%)[a]
17	4CzIPN (1 mol%)	KOAc	TMSCl (0.5 equiv)	$CrCl_3$ (5 mol%)	DMF	27
18	4CzIPN (1 mol%)	Cs_2CO_3	TMSCl (0.5 equiv)	$CrCl_3$ (5 mol%)	DMF	0
19	4CzIPN (1 mol%)	Li_2CO_3	TMSCl (0.5 equiv)	$CrCl_3$ (5 mol%)	DMF	0
20	4CzIPN (1 mol%)	CsOPiv	TMSCl (0.5 equiv)	$CrCl_3$ (5 mol%)	DMF	33
21	4CzIPN (1 mol%)	$NaHCO_3$	TMSCl (0.5 equiv)	$CrCl_3$ (5 mol%)	DMF	11
22	4CzIPN (1 mol%)	$KHCO_3$	TMSCl (0.5 equiv)	$CrCl_3$ (5 mol%)	DMF	8
23	4CzIPN (1 mol%)	$NaCO_2C_2H_5$	TMSCl (0.5 equiv)	$CrCl_3$ (5 mol%)	DMF	21
24	4CzIPN (1 mol%)	NaBz	TMSCl (0.5 equiv)	$CrCl_3$ (5 mol%)	DMF	19
25	4CzIPN (1 mol%)	$NaCO_2C_3H_7$	TMSCl (0.5 equiv)	$CrCl_3$ (5 mol%)	DMF	19
26	4CzIPN (1 mol%)	$CsCO_2Ad$	TMSCl (0.5 equiv)	$CrCl_3$ (5 mol%)	DMF	31
27	4CzIPN (1 mol%)	DBU	TMSCl (0.5 equiv)	$CrCl_3$ (5 mol%)	DMF	0
28	4CzIPN (1 mol%)	2,6-lutidine	TMSCl (0.5 equiv)	$CrCl_3$ (5 mol%)	DMF	0
29	4CzIPN (1 mol%)	CsOPiv	TMSCl (0.5 equiv)	$CrCl_3$ (5 mol%)	CH_3CN	40
30	4CzIPN (1 mol%)	CsOPiv	TMSCl (0.5 equiv)	$CrCl_3$ (5 mol%)	THF	38
31	4CzIPN (1 mol%)	CsOPiv	TMSCl (0.5 equiv)	$CrCl_3$ (5 mol%)	DMSO	23
32	4CzIPN (1 mol%)	CsOPiv	TMSCl (0.5 equiv)	$CrCl_3$ (5 mol%)	DMA	29
33	4CzIPN (1 mol%)	CsOPiv	TMSCl (0.5 equiv)	$CrCl_3$ (5 mol%)	DCM	33
34	4CzIPN (1 mol%)	CsOPiv	TIPSCl (0.5 equiv)	$CrCl_3$ (5 mol%)	CH_3CN	37
35	4CzIPN (1 mol%)	CsOPiv	TESCl (0.5 equiv)	$CrCl_3$ (5 mol%)	CH_3CN	37
36	4CzIPN (1 mol%)	CsOPiv	TESOTf (0.5 equiv)	$CrCl_3$ (5 mol%)	CH_3CN	38
37	4CzIPN (1 mol%)	CsOPiv	TMSCl (0.5 equiv)	–	CH_3CN	32

entry	photocatalyst	base	additive	Cr catalyst	solvent	yield (%)[a]
38	4CzIPN (1 mol%)	CsOPiv	–	–	CH_3CN	25
39	4CzIPN (1 mol%)	CsOPiv	TMSCl (0.5 equiv)	$CrCl_3$ (5 mol%)	CH_3CN	60[b]
40	4CzIPN (1 mol%)	CsOPiv	TMSCl (0.5 equiv)	$CrCl_3$ (5 mol%)	CH_3CN	55[c]
41	4CzIPN (1 mol%)	CsOPiv	TMSCl (0.5 equiv)	$CrCl_3$ (5 mol%)	CH_3CN	50[d]
42	4CzIPN (1 mol%)	CsOPiv	TMSCl (0.5 equiv)	–	CH_3CN	50[b]
43	4CzIPN (1 mol%)	CsOPiv	–	$CrCl_3$ (5 mol%)	CH_3CN	49[b]
44	4CzIPN (1 mol%)	–	TMSCl (0.5 equiv)	$CrCl_3$ (5 mol%)	CH_3CN	0[b]
44	4CzIPN (1 mol%)	CsOPiv	–	–	CH_3CN	47[b]
45	4CzIPN (1 mol%)	CsOPiv	TMSCl (0.5 equiv)	$CrCl_3$ (5 mol%)	CH_3CN	42 [b,e]
46	4CzIPN (1 mol%)	CsOPiv	TMSCl (0.5 equiv)	$CrCl_3$ (5 mol%)	CH_3CN	0[b,f]
47	–	CsOPiv	TMSCl (0.5 equiv)	$CrCl_3$ (5 mol%)	CH_3CN	0[b]

[a]Yields based on ^{1}H-NMR analysis of crude reaction product and are relative to *N*-phenyl glycine. [b]1 equiv *N*-phenyl glycine to 1 equiv 3-oxetanone. [c]1 equiv *N*-phenyl glycine to 2 equiv 3-oxetanone. [d]1 equiv *N*-phenyl glycine to 3 equiv 3-oxetanone. [e]Reaction mixture was not degassed with Ar. [f]Reaction mixture was not irradiated with blue LEDs.

Supplementary Table S2. Complete Data on Optimization of Cr-free Reaction

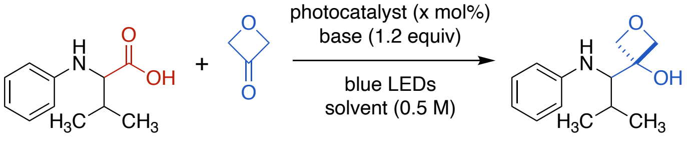

entry	3-oxetanone (equiv)	photocatalyst	base (1.2 equiv)	solvent	yield (%)[a]
1	1	4CzIPN (1 mol%)	CsOPiv	CH_3CN	15[b]
2	1	4CzIPN (1 mol%)	CsOPiv	CH_3CN	6[c]
3	1	4CzIPN (1 mol%)	CsOPiv	CH_3CN	9[d]
4	1	4CzIPN (1 mol%)	CsOPiv	CH_3CN	8
5	2	4CzIPN (1 mol%)	CsOPiv	CH_3CN	10
6	3	4CzIPN (1 mol%)	CsOPiv	CH_3CN	11
7	3	4CzIPN (2 mol%)	CsOPiv	CH_3CN	13
8	3	4CzIPN (2 mol%)	CsOPiv	DCE	57
9	3	4CzIPN (2 mol%)	CsOPiv	CH_2Cl_2	89
10	3	4CzIPN (2 mol%)	CsOPiv	iPrOH	78
11	3	4CzIPN (2 mol%)	CsOPiv	DMF	0
12	3	4CzIPN (2 mol%)	CsOPiv	toluene	82
13	3	4CzIPN (2 mol%)	Na_2CO_3	CH_2Cl_2	24
14	3	4CzIPN (2 mol%)	DIPEA	CH_2Cl_2	21
15	3	5CzBN (2 mol%)	CsOPiv	CH_2Cl_2	66
16	3	3CzCl-IPN (2 mol%)	CsOPiv	CH_2Cl_2	63
17	3	3DPACl-IPN (2 mol%)	CsOPiv	CH_2Cl_2	50
18	3	3DPAF-IPN (2 mol%)	CsOPiv	CH_2Cl_2	74
19	3	3DPA2FBN (2 mol%)	CsOPiv	CH_2Cl_2	31
20	3	10-Phen-MesAcr (2 mol%)	CsOPiv	CH_2Cl_2	30
21	3	2,7-dimethyl-10-Phen-MesAcr (2 mol%)	CsOPiv	CH_2Cl_2	55
22	3	10-Me-MesAcr (2 mol%)	CsOPiv	CH_2Cl_2	5
23	3	$[Ir\{dF(CF_3)_2ppy\}_2(bpy)]PF_6$ (2 mol%)	CsOPiv	CH_2Cl_2	43
24	3	$[Ir\{dF(CF_3)_2ppy\}_2(dtbbpy)]PF_6$ (2 mol%)	CsOPiv	CH_2Cl_2	30

25	3	[Ir(dtbbpy)(ppy)$_2$]PF$_6$ (2 mol%)	CsOPiv	CH_2Cl_2	63
26	3	[Ir{dF(Me)$_2$ppy}2(dtbbpy)]PF$_6$ (2 mol%)	CsOPiv	CH_2Cl_2	62
27	3	fac-[Ir(dFppy)$_3$] (2 mol%)	CsOPiv	CH_2Cl_2	56
28	3	fac-[Ir(dppy)$_3$] (2 mol%)	CsOPiv	CH_2Cl_2	50
29	3	4CzMeBN (2 mol%)	CsOPiv	CH_2Cl_2	80

[a]Determined by ^{1}H-NMR analysis using mesitylene as an internal standard. [b]1.0 equiv of TMSCl was used as an additive. Reaction time = 20 h. [c]1.0 equiv of $CrCl_3$ was used as an additive. [d]Reaction time = 20 h. DCE = dichloroethane, DMF = *N*,*N*-dimethylformamide.

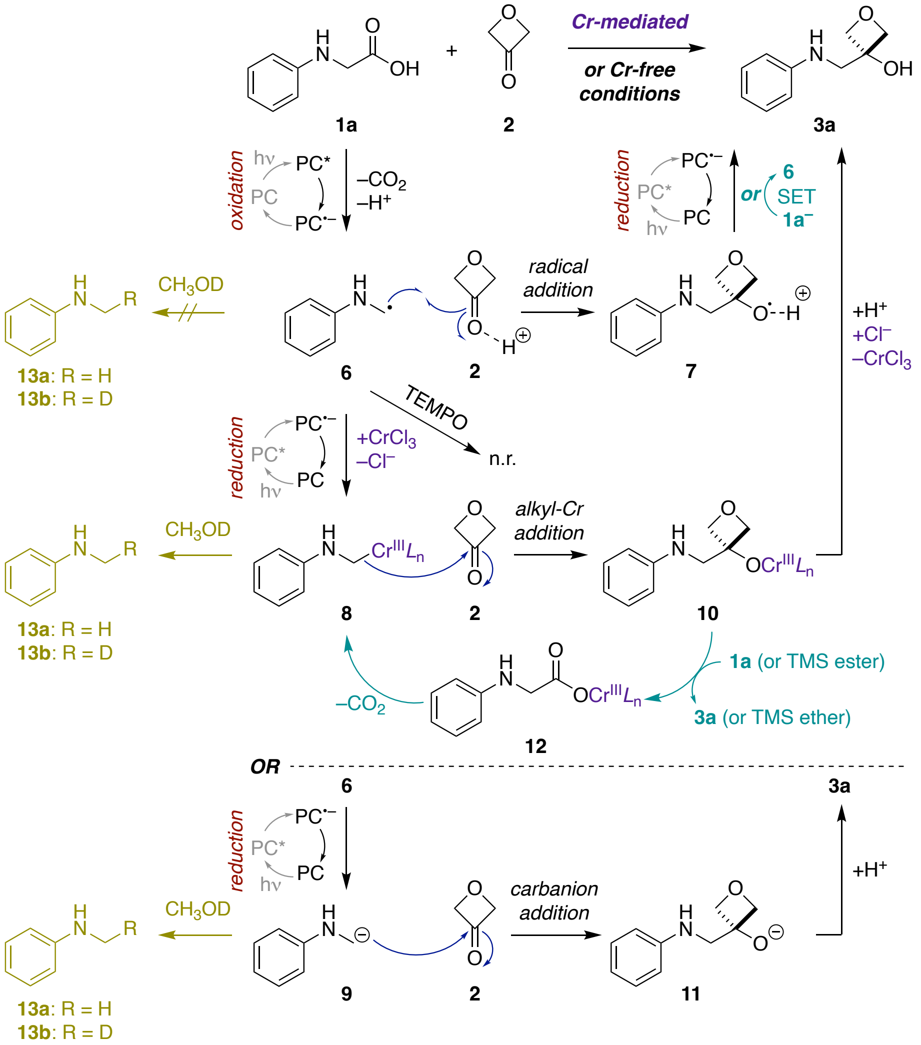

Supplementary Figure S1. Competition experiments with CH_3OD (olive). The alkyl-Cr (**8** + **2** → **10**) and carbanion (**9** + **2** → **11**) pathways are shown separately for clarity.

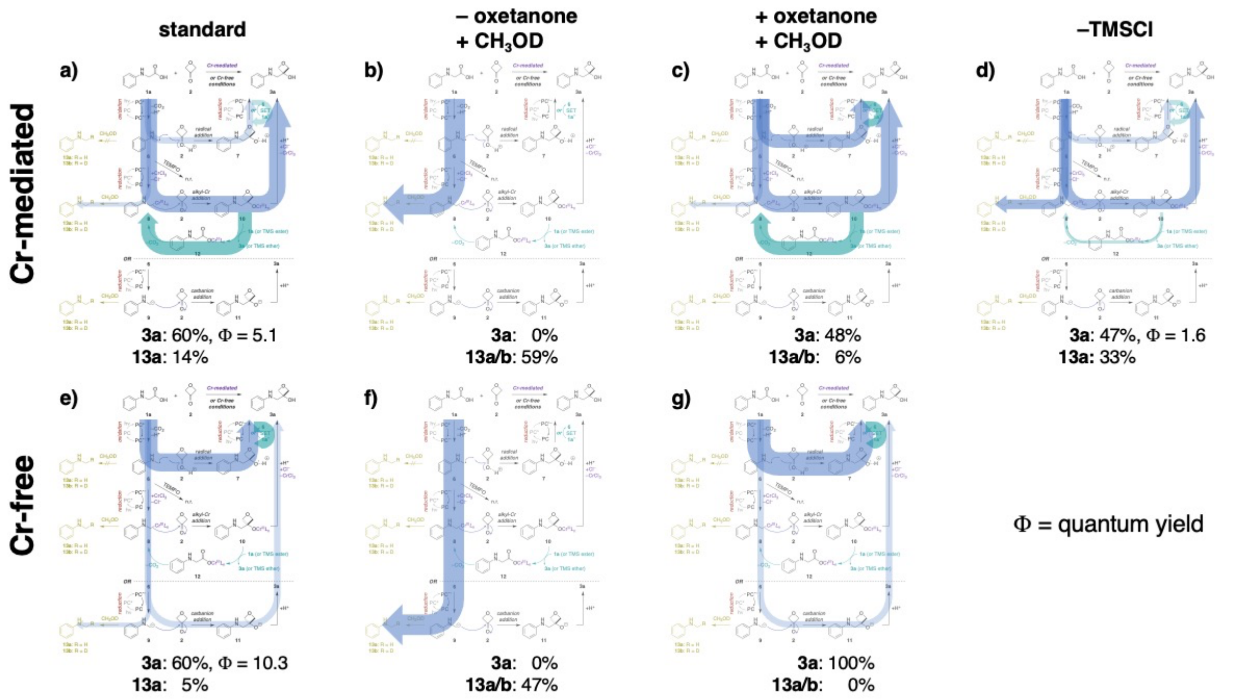

Supplementary Figure S2. Proposed flux through competing reaction pathways, consistent with the experimental data. See **Supplementary Figure S1** above for full-scale reaction scheme. Reaction arrows are in blue; propagation cycles are in teal; relative flux is represented by arrow size. **(a)** The Cr-mediated reaction proceeds primarily through the alkyl-Cr addition pathway (**8** + **2** → **10**), including the propagation cycle via Cr-carboxylate **12**. **(b)** In the presence of methanol but absence of oxetanone, only the protodecarboxylation products **13a/b** can be formed. **(c)** In the presence of both methanol and oxetanone, flux increases through the radical pathway, thus paradoxically decreasing protodecarboxylation to **13a/b**. **(d)** In the absence of TMSCl, the Cr-dependent propagation cycle is blocked or decreased, thus decreasing the quantum yield (Φ = 1.6 vs 5.1 with TMSCl); propagation may still occur to some extent via TMSCl-independent formation of Cr-carboxylate **12** and/or by the SET propagation cycle in the radical addition pathway. **(e)** The Cr-free reaction proceeds primarily through the radical addition pathway (**6** + **2** → **7**), including the SET propagation cycle that regenerates radical **6**. **(f)** In the presence of methanol but absence of oxetanone, the radical intermediate **6** has no productive pathway forward other than to shunt to photocatalyzed reduction to carbanion intermediate **9** and then to protodecarboxylation products **13a/b**. **(g)** In the presence of both methanol and oxetanone, flux increases through the radical addition pathway, resulting in increased yield of the 3-oxetanol product **3a**.

B. Materials and Methods

Reagents

Reagents were obtained from Aldrich Chemical (www.sigma-aldrich.com) or Acros Organics/Tokyo Chemical Industry (www.fishersci.com) and used without further purification. Commercially available α-amino acids were obtained from the following suppliers and used without further purification: *N*-phenyl glycine (**1a**) from Sigma Aldrich (www.sigmaaldrich.com); (2-(phenylamino)propanoic acid (**1b**), 1-phenylpyrrolidine-2-carboxylic acid (**1h**), 2-(2,3-dihydro-1*H*-indol-1-yl)acetic acid hydrochloride) (**1i**), 2-methyl-2-(phenylamino)propanoic acid (**1k**), 2-[(4-methylphenyl)amino]acetic acid (**1u**), *N*-(4-bromophenyl)-glycine (**1v**), and 2-[(carboxymethyl)-(phenyl) amino] acetic acid (**1y**) from Enamine (www.enamine.net); *N*-(4-fluorophenyl)-glycine (**1w)** and from Fisher Scientific (www.fishersci.com); *N*-methyl *N*-phenylalanine (**1j**), morpholin-4-yl acetic acid (**4a**), (4-methyl-piperazin-1-yl acetic acid (**4b**), Boc-(4-carboxymethyl)piperazine (**4c**), and piperidin-1-yl-acetic acid hydrochloride (**4d**), from Matrix Scientific (www.matrixscientific.com); *N*,*N*-dipropyl-L-alanine (**4e**) from Tokyo Chemical Industry (https://www.tcichemicals.com/US/en/). Optima or HPLC grade solvents were obtained from Fisher Scientific (www.fishersci.com), degassed with Ar, and purified on a solvent drying system as described unless otherwise indicated.[1]

Reactions

All reactions were performed in flame-dried glassware under positive Ar pressure with magnetic stirring unless otherwise noted. Liquid reagents and solutions were transferred thru rubber septa via syringes flushed with Ar prior to use.

Photochemical reactions were performed using a PR160 Rig with fan kit and irradiated with four PR160L LEDs (40 W, λ_{max} = 456 nm) from Kessil (www.kessil.com). This apparatus was enclosed within an aluminum covered box. The reaction vials were placed 5 cm from the LEDs and the temperature was measured to be between 25 °C to 30 °C using this configuration.

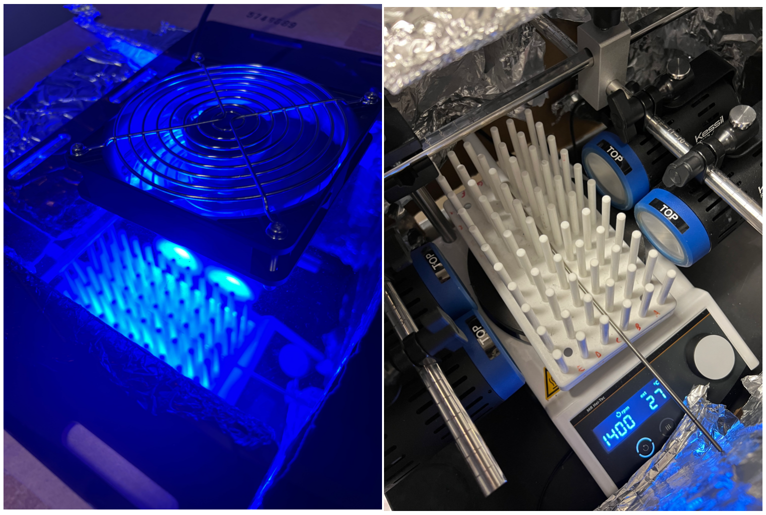

Supplementary Figure S2. Photochemical reaction apparatus using Kessil PR160L LEDs (40 W, λ_{max} = 456 nm) Rig and fan kit.

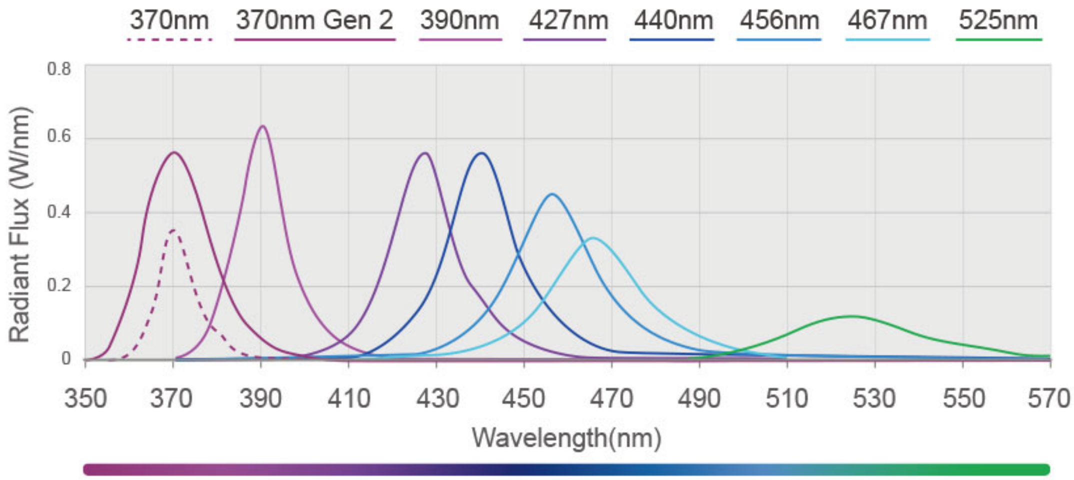

Supplementary Figure S3. Emission spectrum and intensity provided by the manufacturer, Kessil. Model PR160L 456 nm used for the reactions described.

Chromatography

TLC was performed on 0.25 mm E. Merck silica gel 60 F254 plates and visualized under UV light (254 nm) or by staining with potassium permanganate ($KMnO_4$), or cerium ammonium molybdenate (CAM). Silica flash chromatography was performed manually on E. Merck 230– 400 mesh silica gel 60 or on an ISCO CombiFlash Rf+ instrument with RediSep silica gel normal phase columns or RediSep Gold silica gel normal phase columns with UV detection at 254 nm.

Analytical Instrumentation

NMR spectra were recorded on a Bruker UltraShield Plus 500 MHz Avance III NMR or UltraShield Plus 600 MHz Avance III NMR with DCH CryoProbe at 24 °C in $CDCl_3$ unless otherwise indicated. Chemical shifts are expressed in ppm relative to TMS (^{1}H, 0 ppm) or solvent signals: $CDCl_3$ (^{13}C, 77.0 ppm), C_6D_6 (^{1}H, 7.16 ppm; ^{13}C, 128.0 ppm) or acetone-d_6 (^{13}C, 206.2 ppm); coupling constants are expressed in Hz. NMR spectra were processed using Bruker TopSpin, Mnova (www.mestrelab.com/software/mnova-nmr), or nucleomatica iNMR (www.inmr.net) software. Differential pulse voltammetry experiments were recorded using a 660E potentiostat/galvanostat from CH Instruments (https://www.chinstruments.com/). Mass spectra were obtained at the MSKCC Analytical Core Facility on a Waters Acuity SQD LC-MS. High resolution mass spectra were obtained on a Waters Acuity Premiere XE TOF LC-MS by electrospray ionization (ESI).

C. SYNTHESIS OF *N*-ARYL-α-AMINO ACIDS (1c–g,l–t,x)

N-Aryl α-amino acids **1c–g**, **1l–t**, and **1x**, which were not commercially available, were synthesized using a literature protocol[2] with the minor modification of running the reaction at 60 °C instead of rt.

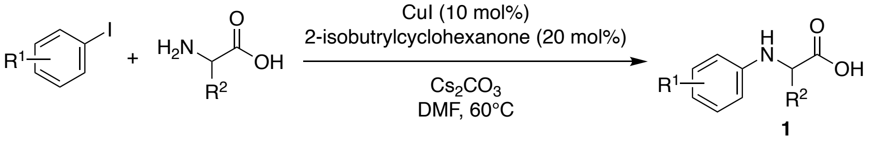

General Procedure for Ullmann Coupling

In a 50-mL roundbottom flask, CuI (23 mg, 0.25 mmol, 10 mol%), Cs_2CO_3 (1.63 g, 5 mmol, 2 equiv), and the appropriate α-amino acid (3 mmol, 1.2 equiv) were combined. The mixture was evacuated and refilled with Ar three times, then dissolved in DMF (3.7 mL). The appropriate aryl iodide (2.5 mmol, 1 equiv) and 2-isobutyrylcyclohexanone (0.82 mL, 0.5 mmol, 20 mol%) were added. The reaction mixture was heated to 60 °C and stirred until complete conversion as determined by TLC and ^{1}H-NMR analysis. The mixture was allowed to cool to rt, then diluted with 1*N* HCl to pH = 4, and extracted with 25 mL EtOAc (3x). The combined organic extracts were washed with 30 mL of water (2x) and brine, dried (Na_2SO_4), filtered, and concentrated by rotary evaporation. Purification by silica flash chromatography (10–30% EtOAc in hexanes, with 1% AcOH) afforded the *N*-aryl α-amino acid product **1**.

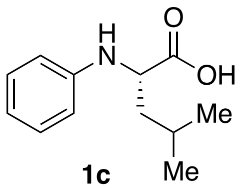

1c

***N*-Phenyl-L-leucine (1c)**: Prepared from iodobenzene and L-leucine in 78% yield as a white solid. The NMR spectra of **1c** matched those reported in literature.[2]

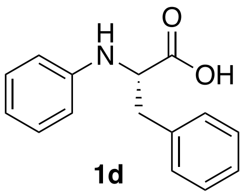

1d

***N*-Phenyl-L-phenylalanine (1d)**: Prepared from iodobenzene and L-phenylalanine in 66% yield as a white solid. The NMR spectra of **1d** matched those reported in literature.[2]

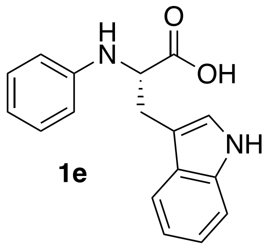

1e

***N*-Phenyl-L-tryptophan (1e)**: Prepared from iodobenzene and L-tryptophan in 51% yield as a brown solid. The NMR spectra of **1e** matched those reported in literature.[2]

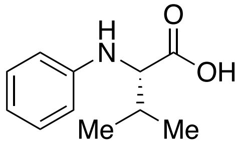

1f

***N*-Phenyl-L-valine (1f)**: Prepared from iodobenzene and L-valine in 72% yield as a light yellow solid. The NMR spectra of **1e** matched those reported in literature.[2]

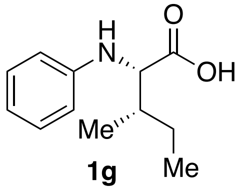

1g

***N*-Phenyl-L-isoleucine (1g)**: Prepared from iodobenzene and L-isoleucine in 70% yield as a light yellow solid. The NMR spectra of **1f** matched those reported in literature.[2]

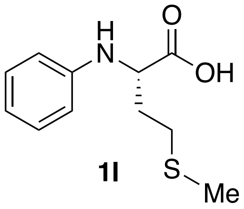

1l

***N*-Phenyl-L-methionine (1l)**: Prepared from iodobenzene and L-methionine in 78% yield as an off-white solid. The NMR spectra of **1l** matched those reported in literature.[2]

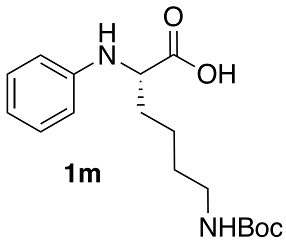

1m

***N*^{6}-(*tert*-butoxycarbonyl)-*N*^{2}-phenyl-L-lysine (1m)**: Prepared from iodobenzene and *N*^{6}-(*tert*-butoxycarbonyl)-L-lysine in 92% yield as an off-white solid. The NMR spectra of **1m** matched those reported in literature.[3]

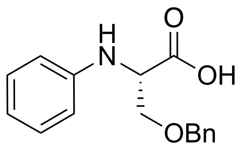

1n

***O*-benzyl-*N*-phenyl-L-serine (1n)**: Prepared from iodobenzene and *O*-benzyl-L-serine in 45% yield as an light yellow solid. **TLC**: R_f 0.30 (1% acetic acid in 2:3 EtOAc/Hexanes). **^{1}H NMR** (600 MHz, CD_2Cl_2) δ 7.39 – 7.27 (m, 5H), 7.23 – 7.15 (m, 2H), 6.84 – 6.75 (m, 1H), 6.74 – 6.60 (m, 2H), 4.57 (s, 2H), 4.20 (s, 1H, N–H), 3.96 (dd, *J* = 9.5, 4.0 Hz, 1H), 3.83 (dd, *J* = 9.5, 4.2 Hz, 1H). **^{13}C NMR** (151 MHz, CD_2Cl_2) δ 146.7, 137.8, 129.7, 128.9, 128.2, 119.6, 114.2, 73.8, 70.1, 57.9. **ESI-MS**: Calcd for $C_{16}H_{18}NO_3$ ($[M+H]^+$) 272.1287; found 272.1284.

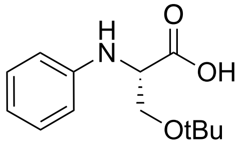

1o

***O*-(*tert*-butyl)-*N*-phenyl-L-serine (1o)**: Prepared from iodobenzene and *O*-(*tert*-butyl)-L-serine in 70% yield as an light yellow solid. **TLC**: R_f 0.34 (1% acetic acid in 3:2 EtOAc/Hexanes). **^{1}H NMR** (600 MHz, CD_2Cl_2) δ 7.25 – 7.18 (m, 2H), 6.85 – 6.79 (m, 1H), 6.72 – 6.65 (m, 2H), 4.07 (dd, *J* = 5.9, 4.2 Hz, 1H), 3.88 (dd, *J* = 9.0, 4.2 Hz, 1H), 3.63 (dd, *J* = 9.0, 5.9 Hz, 1H), 1.24 (s, 9H). **^{13}C NMR** (151 MHz, CD_2Cl_2) δ 173.0, 160.9, 146.8, 129.8, 119.8, 114.4, 75.2, 61.9, 58.1, 31.4, 28.5, 27.5. **ESI-MS**: Calcd for $C_{13}H_{20}NO_3$ ($[M+H]^+$) 238.1443; found 238.1447.

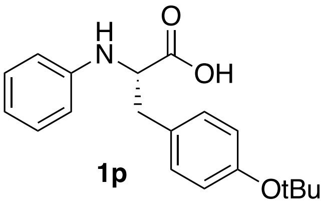

1p

(*S*)-3-(4-(*tert*-butoxy)phenyl)-2-(phenylamino)propanoic acid (1p): Prepared from iodobenzene and *O*-(*tert*-butyl)-L-tyrosine in 75% yield as an light yellow solid. **TLC**: R_f 0.32 (1% acetic acid in 2:3 EtOAc/Hexanes. **^{1}H NMR** (600 MHz, $CDCl_3$) δ 7.22 – 7.14 (m, 2H), 7.10 (d, *J* = 8.3 Hz, 2H), 6.98 – 6.89 (m, 2H), 6.79 (dd, *J* = 1497.9, 7.1 Hz, 1H), 6.59 (d, *J* = 7.9 Hz, 2H), 4.27 (t, *J* = 6.6 Hz, 1H), 3.23 (dd, *J* = 14.0, 5.3 Hz, 1H), 3.07 (dd, *J* = 14.1, 7.3 Hz, 1H), 1.33 (s, 9H). **^{13}C NMR** (151 MHz, $CDCl_3$) δ 176.5, 154.7, 146.2, 130.7, 129.9, 129.6, 124.6, 119.5, 114.2, 78.7, 58.4, 37.8, 28.9, 23.4, 20.7. **ESI-MS**: Calcd for $C_{19}H_{22}NO_3$ ($[M–H]^-$)312.1600; found 312.1595.

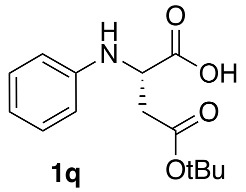

1q

(*S*)-4-(*tert*-butoxy)-4-oxo-2-(phenylamino)butanoic acid (1q): Prepared from iodobenzene and 4-*O*-(*tert*-butyl)-L-aspartic acid in 70% yield as a light yellow solid. **TLC**: R_f 0.20 (1% acetic acid in 2:3 EtOAc/Hexanes. **^{1}H NMR** (600 MHz, $CDCl_3$) δ 7.25 – 7.18 (m, 2H), 6.84 (d, *J* = 1.0 Hz, 1H), 6.73 – 6.66 (m, 2H), 4.40 – 4.29 (m, 1H), 2.95 – 2.86 (m, 1H), 2.82 (dd, *J* = 16.4, 5.4 Hz, 1H), 1.44 (s, 9H). **^{13}C NMR** (151 MHz, $CDCl_3$) δ 170.4, 145.86, 129.7, 119.8, 114.5, 114.5, 82.5, 54.3, 37.8, 28.3, 28.2. **ESI-MS**: Calcd for $C_{14}H_{19}NO_4$ ($[M+H]^+$) 288.1204; found 288.1204.

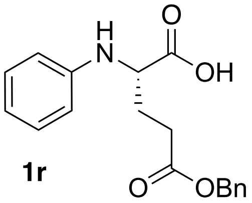

1r

(*S*)-5-(benzyloxy)-5-oxo-2-(phenylamino)pentanoic acid (1r): Prepared from iodobenzene and (*S*)-2-amino-5-(benzyloxy)-5-oxopentanoic acid in 20% yield as a white solid. **TLC**: R_f 0.18 (1% acetic acid in 2:3 EtOAc/Hexanes. **^{1}H NMR** (600 MHz, CD_2Cl_2) δ 7.40 – 7.26 (m, 5H), 7.21 – 7.10 (m, 2H), 6.79 – 6.73 (m, 1H), 6.61 (d, *J* = 7.7 Hz, 2H), 5.10 (s, 2H), 4.13 – 4.10 (m, 1H), 2.66 – 2.52 (m, 2H), 2.32 – 2.23 (m, 1H), 2.16 – 2.07 (m, 1H). **^{13}C NMR** (151 MHz, CD_2Cl_2) δ 177.8, 177.5, 173.2, 146.9, 136.4, 129.8, 128.9, 128.6, 128.6, 119.3, 119.3, 114.0, 114.0, 66.9, 56.6, 56.6, 30.9, 28.0, 28.0. **ESI-MS**: Calcd for $C_{18}H_{18}NO_4$ ($[M+H]^+$) 312.1236; found 312.1242.

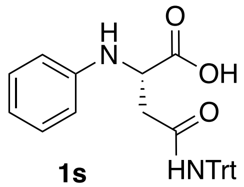

1s

N^2-phenyl-N^4-trityl-*L*-asparagine (1s): Prepared from iodobenzene and N^4-trityl-*L*-asparagine in 95% yield as a light yellow solid. **TLC**: R_f 0.35 (1% acetic acid in 2:3 EtOAc/Hexanes. **^{1}H NMR** (600 MHz, CD_3CN) δ 7.69 (s, 1H), 7.26 – 7.17 (m, 15H), 7.16 – 7.12 (m, 2H), 6.70 (tt, *J* = 7.3, 1.1 Hz, 1H), 6.65 – 6.61 (m, 2H), 5.42 (s, 1H), 4.32 (t, *J* = 6.2 Hz, 1H), 2.84 – 2.75 (m, 2H). **^{13}C NMR** (151 MHz, CD_3CN) δ 174.2, 170.7, 147.8, 145.5, 130.1, 129.6, 128.6, 127.7, 119.0, 114.40, 71.2, 55.3, 54.0, 39.7, 20.6. **ESI-MS**: Calcd for $C_{29}H_{26}N_2O_3Na$ ($[M+Na]^+$) 473.1841; found 473.1837.

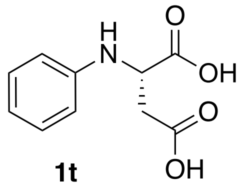

1t

***N*-Phenyl-L-aspartic acid (1t)**: Reaction solvent was changed to methanol. Prepared from iodobenzene and L-aspartic acid in 15% yield as an off-white solid. The NMR spectra of **1t** matched those reported in literature.[4]

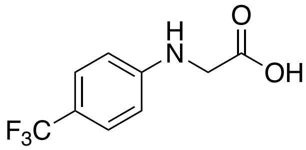

1x

***N*-Phenyl(4-trifluoromethane)-glycine (1x)**: Prepared from 4-trifluoromethane iodobenzene and glycine in 61% yield as a white solid. The NMR spectra of **1f** matched those reported in literature.[5]

D. CONVERSION OF *N*-ARYL α-AMINO ACIDS (1) TO 3-OXETANOLS (3)

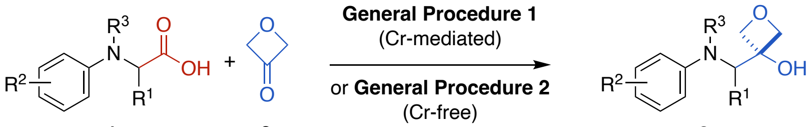

General Procedure 1 for Cr-mediated decarboxylative addition

In a 4-mL glass vial, 4CzIPN (1.58 mg, 2 μmol, 1 mol%), $CrCl_3$ (1.59 mg, 10 μmol, 5 mol%), cesium pivalate (56 mg, 0.24 mmol, 1.2 equiv), and the appropriate *N*-aryl α-amino acid **1** (0.2 mmol, 1 equiv) were combined. CH_3CN (0.25 mL) was added followed by 3-oxetanone (**2**) (12.8 μL, 0.2 mmol, 1 equiv). The mixture was degassed with Ar for 1 min, then TMSCl (12.5 μL, 0.1 mmol, 0.5 equiv) was added. The reaction was exposed to blue LED light (456 nm) at 25 °C for 20 h. The reaction mixture was filtered over celite and concentrated by rotary evaporation. Purification by silica flash chromatography (10–40% EtOAc in hexanes) yielded the 3-oxetanol product **3**.

General Procedure 2 for Cr-free decarboxylative addition

In a 4-mL glass vial, 4CzIPN (1.58 mg, 2 μmol, 1 mol%), cesium pivalate (56 mg, 0.24 mmol, 1.2 equiv), and the appropriate *N*-aryl α-amino acid **1** (0.2 mmol, 1 equiv) were combined. Methylene chloride (0.4 mL) was added followed by 3-oxetanone (**2**) (38.4 μL, 0.6 mmol, 3 equiv). The mixture was degassed with Ar for 1 min. The reaction was exposed to blue LED light (456 nm) at 25 °C for 2 h. The reaction mixture was filtered over celite and concentrated by rotary evaporation. Purification by silica flash chromatography (10–40% EtOAc in hexanes) yielded the 3-oxetanol product **3**.

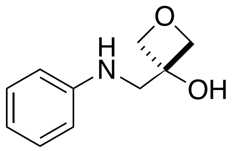

3a

3-((Phenylamino)methyl)oxetan-3-ol (3a): Prepared from **1a** by General Procedure 1 in 60% yield or by General Procedure 2 in 60% yield as a light brown solid. **TLC**: R_f 0.16 (2:3 EtOAc/Hexanes). **^{1}H NMR** (600 MHz) δ 7.22 (t, *J* = 7.7 Hz, 2H), 6.80 (t, *J* = 7.4 Hz, 1H), 6.73 (d, *J* = 7.5 Hz, 2H), 4.66 (d, *J* = 7.1 Hz, 2H), 4.58 (d, *J* = 7.4 Hz, 2H), 3.54 (s, 2H). **^{13}C NMR** (151 MHz, $CDCl_3$) δ 148.0, 129.6, 118.8, 113.7, 82.5, 73.7, 50.5. **ESI-MS**: Calcd for $C_{10}H_{13}NO_2$ ($[M+H]^+$) 180.1025; found 180.1019.

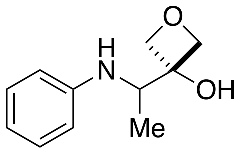

3b

3-(1-(Phenylamino)ethyl)oxetan-3-ol (3b): Prepared from **1b** by General Procedure 1 in 69% yield or by General Procedure 2 in 96% yield as a light brown solid. **TLC**: R_f 0.32 (2:3 EtOAc/Hexanes). **^{1}H NMR** (600 MHz, $CDCl_3$) δ 7.24 – 7.17 (m, 2H), 6.77 (t, 1H), 6.71 (d, 2H), 4.68 – 4.55 (m, 4H), 3.92 (q, J = 6.5 Hz, 1H), 1.24 (d, J = 6.5 Hz, 3H). **^{13}C NMR** (151 MHz, $CDCl_3$) δ 146.8, 129.6, 118.8, 114.4, 82.6, 81.6, 76.5, 54.0, 13.8. **ESI-MS**: Calcd for $C_{11}H_{15}NO_2$ ($[M+H]^+$) 194.1181; found 194.1184.

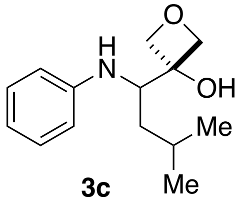

3c

3-(2-Phenyl-1-(phenylamino)ethyl)oxetan-3-ol (3c): Prepared from **1c** by General Procedure 1 in 67% yield or by General Procedure 2 in 72% yield as a white solid. **TLC**: R_f 0.42 (2:3 EtOAc/Hexanes). **^{1}H NMR** (600 MHz, CD_2Cl_2) δ 7.26 – 7.19 (m, 4H), 7.19 –7.15 (m, 1H), 7.14 – 7.08 (m, 2H), 6.68 – 6.59 (m, 3H), 4.55 (d, J = 7.5 Hz, 1H), 4.41 (d, J = 8.0 Hz, 1H), 4.19 (d, J = 7.7 Hz, 1H), 4.12 (d, J = 7.7 Hz, 1H), 4.08 (t, J = 6.8 Hz, 1H), 2.88 (d, J = 6.7 Hz, 2H). **^{13}C NMR** (151 MHz, CD_2Cl_2) δ 147.8, 138.9 129.7, 129.5, 128.9, 126.9, 118.1, 113.9, 83.6, 82.4, 77.4, 59.9, 36.1. **ESI-MS**: Calcd for $C_{17}H_{19}NO_2$ ($[M+H]^+$) 270.1494; found 270.1493.

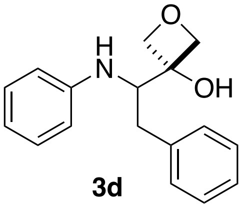

3d

3-(3-Methyl-1-(phenylamino)butyl)oxetan-3-ol (3d): Prepared from **1d** by General Procedure 1 in 52% yield or by General Procedure 2 in 99% yield as a white solid. **TLC**: R_f 0.35 (2:3 EtOAc/Hexanes). **^{1}H NMR** (600 MHz, $CDCl_3$) δ 7.21 – 7.12 (m, 2H), 6.75 – 6.64 (m, 3H), 4.65 – 4.59 (m, 2H), 4.56 – 4.51 (m, 2H), 3.86 (dd, J = 10.2, 3.1 Hz, 1H), 1.75 – 1.65 (m, 1H), 1.54 – 1.45 (m, 1H), 1.43 – 1.36 (m, 1H), 0.94 (d, J = 6.7 Hz, 3H), 0.91 (d, J = 6.5 Hz, 3H). **^{13}C NMR** (151 MHz, $CDCl_3$) δ 148.4, 129.6, 118.0, 113.3, 82.6, 81.7, 56.3, 40.2, 24.9, 23.9, 22.2. **ESI-MS**: Calcd for $C_{14}H_{21}NO_2$ ($[M+H]^+$) 236.1651; found 236.1645.

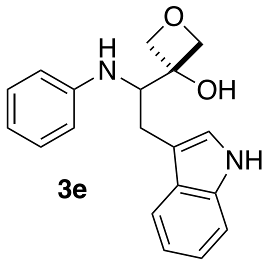

3e

3-(2-(1*H*-indol-3-yl)-1-(phenylamino)ethyl)oxetan-3-ol (3e): Prepared from **1e** by General Procedure 1 in 72% yield or by General Procedure 2 in 68% yield as a light brown solid. **TLC**: R_f 0.34 (3:7 EtOAc/Hexanes). **^{1}H NMR** (600 MHz, CD_2Cl_2) δ 8.00 (s, 1H–NH), 7.59 (d, J = 7.7 Hz, 1H), 7.35 (d, J = 8.3 Hz, 1H), 7.23 – 7.17 (m, 3H), 7.17 – 7.13 (m, 1H), 7.01 – 6.94 (m, 1H), 6.79 – 6.70 (m, 3H), 4.60 (d, J = 7.4 Hz, 1H), 4.47 (d, J = 7.4 Hz, 1H), 4.36 (q, J = 7.4 Hz, 2H), 4.27 (t, J = 6.3 Hz, 1H), 3.24 – 3.04 (m, 2H). **^{13}C NMR** (151 MHz, CD_2Cl_2) δ 136.4, 129.7, 127.3, 122.6, 120.0, 118.8, 111.8, 111.5, 83.3, 82.1, 24.9. **ESI-MS**: Calcd for $C_{19}H_{21}NO_2$ ($[M+H]^+$) 309.1603; found 309.1588.

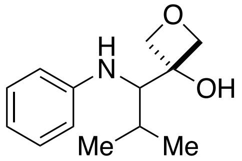

3f

3-(2-Methyl-1-(phenylamino)propyl)oxetan-3-ol (3f): Prepared from **1f** by General Procedure 1 in 28% yield or by General Procedure 2 in 89% yield as a white solid. **TLC**: R_f 0.28 (1:2 EtOAc/Hexanes). **^{1}H NMR** (600 MHz, $CDCl_3$) δ 7.18 – 7.13 (m, 2H), 6.71 – 6.65 (m, 3H), 4.80 (d, J = 7.4, 0.9 Hz, 1H), 4.68 (d, J = 7.6, 0.9 Hz, 1H), 4.53 (d, J = 7.4, 0.9 Hz, 1H), 4.43 (d, J = 7.5, 0.8 Hz, 1H), 3.62 (d, J = 7.1 Hz, 1H), 2.01 – 1.95 (m, 1H), 0.98 (d, J = 6.8 Hz, 3H), 0.93 (d, J = 6.8 Hz, 3H). **^{13}C NMR** (151 MHz, $CDCl_3$) δ 149.0, 129.8, 117.7, 113.2, 84.6, 83.3, 78.7, 63.2, 31.5, 21.0, 19.9. **ESI-MS**: Calcd for $C_{13}H_{19}NO_2$ ($[M+H]^+$) 222.1494; found 222.1485.

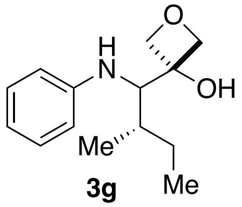

3g

3-(2-Methyl-1-(phenylamino)butyl)oxetan-3-ol (3g): Prepared from **1g** by General Procedure 1 in 36% yield or by General Procedure 2 in 78% yield as a brown oil. **TLC**: R_f 0.39 (2:3 EtOAc/Hexanes). **^{1}H NMR** (600 MHz, $CDCl_3$) δ 7.18 – 7.13 (m, 4H), 6.71 – 6.63 (m, 6H), 4.81 (d, J = 7.4, 0.9 Hz, 1H), 4.75 (d, J = 7.3, 0.8 Hz, 1H), 4.68 (d, J = 7.5, 1.1 Hz, 2H), 4.56 – 4.52 (m, 2H), 4.44 (t, J = 7.5, 6.5, 0.8 Hz, 2H), 3.77 (d, J = 5.1 Hz, 1H), 3.66 (d, J = 8.0 Hz, 1H), 1.79 – 1.73 (m, 1H), 1.73 – 1.63 (m, 2mH), 1.41 – 1.33 (m, 1H), 1.24 – 1.10 (m, 3H), 0.96 (d, J = 6.8 Hz, 4H), 0.94 – 0.84 (m, 11H). **^{13}C NMR** (151 MHz) δ 148.6, 129.6, 118.0, 113.5, 82.9, 81.9, 59.9, 23.8, 11.6. **ESI-MS**: Calcd for $C_{14}H_{21}NO_2$ ($[M+H]^+$) 236.1651; found 236.1653.

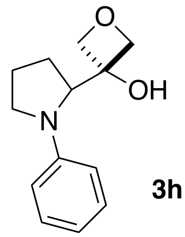

3h

3-(1-Phenylpyrrolidin-2-yl)oxetan-3-ol (3h): Prepared from **1h** by General Procedure 1 in 56% yield or by General Procedure 2 in 73% yield a yellow solid. **TLC**: R_f 0.40 (2:3 EtOAc/Hexanes). **^{1}H NMR** (600 MHz, CD_2Cl_2) δ 7.22 (t, J = 7.8 Hz, 2H), 6.79 – 6.71 (m, 3H), 4.81 (d, J = 6.9 Hz, 1H), 4.65 (d, J = 7.0 Hz, 1H), 4.43 (d, J = 7.0 Hz, 1H), 4.35 (d, J = 6.9 Hz, 1H), 4.12 – 4.05 (m, 1H), 3.71 – 3.64 (m, 1H), 3.28 – 3.19 (m, 1H), 2.07 – 1.99 (m, 2H), 1.99 – 1.92 (m, 1H), 1.91 – 1.81 (m, 1H). **^{13}C NMR** (151 MHz, CD_2Cl_2) δ 149.6, 129.5, 118.3, 114.6, 83.2, 81.3, 78.8, 63.0, 52.7, 27.9, 24.8. **ESI-MS**: Calcd for $C_{13}H_{17}NO_2$ ([M+H]$^+$) 220.1338; found 220.1333.

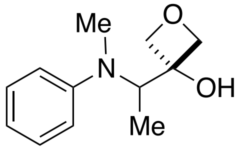

3i

3-(1-(Methyl(phenyl)amino)ethyl)oxetan-3-ol (3i): Prepared from **1i** by General Procedure 1 in 39% yield or by General Procedure 2 in 37% yield as a clear oil. **TLC**: R_f 0.31 (2:3 EtOAc/Hexanes). **^{1}H NMR** (600 MHz, CD_2Cl_2) δ 7.32 – 7.19 (m, 2H), 6.94 (d, J = 8.0 Hz, 2H), 6.89 – 6.80 (m, 1H), 4.79 (d, J = 6.7 Hz, 1H), 4.70 (d, J = 7.0 Hz, 1H), 4.59 (d, J = 6.7 Hz, 1H), 4.52 (d, J = 6.9 Hz, 1H), 3.89 (q, J = 6.8 Hz, 1H), 2.58 (s, 3H), 1.32 (d, J = 6.8 Hz, 3H). **^{13}C NMR** (151 MHz, CD_2Cl_2) δ 151.4, 129.46, 119.9, 116.8, 81.8, 81.4, 75.7, 61.0, 32.4, 9.2. **ESI-MS**: Calcd for $C_{12}H_{17}NO_2$ ([M+H]$^+$) 208.1338; found 208.1328.

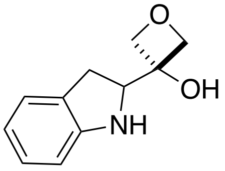

3j

3-(Indolin-2-yl)oxetan-3-ol (3j): Prepared from **1j** by General Procedure 1 in 43% yield or by General Procedure 2 in 42% yield as a light yellow solid. **TLC**: R_f 0.16 (2:3 EtOAc/Hexanes). **^{1}H NMR** (600 MHz, $CDCl_3$) δ 7.10 (d, J = 7.7 Hz, 1H), 7.05 (t, J = 7.7 Hz, 1H), 6.77 (t, J = 7.0 Hz, 1H), 6.67 (d, J = 7.8 Hz, 1H), 4.75 (d, J = 7.0 Hz, 1H), 4.68 (d, J = 6.5 Hz, 1H), 4.54 (d, J = 6.5, 1.3 Hz, 2H), 4.48 (t, J = 9.5 Hz, 1H), 3.27 – 3.18 (m, 1H), 3.06 – 2.98 (m, 1H). **^{13}C NMR** (151 MHz, $CDCl_3$) δ 150.4, 128.6, 127.7, 125.0, 120.1, 110.3, 83.0, 80.0, 74.4, 63.9, 30.7. **ESI-MS**: Calcd for $C_{11}H_{13}NO_2$ ([M+H]$^+$) 192.1025; found 192.1027.

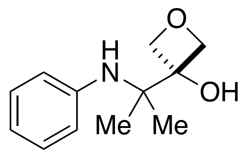

3k

3-(2-(Phenylamino)propan-2-yl)oxetan-3-ol (3k): Prepared from **1k** by General Procedure 1 in 22% yield or by General Procedure 2 in 29% yield as a clear oil. **TLC**: R_f 0.37 (2:3 EtOAc/Hexanes). **^{1}H NMR** (600 MHz, $CDCl_3$) δ 7.25 – 7.20 (m, 2H), 7.05 – 6.98 (m, 1H), 6.84 – 6.79 (m, 2H), 4.68 (d, J = 7.7 Hz, 2H), 4.63 (d, J = 7.7 Hz, 2H), 1.32 (s, 6H). **^{13}C NMR** (151 MHz, $CDCl_3$) δ 144.6, 129.1, 122.9, 80.7, 78.5, 22.7. **ESI-MS**: Calcd for $C_{12}H_{17}NO_2$ ($[M+H]^+$) 208.1338; found 208.1347.

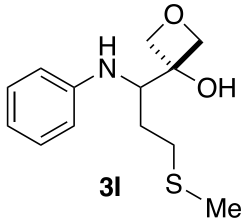

3l

3-(3-(Methylthio)-1-(phenylamino)propyl)oxetan-3-ol (3l): Prepared from **1l** by General Procedure 1 in 18% yield or by General Procedure 2 in 90% yield as a light yellow oil. **TLC**: R_f 0.21 (3:7 EtOAc/Hexanes). **^{1}H NMR** (600 MHz, $CDCl_3$) δ 7.20 – 7.13 (m, 2H), 6.75 – 6.66 (m, 3H), 4.64 (d, J = 7.1 Hz, 1H), 4.61 (d, J = 7.2 Hz, 1H), 4.56 (d, J = 7.1 Hz, 1H), 4.52 (d, J = 7.3 Hz, 1H), 4.07 (dd, J = 9.2, 3.6 Hz, 1H), 2.67 – 2.58 (m, 1H), 2.57 – 2.49 (m, 1H), 2.07 (s, 3H), 1.98 – 1.89 (m, 1H), 1.89 – 1.77 (m, 1H). **^{13}C NMR** (151 MHz, $CDCl_3$) δ 148.2, 129.7, 118.1, 113.4, 82.9, 82.0, 56.7, 31.2, 29.8, 15.7. **ESI-MS**: Calcd for $C_{13}H_{20}NO_2S$ ($[M+H]^+$) 254.1215; found 254.1207.

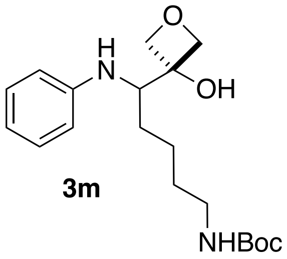

3m

***tert*-Butyl (5-(3-hydroxyoxetan-3-yl)-5-(phenylamino)pentyl)carbamate (3m):** Prepared from **1m** by General Procedure 1 in 77% yield or by General Procedure 2 in 83% yield as a light yellow solid. **TLC**: R_f 0.33 (3:7 EtOAc/Hexanes). **^{1}H NMR** (600 MHz, CD_2Cl_2) δ 7.20 – 7.08 (m, 2H), 6.73 – 6.57 (m, 3H), 4.60 (d, J = 7.0 Hz, 1H), 4.56 (d, J = 7.1 Hz, 1H), 4.50 – 4.44 (m, 2H), 3.79 (dd, J = 8.4, 5.1 Hz, 1H), 3.23 (s, 1H), 3.16 – 3.05 (m, 1H), 3.05 – 2.93 (m, 1H), 1.76 – 1.66 (m, 1H), 1.59 – 1.50 (m, 1H), 1.48 – 1.43 (m, 2H), 1.41 (s, 9H), 1.33 – 1.19 (m, 1H). **^{13}C NMR** (151 MHz, CD_2Cl_2) δ 156.7, 148.6, 129.8, 118.0, 113.7, 82.7, 82.0, 79.4, 77.4, 58.5, 39.9, 30.8, 30.1, 28.6, 23.6. **ESI-MS**: Calcd for $C_{19}H_{31}N_2O_4$ ($[M+H]^+$) 351.2284; found 351.2271.

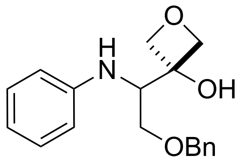

3n

3-(2-(Benzyloxy)-1-(phenylamino)ethyl)oxetan-3-ol (3n): Prepared from **1n** by General Procedure 1 in 37% yield or by General Procedure 2 in 40% yield as a brown oil. **TLC**: R_f 0.34 (3:7 EtOAc/Hexanes). **^{1}H NMR** (600 MHz, CD_2Cl_2) δ 7.42 – 7.21 (m, 5H), 7.21 – 7.13 (m, 2H), 6.75 – 6.63 (m, 3H), 4.61 – 4.47 (m, 6H), 4.05 – 4.01 (m, 1H), 3.95 – 3.89 (m, 1H, N–H), 3.81 (dd, *J* = 9.8, 2.8 Hz, 1H), 3.77 (dd, *J* = 9.8, 4.1 Hz, 1H), 1.21 (d, *J* = 6.5 Hz, 1H, O–H). **^{13}C NMR** (151 MHz, CD_2Cl_2) δ 147.4, 137.7, 129.8, 129.7, 128.9, 128.4, 128.1, 118.2, 114.0, 82.2 , 77.3, 74.1, 68.6, 56.3, 13.7. **ESI-MS**: Calcd for $C_{18}H_{22}NO_3$ ($[M+H]^+$) 300.1600; found 300.1603.

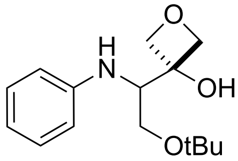

3o

3-(2-(*tert*-Butoxy)-1-(phenylamino)ethyl)oxetan-3-ol (3o): Prepared from **1o** by General Procedure 1 in 44% yield or by General Procedure 2 in 30% yield as a brown oil. **TLC**: R_f 0.40 (2:3 EtOAc/Hexanes). **^{1}H NMR** (600 MHz, $CDCl_3$) δ 7.19 (dd, *J* = 259.4, 7.6 Hz, 2H), 6.73 (d, *J* = 7.1 Hz, 1H), 6.66 (d, *J* = 8.3 Hz, 2H), 4.68 (d, *J* = 6.8 Hz, 1H), 4.62 – 4.58 (m, 2H), 4.58 – 4.55 (m, 2H), 4.31 (s, 1H, O–H), 4.01 (s, 1H, N–H), 3.77 – 3.68 (m, 2H), 1.13 (s, 9H).**^{13}C NMR** (151 MHz, $CDCl_3$) δ 146.8, 129.6, 118.1, 113.9, 82.3, 82.2, 74.9, 60.1, 55.7, 27.4. **ESI-MS**: Calcd for $C_{15}H_{24}NO_3$ ($[M+H]^+$) 266.1756; found 266.1762.

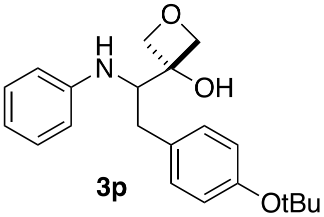

3p

3-(2-(4-(*tert*-Butoxy)phenyl)-1-(phenylamino)ethyl)oxetan-3-ol (3p): Prepared from **1p** by General Procedure 1 in 70% yield or by General Procedure 2 in 77% yield as a brown solid. **TLC**: R_f 0.18 (2:3 EtOAc/Hexanes). **^{1}H NMR** (600 MHz, $CDCl_3$) δ 7.20 – 7.13 (m, 2H), 7.13 – 7.09 (m, 2H), 6.93 – 6.85 (m, 2H), 6.74 – 6.66 (m, 1H), 6.66 – 6.60 (m, 2H), 4.62 (d, *J* = 7.5 Hz, 1H), 4.47 (d, *J* = 7.5 Hz, 1H), 4.22 (d, *J* = 7.7 Hz, 1H), 4.13 (d, *J* = 7.7 Hz, 1H), 4.10 – 4.04 (m, 1H), 2.95 – 2.82 (m, 2H), 1.30 (s, 9H). **^{13}C NMR** (151 MHz, $CDCl_3$) δ 154.1, 147.3, 133.3, 129.7, 129.6, 124.7, 118.1, 113.8, 83.9, 82.4, 78.6, 59.8, 35.3, 28.9. **ESI-MS**: Calcd for $C_{21}H_{28}NO_3$ ($[M+H]^+$) 342.2069; found 342.2068.

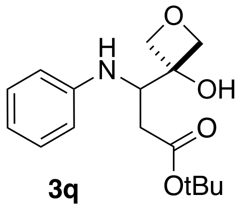

3q

***tert*-Butyl 3-(3-hydroxyoxetan-3-yl)-3-(phenylamino)propanoate (3q):** Prepared from **1q** by General Procedure 1 in 41% yield or by General Procedure 2 in 83% yield as a yellow solid. **TLC**: R_f 0.33 (3:7 EtOAc/Hexanes). **^{1}H NMR** (600 MHz, $CDCl_3$) δ 7.23 – 7.14 (m, 2H), 6.79 – 6.71 (m, 1H), 6.71 – 6.65 (m, 2H), 4.63 – 4.56 (m, 4H), 4.27 (d, J = 5.8 Hz, 1H), 2.72 (dd, J = 15.4, 6.1 Hz, 1H), 2.54 (dd, J = 15.4, 5.0 Hz, 1H), 1.36 (s, 9H). **^{13}C NMR** (151 MHz, $CDCl_3$) δ 172.4, 146.9, 129.6, 118.5, 113.8, 82.5, 82.2, 82.0, 76.7, 55.0, 36.0, 28.0. **ESI-MS**: Calcd for $C_{16}H_{24}NO_4$ ($[M+H]^+$) 294.1705; found 294.1705.

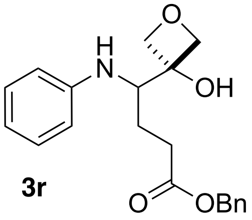

3r

Benzyl 4-(3-hydroxyoxetan-3-yl)-4-(phenylamino)butanoate (3r): Prepared from **1r** by General Procedure 1 in 35% yield or by General Procedure 2 in 49% yield as a light yellow oil. **TLC**: R_f 0.19 (2:3 EtOAc/Hexanes). **^{1}H NMR** (600 MHz, CD_2Cl_2) δ 7.43 – 7.22 (m, 5H), 7.19 – 7.05 (m, 2H), 6.76 – 6.54 (m, 3H), 5.07 (q, J = 12.5 Hz, 2H), 4.60 (d, J = 7.2 Hz, 1H), 4.53 (d, J = 7.3 Hz, 1H), 4.48 (d, J = 7.2 Hz, 1H), 4.45 (d, J = 7.1 Hz, 1H), 3.91 (dd, J = 9.4, 3.9 Hz, 1H), 2.56 – 2.39 (m, 2H), 2.05 – 1.92 (m, 1H), 1.88 – 1.77 (m, 1H). **^{13}C NMR** (151 MHz, CD_2Cl_2) δ 173.9, 148.4, 136.4, 129.8, 128.9, 128.7, 128.6, 118.2, 113.6, 82.7, 82.0, 77.3, 66.8, 57.7, 31.2, 25.3. **ESI-MS**: Calcd for $C_{20}H_{24}NO_4$ ($[M+H]^+$) 342.1705; found 342.1712.

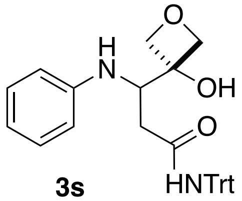

3s

3-(3-Hydroxyoxetan-3-yl)-3-(phenylamino)-*N*-tritylpropanamide (3s): Prepared from **1s** by General Procedure 1 in 37% yield or by General Procedure 2 in 76% yield as a white solid. **TLC**: R_f 0.27 (3:2 EtOAc/Hexanes). **^{1}H NMR** (600 MHz, CD_3CN) δ 7.34 (s, 1H, N–H), 7.08 – 6.87 (m, 17H), 6.56 (d, J = 8.0 Hz, 2H), 6.48 (t, J = 7.6 Hz, 1H), 4.63 (s, 1H, O–H), 4.33 (d, J = 9.7 Hz, 1H), 4.23 (d, J = 6.7 Hz, 1H), 4.19 (d, J = 6.6 Hz, 1H), 4.17 – 4.13 (m, 2H), 4.10 – 4.04 (m, 1H), 2.47 (dd, J = 15.1, 5.0 Hz, 1H), 2.30 (dd, J = 15.1, 6.5 Hz, 1H). **^{13}C NMR** (151 MHz, CD_3CN) δ 171.9, 148.9, 145.5, 130.2, 129.6, 128.6, 127.6, 114.0, 82.4, 82.2, 77.4, 71.0, 55.5, 55.5, 37.5. **ESI-MS**: Calcd for $C_{31}H_{31}N_2O_3$ ($[M+H]^+$) 479.12335; found 479.2352.

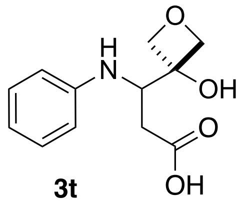

3t

3-(3-Hydroxyoxetan-3-yl)-3-(phenylamino)propanoic acid **(3t):** Prepared from **1t** by General Procedure 1 in 34% yield or by General Procedure 2 in 26% yield as an off-white solid. **TLC**: R_f 0.22 (3:2 EtOAc/Hexanes). **^{1}H NMR** (600 MHz, $CDCl_3$) δ 7.21 (t, J = 8.2 Hz, 2H), 6.78 (t, J = 7.7 Hz, 1H), 6.71 (d, J = 8.2 Hz, 2H), 4.66 (t, J = 9.1 Hz, 1H), 3.92 (q, J = 12.5 Hz, 2H), 3.76 (d, J = 12.4 Hz, 1H), 3.64 (d, J = 12.5 Hz, 1H), 3.05 (dd, J = 17.9, 9.2 Hz, 1H), 2.70 (dd, J = 17.9, 8.7 Hz, 1H). **^{13}C NMR** (151 MHz, $CDCl_3$) δ 175.1, 146.7, 129.8, 119.1, 113.7, 90.2, 63.8, 63.3, 52.0, 37.6. **ESI-MS**: Calcd for $C_{12}H_{16}NO_4$ ($[M+H]^+$) 238.1079; found 238.1076.

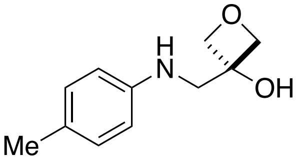

3u

3-((*p*-Tolylamino)methyl)oxetan-3-ol (3u): Prepared from **1u** by General Procedure 1 in 80% yield or by General Procedure 2 in 52% yield as a light brown oil. **TLC**: R_f 0.17 (2:3 EtOAc/Hexanes). **^{1}H NMR** (600 MHz, $CDCl_3$) δ 7.03 (d, J = 8.4 Hz, 2H), 6.66 (d, J = 8.4 Hz, 2H), 4.66 (d, J = 7.0 Hz, 2H), 4.57 (d, J = 8.0 Hz, 2H), 3.53 (s, 2H), 2.26 (s, 3H). **^{13}C NMR** (151 MHz, $CDCl_3$) δ 145.5, 130.1, 128.4, 114.1, 82.4, 73.6, 51.2, 20.6. **ESI-MS**: Calcd for $C_{11}H_{15}NO_2$ ($[M+H]^+$) 194.1181; found 194.1181.

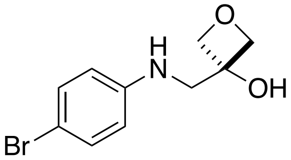

3v

3-(((4-Bromophenyl)amino)methyl)oxetan-3-ol (3v): Prepared from **1v** by General Procedure 1 in 47% yield or by General Procedure 2 in 64% yield as a light yellow solid. **TLC**: R_f 0.27 (2:3 EtOAc/Hexanes).^{1}H NMR (600 MHz, CD_2Cl_2) δ 7.33 – 7.21 (m, 2H), 6.64 – 6.55 (m, 2H), 4.59 (d, J = 7.3 Hz, 2H), 4.54 (d, J = 7.6 Hz, 2H), 3.48 (s, 2H). **^{13}C NMR** (151 MHz, CD_2Cl_2) δ 147.9, 132.3, 115.3, 109.8, 82.5, 74.0, 50.5. **ESI-MS**: Calcd for $C_{10}H_{13}NO_2Br$ ($[M+H]^+$) 258.0130; found 258.0132.

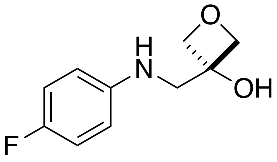

3-(((4-Fluorophenyl)amino)methyl)oxetan-3-ol (3w): Prepared from **1k** by General Procedure 1 in 53% yield or by General Procedure 2 in 75% yield as a clear oil. **TLC**: R_f 0.25 (2:3

EtOAc/Hexanes). **^{1}H NMR** (600 MHz, $CDCl_3$) δ 6.91 (t, *J* = 8.7 Hz, 2H), 6.69 – 6.61 (m, 2H), 4.65 (d, *J* = 7.0 Hz, 2H), 4.58 (d, *J* = 6.4 Hz, 2H), 3.49 (s, 2H). **^{13}C NMR** (151 MHz, $CDCl_3$) δ 157.4, 155.8, 144.3 (d, *J* = 2.0 Hz), 116.0 (d, *J* = 22.4 Hz), 114.8 (d, *J* = 7.5 Hz), 82.5, 73.7, 51.3. **ESI-MS**: Calcd for $C_{10}H_{12}FNO_2$ ($[M+H]^+$) 198.1930; found 198.0925.

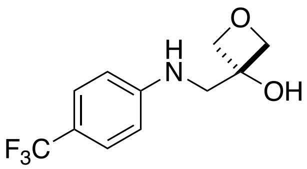

3-(((4-(Trifluoromethyl)phenyl)amino)methyl)oxetan-3-ol (3l): Prepared from **1l** by General Procedure 1 in 30% yield or by General Procedure 2 in 40% yield as a light brown oil. **TLC:** R_f 0.24 (1:1 EtOAc/Hexanes). **^{1}H NMR** (600 MHz, $CDCl_3$) δ 7.47 (d, *J* = 8.3 Hz, 2H), 6.84 (d, *J* = 8.4 Hz, 2H), 4.66 (d, *J* = 7.4 Hz, 2H), 4.58 (d, *J* = 7.6 Hz, 2H), 3.61 (s, 2H). **^{13}C NMR** (151 MHz, $CDCl_3$) δ 126.8, 113.6, 82.2, 50.4. **ESI-MS**: Calcd for $C_{11}H_{11}F_3NO_2$ ($[M–H]^-$) 246.0742; found 246.0734.

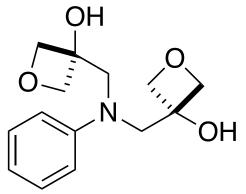

3y

3,3'-((Phenylazanediyl)bis(methylene))bis(oxetan-3-ol) (3y): Prepared from **1y** by General Procedure 1 in 10% yield as a clear oil. **TLC**: R_f 0.12 (1:1 EtOAc/Hexanes). **^{1}H NMR** (600 MHz, CD_2Cl_2) δ 7.34 – 7.26 (m, 2H), 7.20 – 7.11 (m, 2H), 7.00 (tt, *J* = 7.3, 1.1 Hz, 1H), 4.43 (d, *J* = 7.6 Hz, 4H), 4.36 (d, *J* = 7.7 Hz, 4H), 3.68 (s, 4H). **^{13}C NMR** (151 MHz, CD_2Cl_2) δ 151.2, 129.9, 122.9, 120.6, 82.9, 74.7, 61.7. **ESI-MS**: Calcd for $C_{14}H_{20}NO_4$ ($[M+H]^+$) 266.1392; found 266.1398.

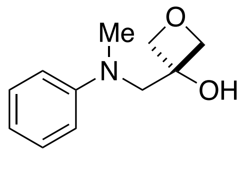

3z

3-((Methyl(phenyl)amino)methyl)oxetan-3-ol (3z): Prepared from **1y** by General Procedure 1 in 44% yield or by General Procedure 2 in 40% yield as a clear oil. **TLC**: R_f 0.28 (2:3 EtOAc/Hexanes). **^{1}H NMR** (600 MHz, CD_2Cl_2) δ 7.29 – 7.21 (m, 2H), 6.91 (d, *J* = 8.1 Hz, 2H), 6.82 (t, *J* = 7.3 Hz, 1H), 4.66 – 4.57 (m, 4H), 3.62 (s, 2H), 2.88 (s, 3H). **^{13}C NMR** (151 MHz, CD_2Cl_2) δ 151.6, 129.5, 119.2, 115.1, 83.2, 74.2, 60.5, 39.9. **ESI-MS**: Calcd for $C_{11}H_{15}NO_2$ ($[M+H]^+$) 194.1181; found 194.1183.

E. Conversion of *N,N*-Dialkyl-α-Amino Acids (4) to 3-Oxetanols (5)

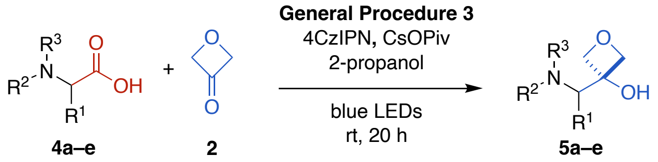

General Procedure 3 for decarboxylative coupling of N-aliphatic α-amino acids

In a 4-mL glass vial, 4CzIPN (3.155 mg, 4 μmol, 2 mol%), cesium pivalate (56 mg, 0.24 mmol, 1.2 equiv), and the appropriate α-amino acid **5** (0.2 mmol, 1 equiv) were combined. 2-Propanol (0.5 mL) was added followed by 3-oxetanone (**2**) (12.8 μL, 0.2 mmol, 1 equiv). The mixture was degassed with Ar for 1 min. The reaction was exposed to blue LED light (456 nm) at 25 °C for 20 h. The reaction mixture was filtered over celite and concentrated by rotary evaporation. Purification by silica flash chromatography yielded the 3-oxetanol product **5**.

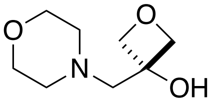

5a

3-(morpholinomethyl)oxetan-3-ol (5a): Prepared from **4a** by General Procedure 3 (modification of 3 equiv 3-oxetanone used) in 30% yield as a light brown oil. **TLC**: R_f 0.16 (1:4 Methanol/DCM). **^{1}H NMR** (600 MHz, $CDCl_3$) δ 4.74 (d, J = 6.8 Hz, 2H), 4.49 (d, J = 7.3 Hz, 2H), 3.81 – 3.71 (m, 4H), 2.90 (s, 2H), 2.58 (s, 4H). **^{13}C NMR** (151 MHz, $CDCl_3$) δ 83.61, 71.00, 66.46, 64.31, 53.53. **ESI-MS**: Calcd for $C_8H_{16}NO_3$ ($[M+H]^+$) 174.1130; found 174.1130.

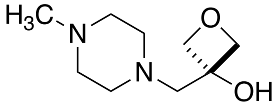

5b

3-((4-methylpiperazin-1-yl)methyl)oxetan-3-ol (5b): Prepared from **4b** by General Procedure 3 in 32% yield as a light brown oil. **TLC**: R_f 0.17 (1:4 Methanol/DCM). **^{1}H NMR** (500 MHz, $CDCl_3$) δ 4.73 (d, J = 6.7 Hz, 2H), 4.49 (d, J = 6.7 Hz, 2H), 2.81 (s, 2H), 2.61 – 2.37 (m, 8H), 2.31 (s, 3H). **^{13}C NMR** (151 MHz, $CDCl_3$) δ 83.92, 71.00, 63.89, 54.92, 52.85, 45.96. **ESI-MS**: Calcd for $C_9H_{19}N_2O_2$ ($[M+H]^+$) 187.1447; found 187.1445.

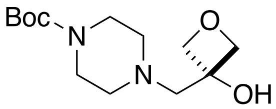

5c

***tert*-butyl 4-((3-hydroxyoxetan-3-yl)methyl)piperazine-1-carboxylate (5c):** Prepared from **4c** by General Procedure 3 in 21% yield as a light brown oil. **TLC**: R_f 0.17 (3:2 EtOAc/Hexanes with 1% triethyl amine). **^{1}H NMR** (600 MHz, $CDCl_3$) δ 4.67 (d, J = 7.3 Hz, 2H), 4.43 (d, J = 7.4 Hz,

2H), 3.37 (t, *J* = 4.8 Hz, 4H), 2.74 (s, 2H), 2.34 (d, *J* = 5.0 Hz, 4H), 1.39 (s, 9H). **^{13}C NMR** (151 MHz, $CDCl_3$) δ 154.70, 83.87, 80.12, 71.04, 64.09, 52.91, 28.53. **ESI-MS**: Calcd for $C_{13}H_{25}N_2O_4$ ($[M+H]^+$) 273.1826; found 273.1814.

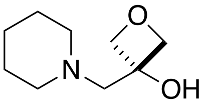

5d

3-(piperidin-1-ylmethyl)oxetan-3-ol (5d): Prepared from **4d** by General Procedure 3 in 29% yield as a light brown oil. **TLC**: R_f 0.14 (1:4 Methanol/DCM). **^{1}H NMR** (600 MHz, CD_2Cl_2) δ 4.62 (d, *J* = 6.0 Hz, 2H), 4.45 (d, *J* = 1.0 Hz, 2H), 2.89 (s, 2H), 2.68 – 2.34 (m, 4H), 1.65 (p, *J* = 5.7 Hz, 4H), 1.55 – 1.40 (m, 2H). **^{13}C NMR** (151 MHz, CD_2Cl_2) δ 83.83, 71.30, 64.39, 54.83, 25.28, 23.70. **ESI-MS**: Calcd for $C_9H_{18}NO_2$ ($[M+H]^+$) 172.1338; found 172.1333.

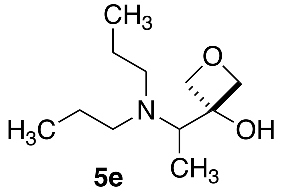

5e

3-(1-(dipropylamino)ethyl)oxetan-3-ol (5e): Prepared from **4e** by General Procedure 3 in 40% yield as a clear oil. **TLC**: R_f 0.14 (1:4 Methanol/DCM). **^{1}H NMR** (500 MHz, CD_2Cl_2) δ 4.72 (d, *J* = 6.6 Hz, 1H), 4.67 (d, *J* = 7.0 Hz, 1H), 4.50 (d, *J* = 6.6 Hz, 1H), 4.42 (d, *J* = 6.9 Hz, 1H), 2.81 (q, *J* = 6.9 Hz, 1H), 2.40 – 2.30 (m, 2H), 2.27 – 2.15 (m, 2H), 1.47 (dtt, *J* = 15.5, 8.0, 2.3 Hz, 2H), 1.43 – 1.35 (m, 2H), 1.28 (d, *J* = 6.9 Hz, 3H), 0.86 (t, *J* = 7.4 Hz, 6H). **^{13}C NMR** (126 MHz, CD_2Cl_2) δ 82.26, 81.01, 74.24, 59.67, 52.39, 21.65, 11.90, 6.69. **ESI-MS**: Calcd for $C_{11}H_{24}NO_2$ ($[M+H]^+$) 202.1807; found 202.1808.

F. Mechanistic Studies

1. Deuterium quenching and competition experiments

General Procedure 1 (Section D) was followed for entries 1–3 with the minor modifications of using deuterium exchanged *N*-phenyl glycine with CH_3OD (entries 2,3), excluding 3-oxetanone and adding 327 µL of CH_3OD (entry 2), or adding 327 µL CH_3OD (entry 3). General Procedure 2 (Section D) was followed for entries 4–6 with the minor modifications of using deuterium exchanged *N*-phenyl glycine with CH_3OD (entries 2,3), excluding 3-oxetanone and adding 327 µL of CH_3OD (entry 2), or adding 327 µL CH_3OD (entry 3). Mesitylene (27.8 µL, 0.2 mmol, 0.5 equiv) was added as an internal standard for ^{1}H-NMR analysis.

The following control experiment was also carried out to exclude formation of **4b** by H/D-exchange between **4a** and CH_3OD in the presence of CsOPiv.

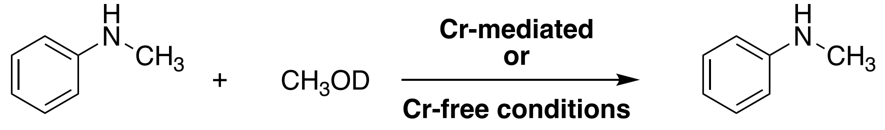

13a
1 equiv

20 equiv

13a
(0% D-incorporation **13b**)

2. DIFFERENTIAL PULSE VOLTAMMETRY (DPV) MEASUREMENTS OF 3-OXETANONE

DPV measurements were performed using a 660E potentiostat/galvanostat model from CH Instruments. A standard three-electrode configuration was used for these experiments, which were a glassy carbon working electrode, a platinum wire counter electrode, and a Ag/AgCl reference electrode. The supporting electrolyte used was tetrabutylammonium tetrafluoroborate ($TBAPF_6$) dissolved in acetonitrile (0.1 M) and analyte concentration was 20 mM. Standard reduction potentials were obtained against Ag/AgCl and converted against standard calomel electrode (SCE). The 3-oxetanone wave was confirmed using Fc/Fc^+ as an internal standard.

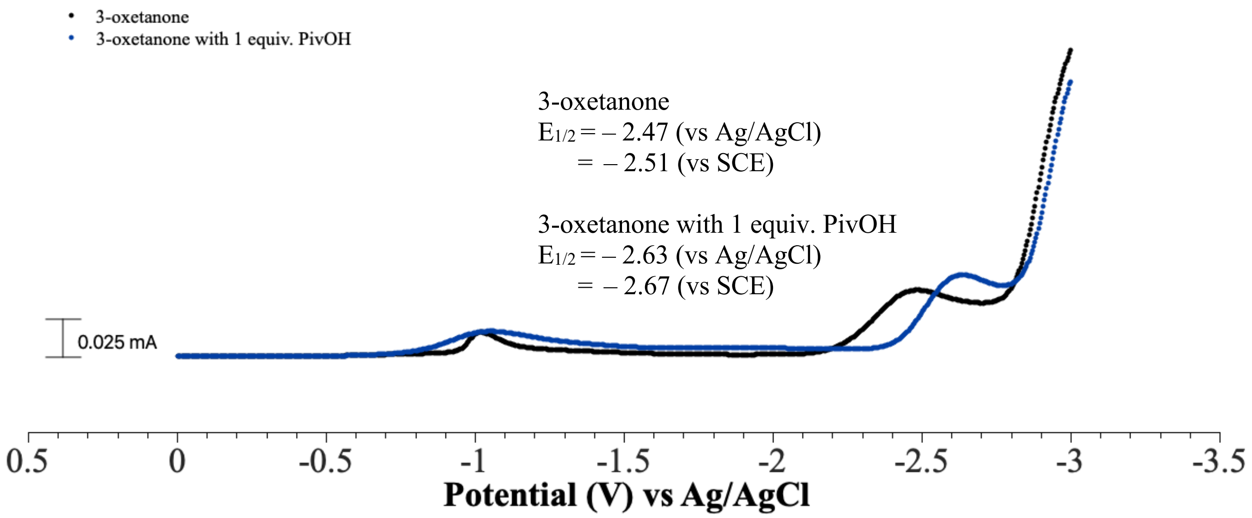

Supplementary Figure S3. Differential pulse voltammetry of 3-oxetanone and 3-oxetanone with 1 equiv. pivalic acid.

3. Stern–Volmer Fluorescence Quenching Analysis

Photocatalyst fluorescence quenching measurements were obtained on a fluorimeter (Fluoromax-4, Horiba Jobin-Yvon). For emission quenching experiments, a 45 µM solution of 4CzIPN in acetonitrile was added to 3.5 mL quartz cuvettes (10 mm) with varying concentrations of quencher added. The cuvettes were equipped PTFE/Septa screw caps and degassed by argon sparging. The samples were irradiated at 435 nm and fluorescence emission was measured at 535 nm. A Stern–Volmer plot was created by plotting the ratio of emission intensity (I_0/ I) in the absence and presence of individual quenchers at different concentrations.

I_0 = emission intensity of photocatalyst in the absence of quencher at a given wavelength
I = observed emission intensity of photocatalyst with quencher

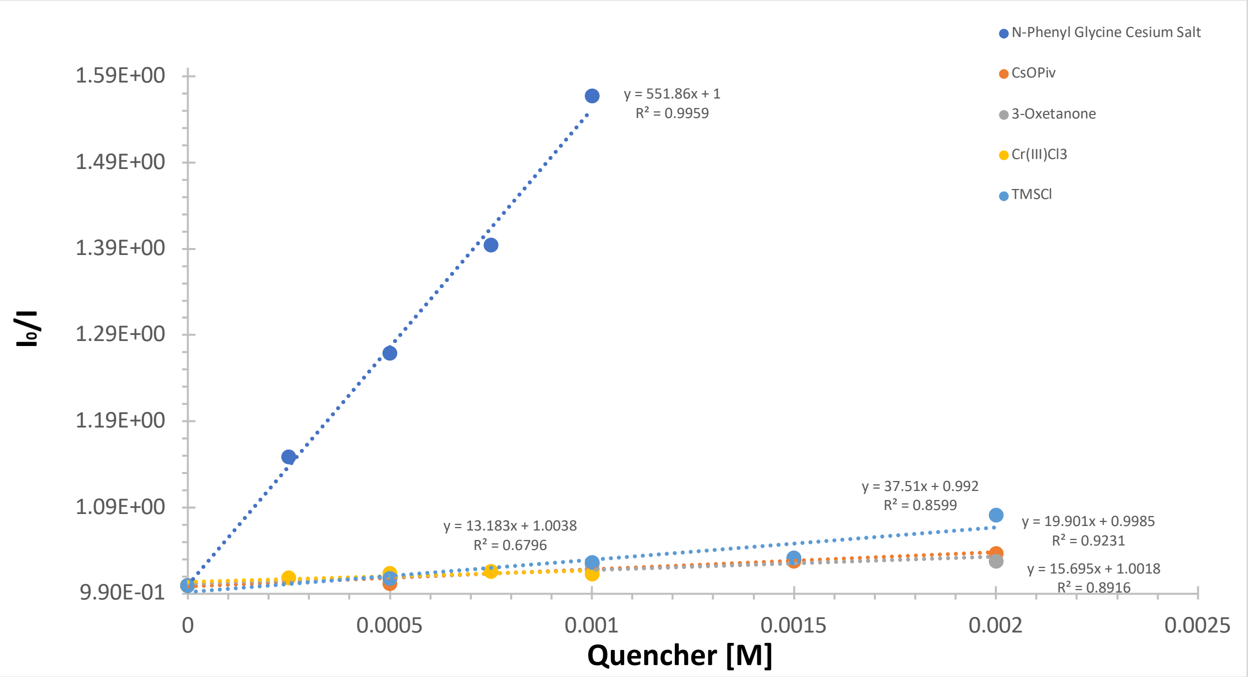

Supplementary Figure S4. Stern–Volmer plot of 4CzIPN (45 µM) in acetonitrile with varying concentrations of different quenchers.

This data indicates that *N*-phenyl glycine (cesium salt), cesium pivalate, 3-oxetanone, and Cr(III)Cl_3, and TMSCl quench the excited state photocatalyst, however, the Stern–Volmer constant of *N*-phenyl glycine (cesium salt) is much larger than that of the other reagents.

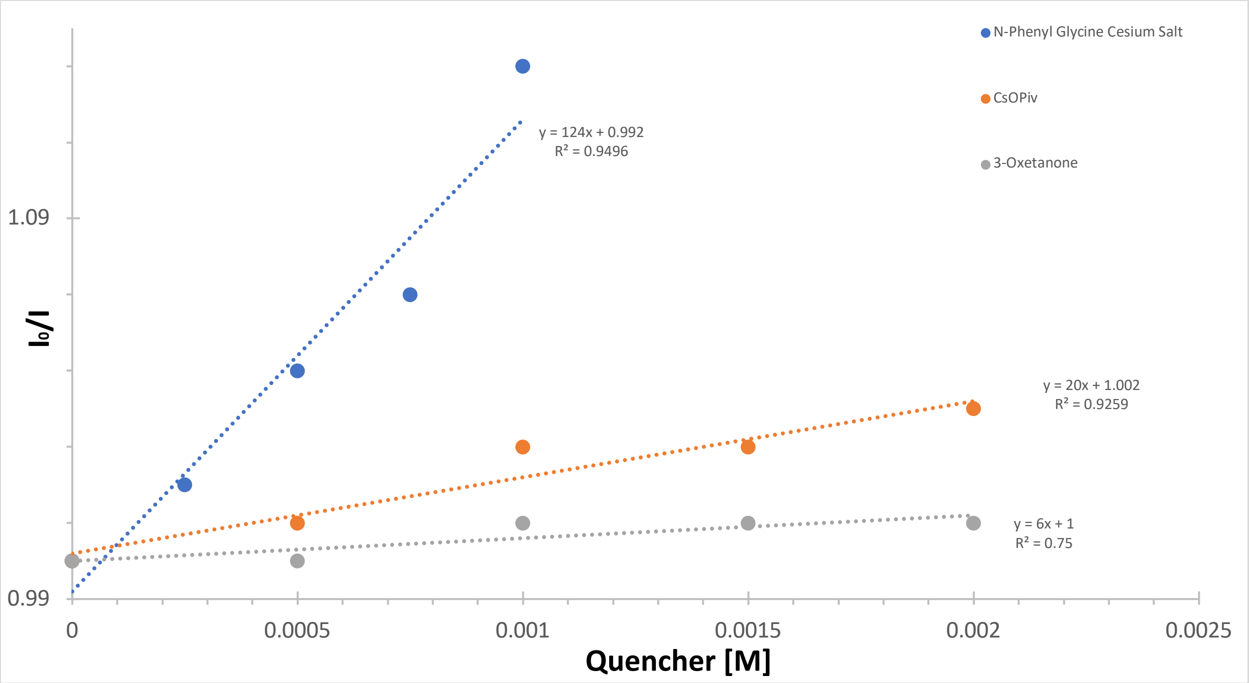

Supplementary Figure S5. Stern–Volmer plot of 4CzIPN (45 μM) in methylene chloride with varying concentrations of different quenchers.

This data indicates that *N*-phenyl glycine (cesium salt), cesium pivalate, and 3-oxetanone quench the excited state photocatalyst, however, the Stern–Volmer constant of *N*-phenyl glycine (cesium salt) is much larger than that of the other reagents.

4. QUANTUM YIELD DETERMINATION

The quantum yield of the reaction was determined using a published protocol.[6]

Determination of photon flux

The photon flux of the LED (Kessil PR160L, 40 W, λ_{max} = 456 nm) was measured via standard ferrioxalate actinometry.[7,8] A solution of ferrioxalate (0.15 M) was prepared by dissolving 2.21 g of potassium ferrioxalate hydrate in 30 mL of 0.05 M H_2SO_4. A buffered solution of 1,10-phenanthroline was prepared by dissolving 25 mg of phenanthroline and 5.63 g of sodium acetate in 25 mL of 0.5 M H_2SO_4. Solutions were stored in the dark. 3 mL of the ferrioxalate solution was added to 4 mL vials and irradiated for 60 seconds. After irradiation, 0.525 mL of the phenanthroline solution was added and the sample was allowed to rest for 1 hour for coordination. Next, the mixture was transferred to a quartz cuvette and the absorbance was measured at 510 nm. Non-irradiated samples as controls were also prepared. Photoconversion of Fe^{3+} to Fe^{2+} was calculated using eq 1.

1.

$$mol\ Fe^{2+} = \frac{\mathrm{V} * \Delta\mathrm{A}}{l * \varepsilon}$$

V is the total volume (0.003525 L), ΔA is the difference in absorbance at 510 nm between the irradiated and non-irradiated samples, l is the path length (1 cm), and ε is the molar absorptivity at 510 nm (11,100 L mol^{-1} cm^{-1}). After the $mol\ Fe^{2+}$ was calculated from the equation 1, the photon flux was determined using eq 2.

2.

$$photon\ flux = \frac{mol\ Fe^{2+}}{\Phi * t * f}$$

Φ is the quantum yield for the ferrioxalate actinometer (0.846 for 0.15 M at λ = 457.9 nm),[9] t is the time (60 seconds), and f is the fraction of light absorbed by the ferrioxalate actinometer at λ = 456 nm (0.964). The fraction of light absorbed is calculated using eq 3.

3.

$$f = 1 - 10^{-A\ (at\ 456\ nm)}$$

The average photon flux from 3 experiments was determined to be 1.79 x 10^{-8} einsteins per second.

Determination of quantum yield

Conditions 1 (Cr-mediated)

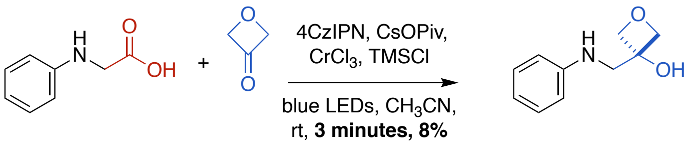

In a 4-mL glass vial, 4CzIPN (1.58 mg, 2 μmol, 1 mol%), $CrCl_3$ (1.59 mg, 10 μmol, 5 mol%), cesium pivalate (56 mg, 0.24 mmol, 1.2 equiv), and the appropriate *N*-aryl α-amino acid **1** (0.2 mmol, 1 equiv) were combined. CH_3CN (0.25 mL) was added followed by 3-oxetanone (**2**) (12.8 μL, 0.2 mmol, 1 equiv). The mixture was degassed with Ar for 1 min, then TMSCl (12.5 μL, 0.1 mmol, 0.5 equiv) was added. The reaction was exposed to blue LED light (456 nm) at 25 °C for 3 minutes. After irradiation, the yield was determined using ^{1}H-NMR analysis of crude reaction product in the presence of mesitylene as an internal standard, relative to limiting reagent 3-oxetanone. The yield obtained was 8% (1.6 x 10^{-5} mol of product). The quantum yield of the reaction was calculated using eq 4, where photon flux is 1.79 x 10^{-8} einsteins per second, t is time (180 seconds), and f is the fraction of light absorbed by the reaction mixture in the conditions described (0.95).

4.

$$quantum\ yield = \frac{mol\ \text{product}}{photon\ flux * t\ * f}$$

The quantum yield (Φ) was determined to be 5.2.

Conditions 1 (Cr-mediated) in the absence of TMSCl

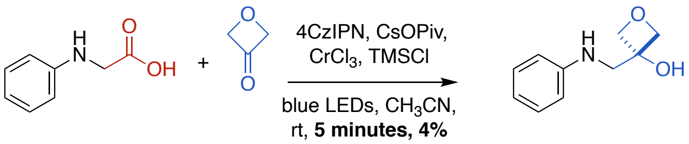

In a 4-mL glass vial, 4CzIPN (1.58 mg, 2 μmol, 1 mol%), $CrCl_3$ (1.59 mg, 10 μmol, 5 mol%), cesium pivalate (56 mg, 0.24 mmol, 1.2 equiv), and the appropriate *N*-aryl α-amino acid **1** (0.2 mmol, 1 equiv) were combined. CH_3CN (0.25 mL) was added followed by 3-oxetanone (**2**) (12.8 μL, 0.2 mmol, 1 equiv). The mixture was degassed with Ar for 1 min. The reaction was exposed to blue LED light (456 nm) at 25 °C for 5 minutes. After irradiation, the yield was determined using ^{1}H-NMR analysis of crude reaction product in the presence of mesitylene as an internal standard, relative to limiting reagent 3-oxetanone. The yield obtained was 4% (0.8 x 10^{-5} mol of product). The quantum yield of the reaction was calculated using eq 4, where photon flux is 1.79 x 10^{-8} einsteins per second, t is time (300 seconds), and f is the fraction of light absorbed by the reaction mixture in the conditions described (0.95).

The quantum yield (Φ) was determined to be 1.6.

<u>*Conditions 2 (Cr-free)*</u>

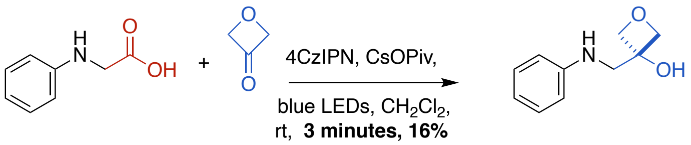

In a 4-mL glass vial, 4CzIPN (1.58 mg, 2 μmol, 1 mol%), cesium pivalate (56 mg, 0.24 mmol, 1.2 equiv), and the appropriate *N*-aryl α-amino acid **1** (0.2 mmol, 1 equiv) were combined. Methylene chloride (0.4 mL) was added followed by 3-oxetanone (**2**) (38.4 μL, 0.6 mmol, 3 equiv). The mixture was degassed with Ar for 1 min. The reaction was exposed to blue LED light (456 nm) at 25 °C for 3 minutes. After irradiation, the yield was determined using ^{1}H-NMR analysis of crude reaction product in the presence of mesitylene as an internal standard, relative to limiting reagent 3-oxetanone. The yield obtained was 16% (3.2×10^{-5} mol of product). The quantum yield of the reaction was calculated using eq 4, where photon flux is 1.79×10^{-8} einsteins per second, t is time (180 seconds), and f is the fraction of light absorbed by the reaction mixture in the conditions described (0.96).

The quantum yield (Φ) was determined to be 10.3.

G. SUPPORTING INFORMATION REFERENCES

1. A. B. Pangborn, M. A. Giardello, R. H. Grubbs, R. K. Rosen and F. J. Timmers, *Organometallics* **1996**, *15*, 1518–1520.
2. K. K. Sharma, S. Sharma, A. Kudwal and R. Jain, *Org. Biomol. Chem.* **2015**, *13*, 4637–4641.
3. X. Hu, X. Chen, B. Li, G. He and G. Chen, *Org. Lett.* **2021**, *23*, 716–721.
4. H. Fu, A. Prats Luján, L. Bothof, J. Zhang, P. G. Tepper and G. J. Poelarends, *ACS Catalysis* **2019**, *9*, 7292–7299.
5. S. Specklin, E. Decuypere, L. Plougastel, S. Aliani and F. Taran, *J. Org. Chem.* **2014**, *79*, 7772–7777.
6. M. A. Cismesia and T. P. Yoon, *Chem. Sci.* **2015**, *6*, 5426–5434.
7. C. G. Hatchard and C. A. Parker, *Proc. R. Soc. Lond.* **1956**, *A235*, 518–536.
8. M. Montalti, A. Credi, L. Prodi and M. T. Gandolfi, *Handbook of Photochemistry*, Taylor & Francis Group, LLC, 3rd edn., 2006.
9. J. N. Demas, W. D. Bowman, E. F. Zalewski and R. A. Velapoldi, *J. Phys. Chem.* **1981**, *85*, 2766–2771.

H. ^{1}H-NMR AND ^{13}C-NMR SPECTRA

1. SYNTHESIS OF *N*-ARYL-α-AMINO ACIDS (1n–s) S35

2. CONVERSION OF *N*-ARYL-α-AMINO ACIDS (1) TO 3-OXETANOLS (3) S41

3. CONVERSION OF *N*,*N*-DIALKYL-α-AMINO ACIDS (4) TO 3-OXETANOLS (5) S68

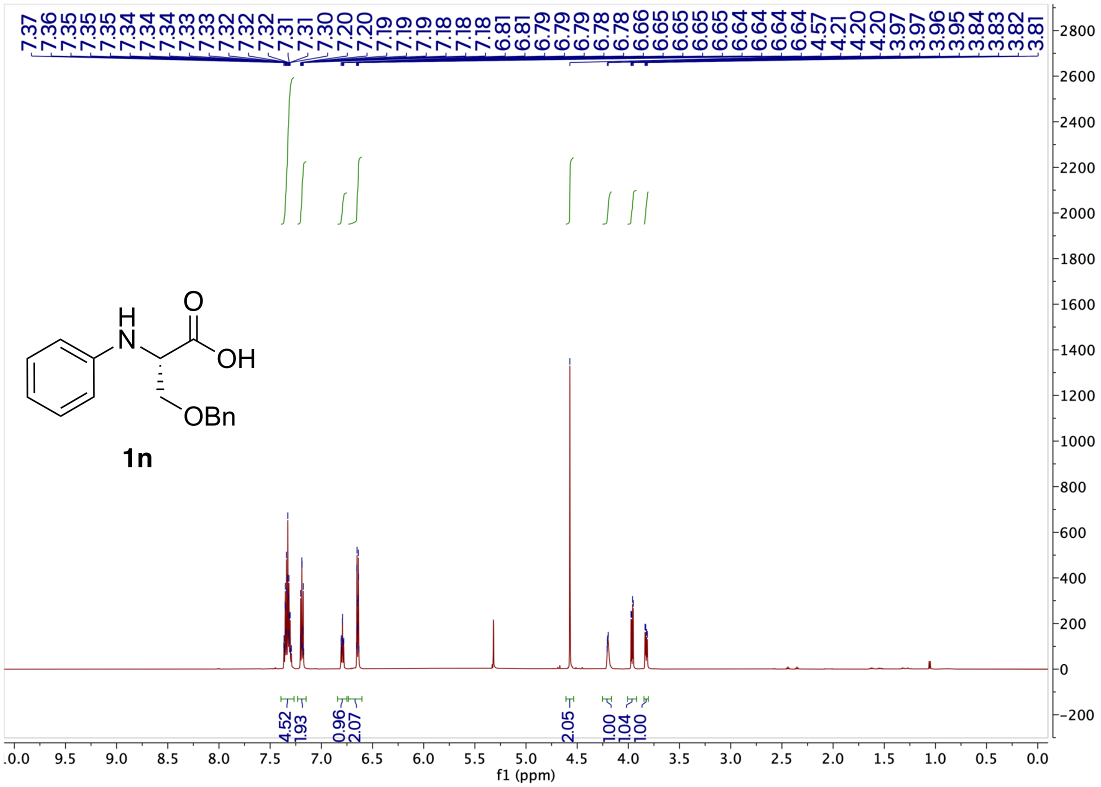
7.37 7.36 7.35 7.35 7.35 7.34 7.34 7.33 7.33 7.32 7.32 7.32 7.31 7.31 7.30 7.20 7.20 7.19 7.19 7.19 7.18 7.18 7.18 6.81 6.81 6.79 6.79 6.79 6.78 6.78 6.66 6.65 6.65 6.65 6.64 6.64 6.64 6.64 4.57 4.21 4.20 3.97 3.97 3.96 3.95 3.84 3.83 3.82 3.81
H
N
O
OH
OBn
1n
4.52
1.93
0.96
2.07
2.05
1.00
1.04
1.00
f1 (ppm)

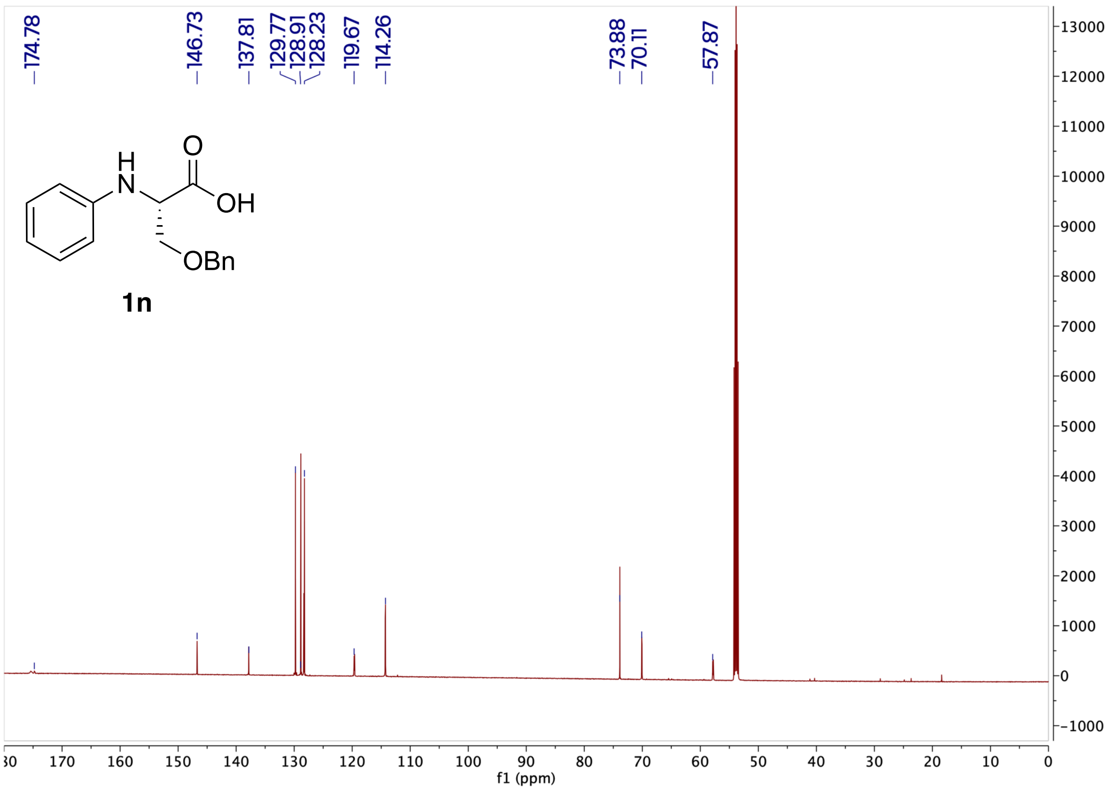
174.78
146.73
137.81
129.77
128.91
128.23
119.67
114.26
73.88
70.11
57.87
H
N
O
OH
OBn
1n
f1 (ppm)

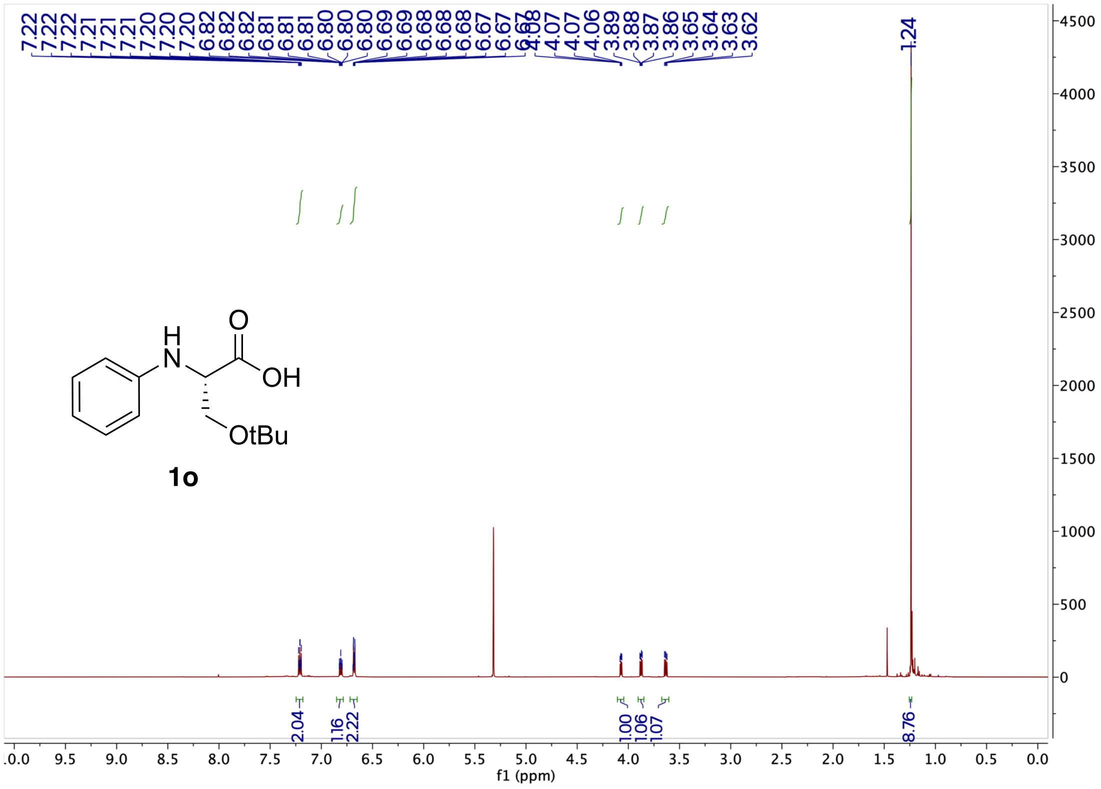
7.22
7.22
7.22
7.21
7.21
7.21
7.20
7.20
7.20
6.82
6.82
6.82
6.81
6.81
6.81
6.80
6.80
6.80
6.69
6.69
6.68
6.68
6.68
6.67
6.67
6.67
4.08
4.07
4.07
4.06
3.89
3.88
3.87
3.86
3.65
3.64
3.63
3.62
1.24
2.04
1.16
2.22
1.00
1.06
1.07
8.76
f1 (ppm)
OH
OtBu
1o

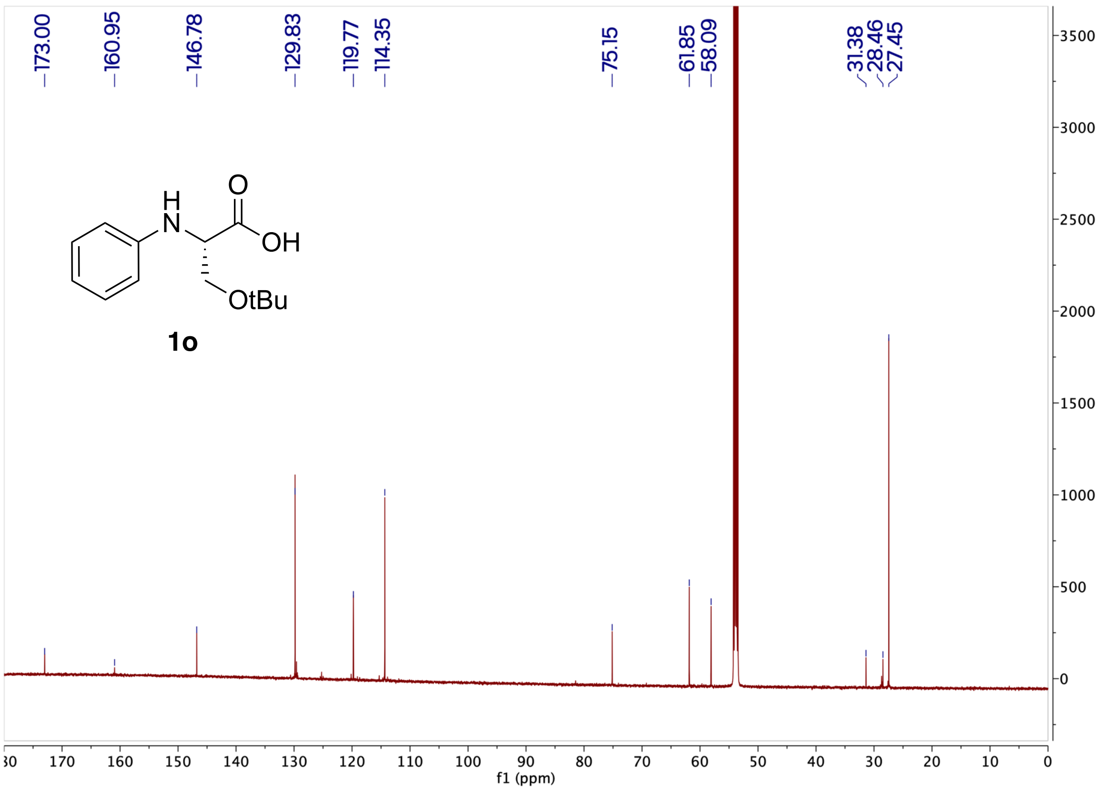
173.00
160.95
146.78
129.83
119.77
114.35
75.15
61.85
58.09
31.38
28.46
27.45
f1 (ppm)
OH
OtBu
1o

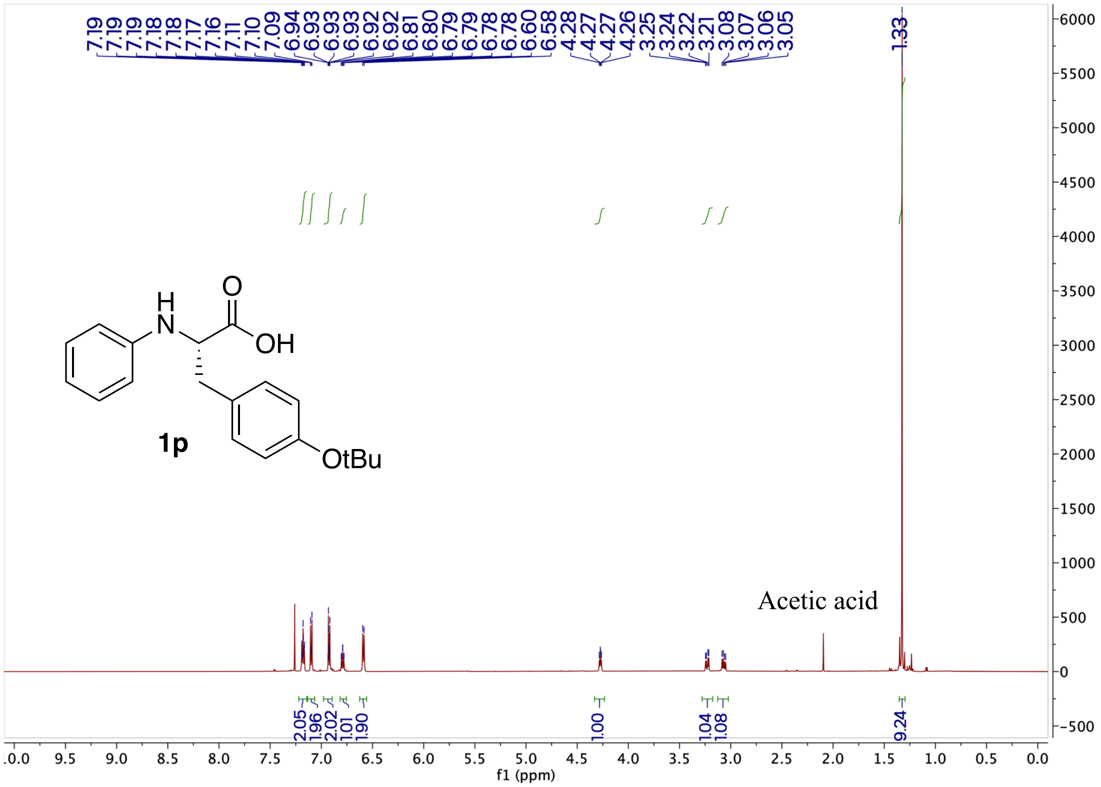
7.19
7.19
7.19
7.18
7.17
7.16
7.11
7.10
7.09
6.94
6.93
6.93
6.93
6.92
6.92
6.81
6.80
6.79
6.78
6.78
6.60
6.58
4.28
4.27
4.27
4.26
3.25
3.24
3.22
3.21
3.08
3.07
3.06
3.05
1.33
H
N
O
OH
1p
OtBu
Acetic acid
2.05
1.96
2.02
1.01
1.90
1.00
1.04
1.08
9.24
f1 (ppm)

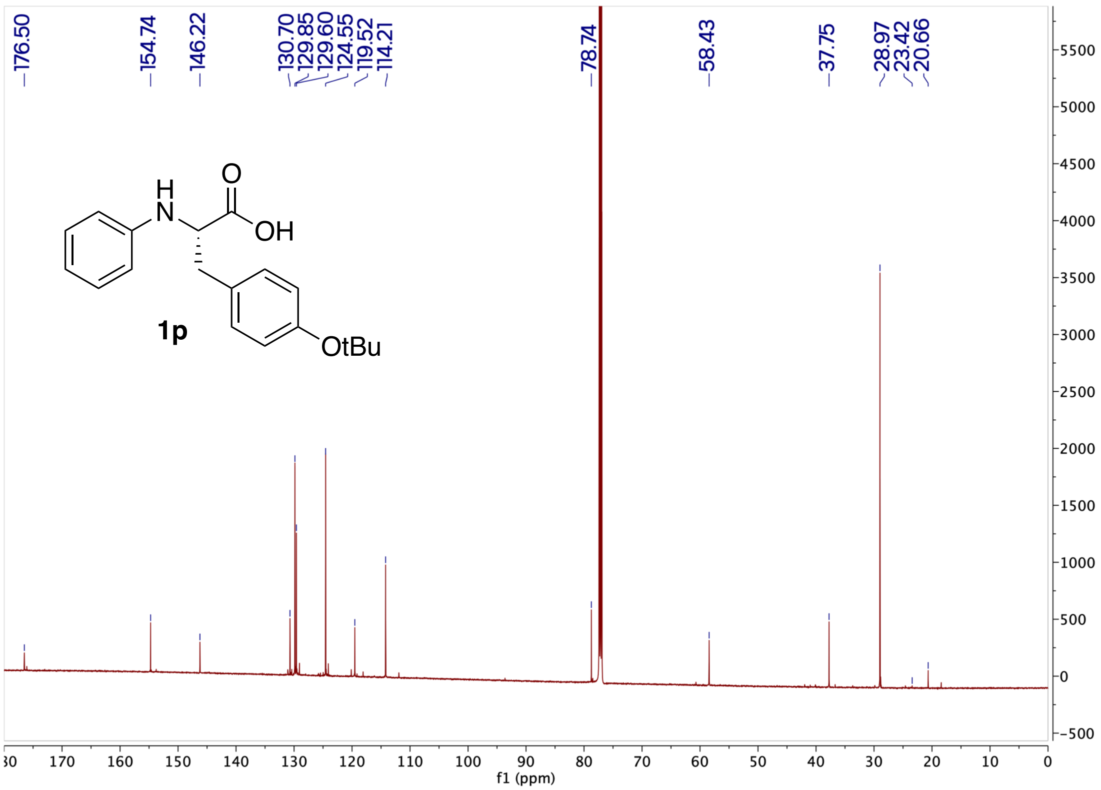
176.50
154.74
146.22
130.70
129.85
129.60
124.55
119.52
114.21
78.74
58.43
37.75
28.97
23.42
20.66
H
N
O
OH
1p
OtBu
f1 (ppm)

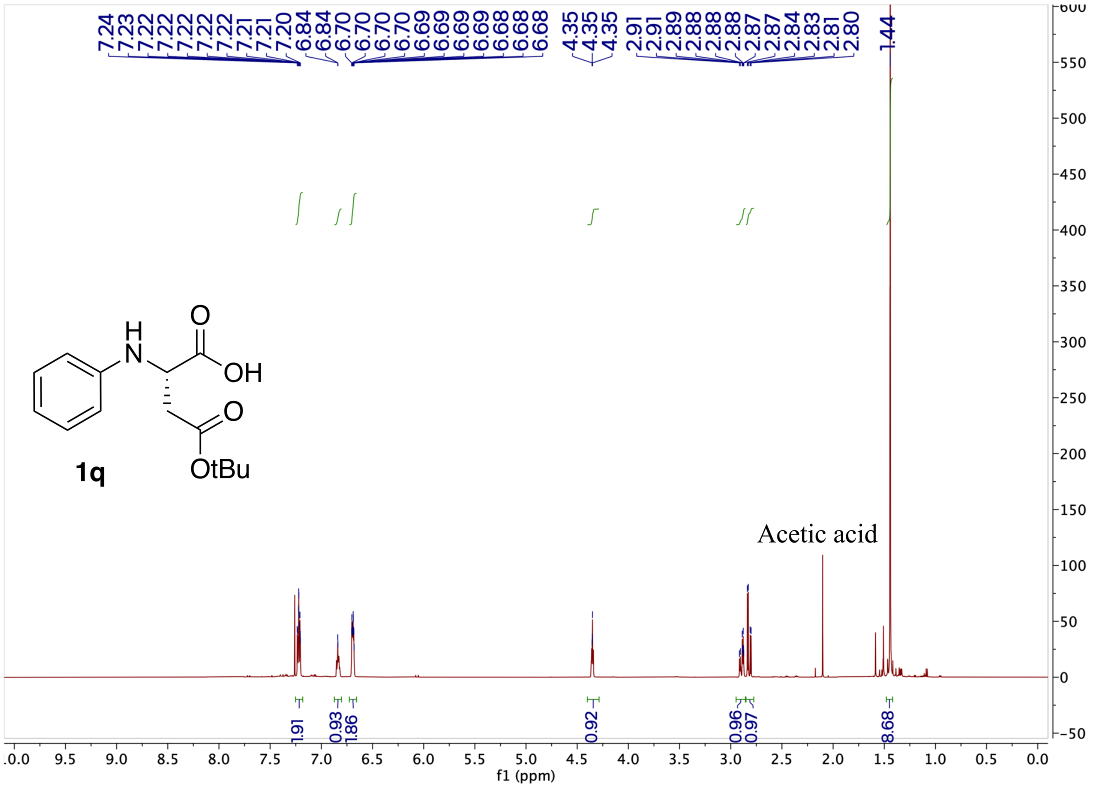
7.24 7.23 7.22 7.22 7.22 7.22 7.22 7.21 7.21 7.20 6.84 6.84 6.70 6.70 6.70 6.69 6.69 6.69 6.68 6.68 6.68
4.35 4.35 4.35
2.91 2.91 2.89 2.88 2.88 2.88 2.87 2.87 2.84 2.83 2.81 2.80
1.44
1q
OtBu
OH
Acetic acid
1.91 0.93 1.86 0.92 0.96 0.97 8.68
f1 (ppm)

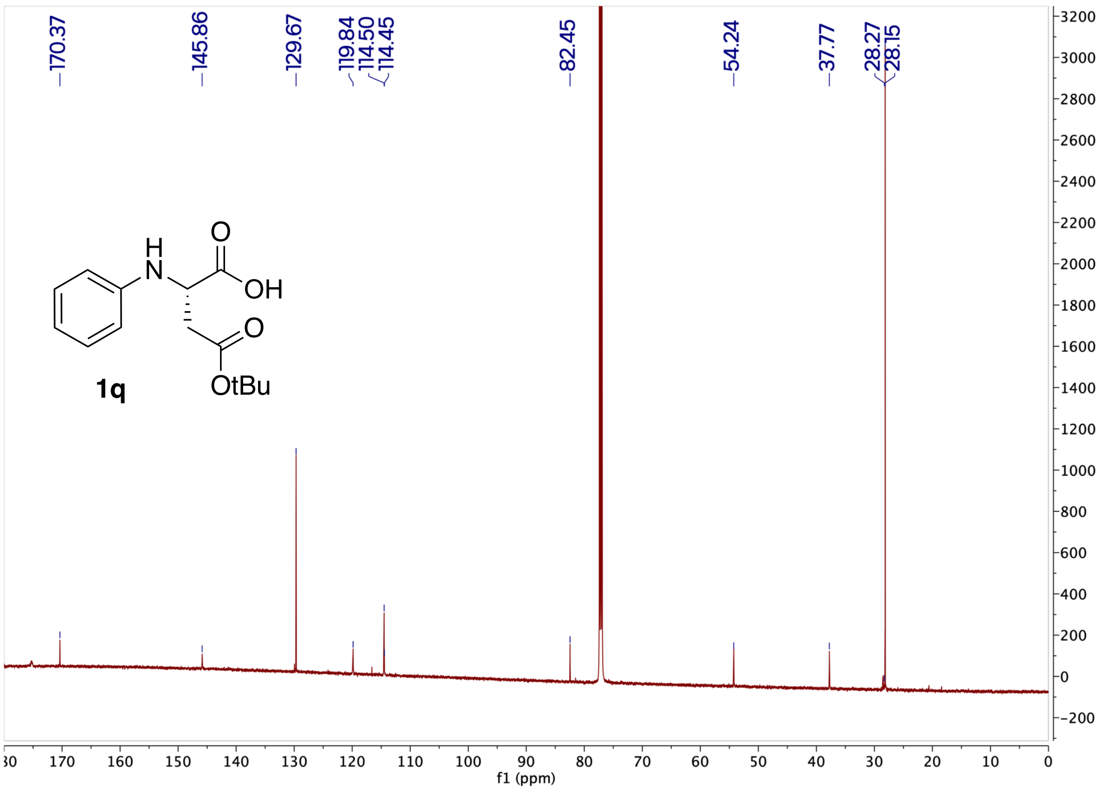
170.37
145.86
129.67
119.84 114.50 114.45
82.45
54.24
37.77
28.27 28.15
1q
OtBu
OH
f1 (ppm)

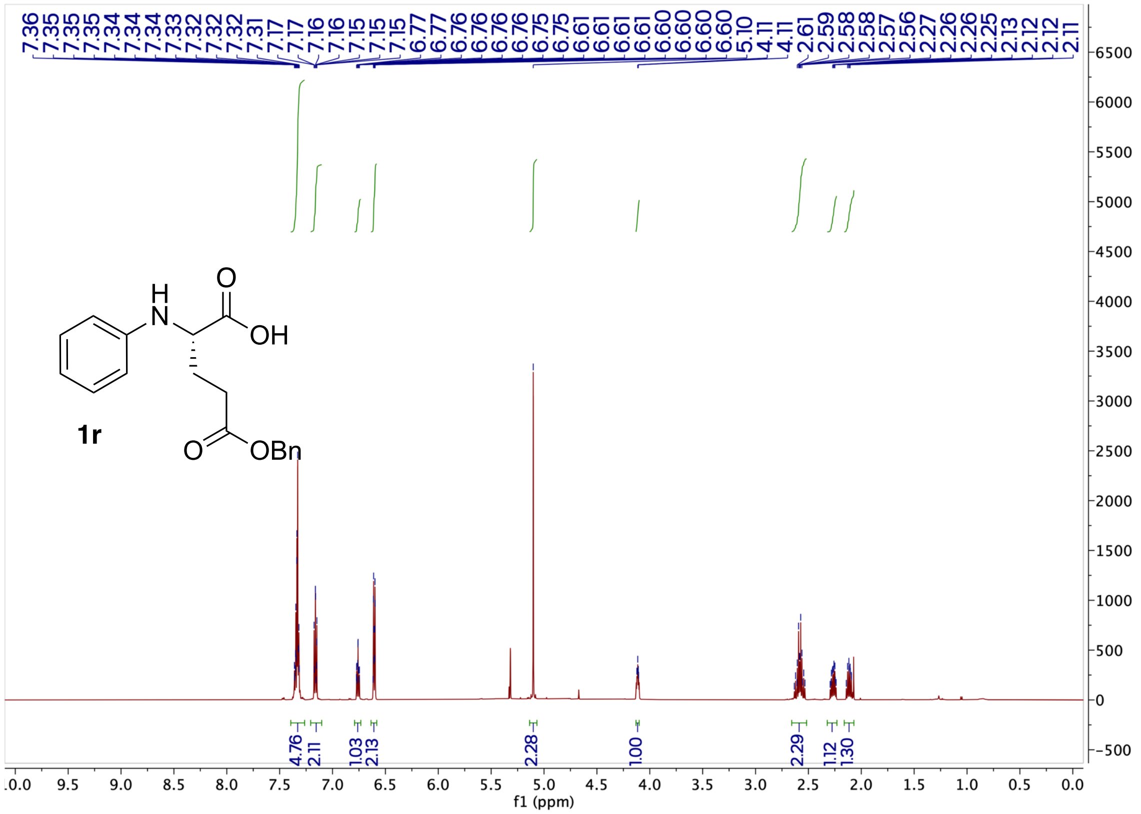
7.36 7.35 7.35 7.35 7.34 7.34 7.34 7.33 7.32 7.32 7.32 7.31 7.17 7.17 7.16 7.16 7.15 7.15 7.15 6.77 6.76 6.76 6.76 6.76 6.75 6.75 6.61 6.61 6.61 6.61 6.60 6.60 6.60 6.60 5.10 4.11 4.11 2.61 2.59 2.58 2.57 2.56 2.27 2.26 2.25 2.13 2.12 2.12 2.11
H
N
O
OH
1r
O
OBn
4.76 2.11 1.03 2.13 2.28 1.00 2.29 1.12 1.30
f1 (ppm)

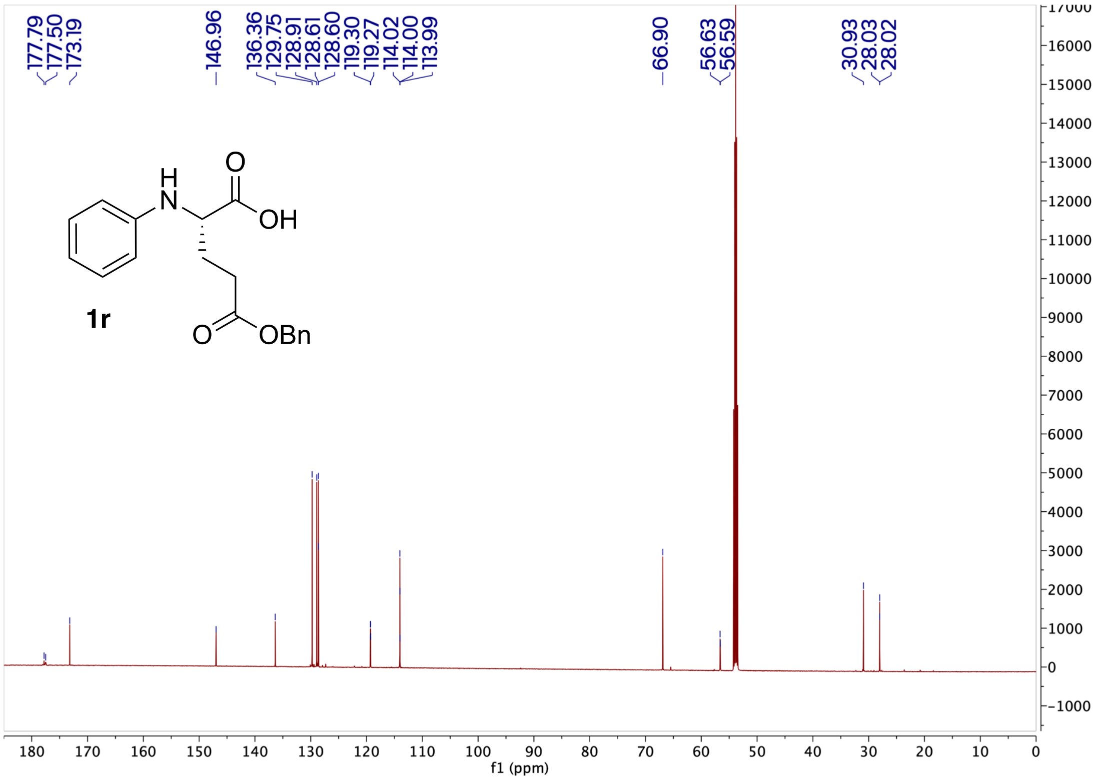
177.79 177.50 173.19 146.96 136.36 129.75 128.91 128.61 128.60 119.30 119.27 114.02 114.00 113.99 66.90 56.63 56.59 30.93 28.03 28.02
H
N
O
OH
1r
O
OBn
f1 (ppm)

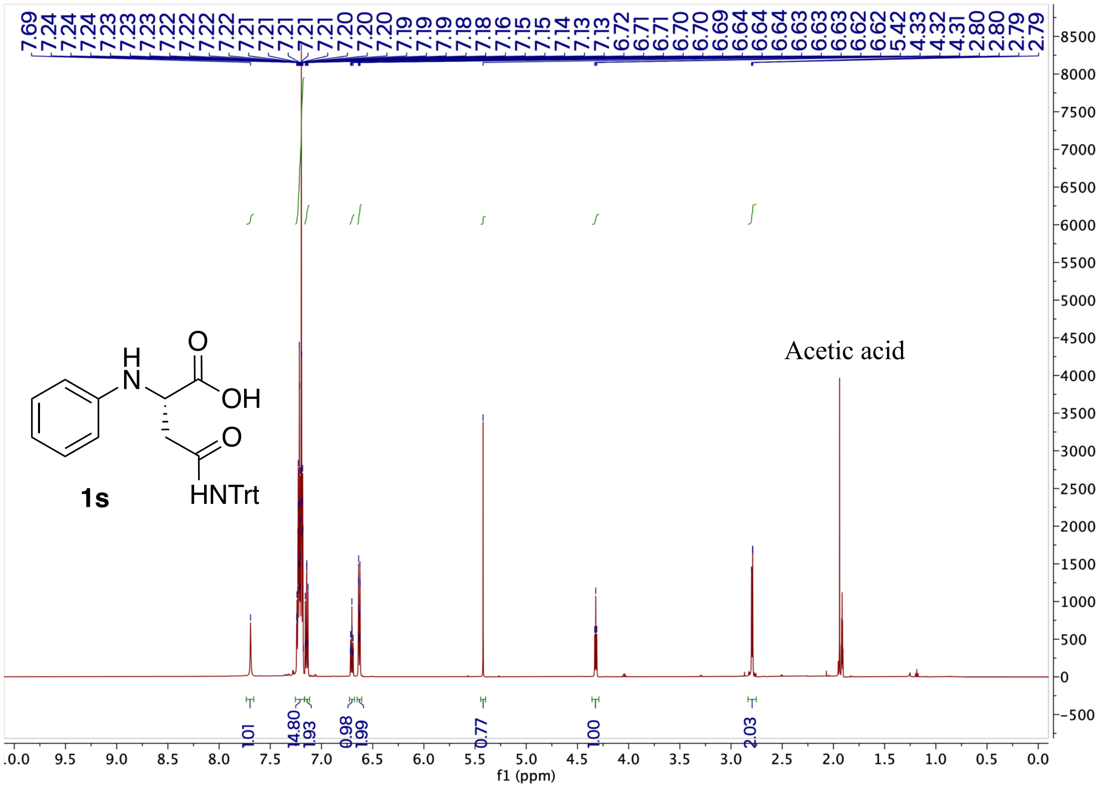
7.69
7.24
7.24
7.24
7.23
7.23
7.23
7.22
7.22
7.22
7.22
7.21
7.21
7.21
7.21
7.21
7.20
7.20
7.19
7.19
7.19
7.18
7.16
7.15
7.15
7.14
7.13
7.13
6.72
6.71
6.71
6.70
6.70
6.69
6.64
6.64
6.63
6.63
6.63
6.62
6.62
5.42
4.33
4.32
4.31
2.80
2.80
2.79
2.79
Acetic acid
1s
HNTrt
OH
1.01
14.80
1.93
0.98
1.99
0.77
1.00
2.03
f1 (ppm)

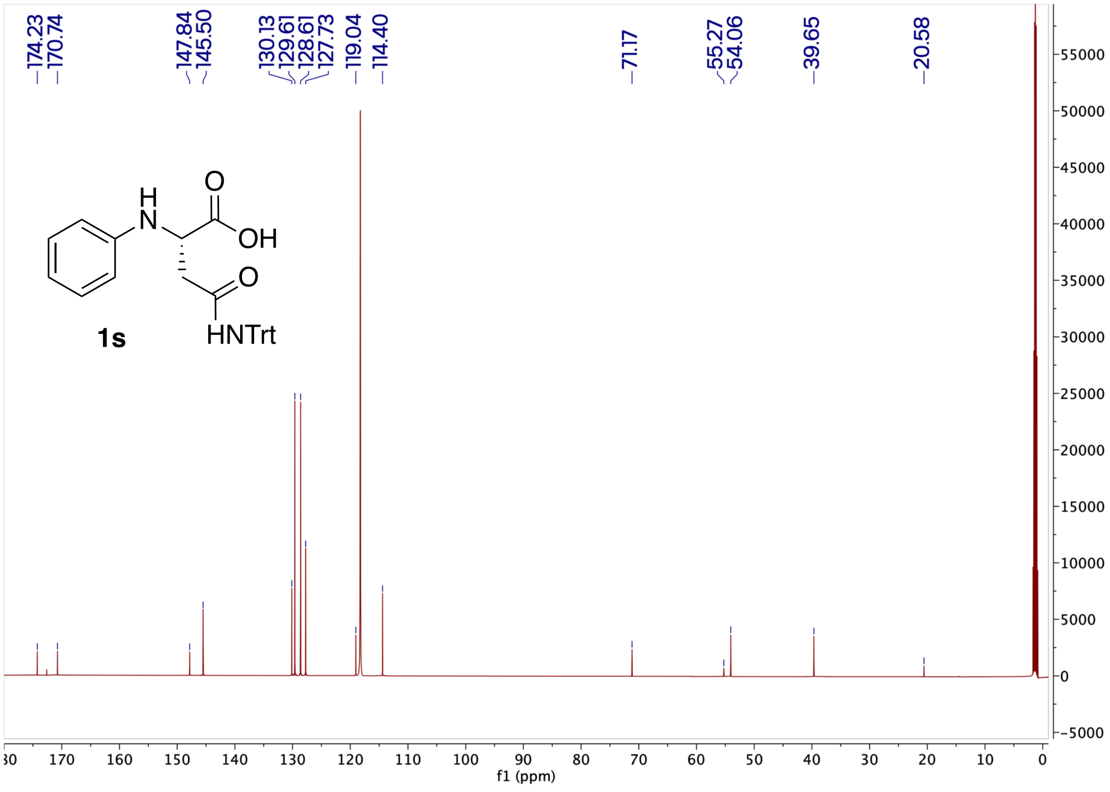
174.23
170.74
147.84
145.50
130.13
129.61
128.61
127.73
119.04
114.40
71.17
55.27
54.06
39.65
20.58
1s
HNTrt
OH
f1 (ppm)

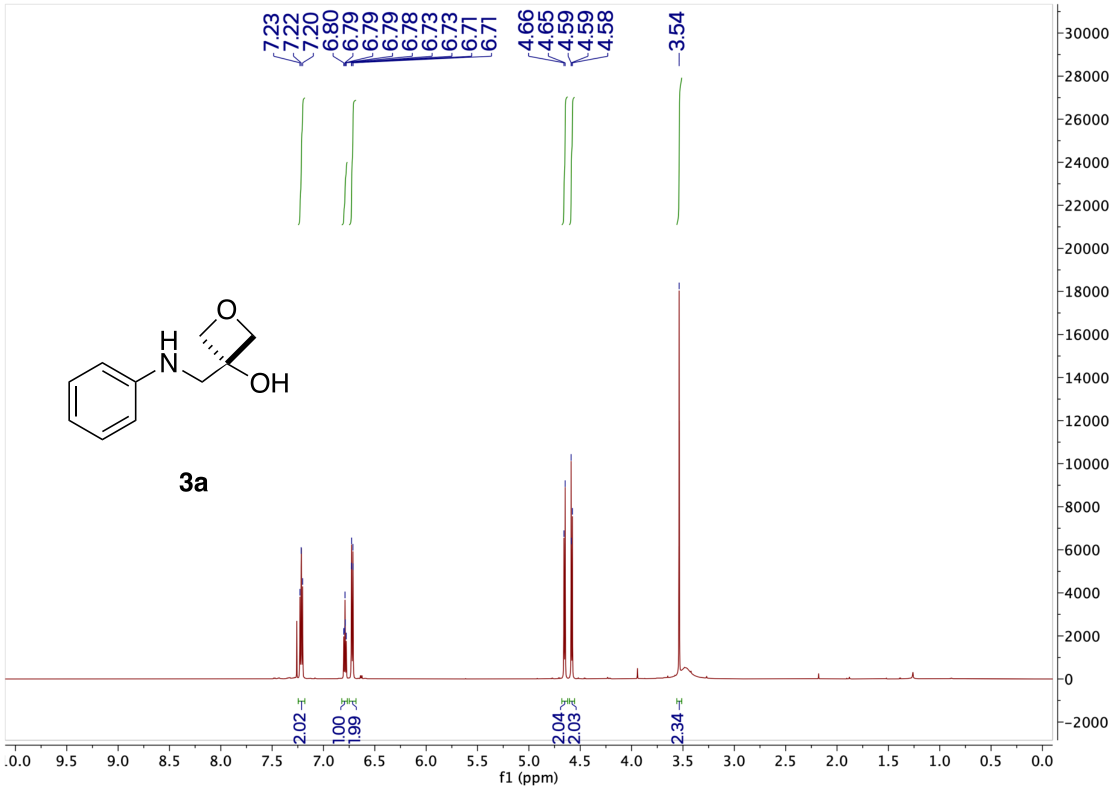
7.23
7.22
7.20
6.80
6.79
6.79
6.78
6.73
6.73
6.71
6.71
4.66
4.65
4.59
4.59
4.58
3.54
H
N
O
OH
3a
2.02
1.00
1.99
2.04
2.03
2.34
f1 (ppm)

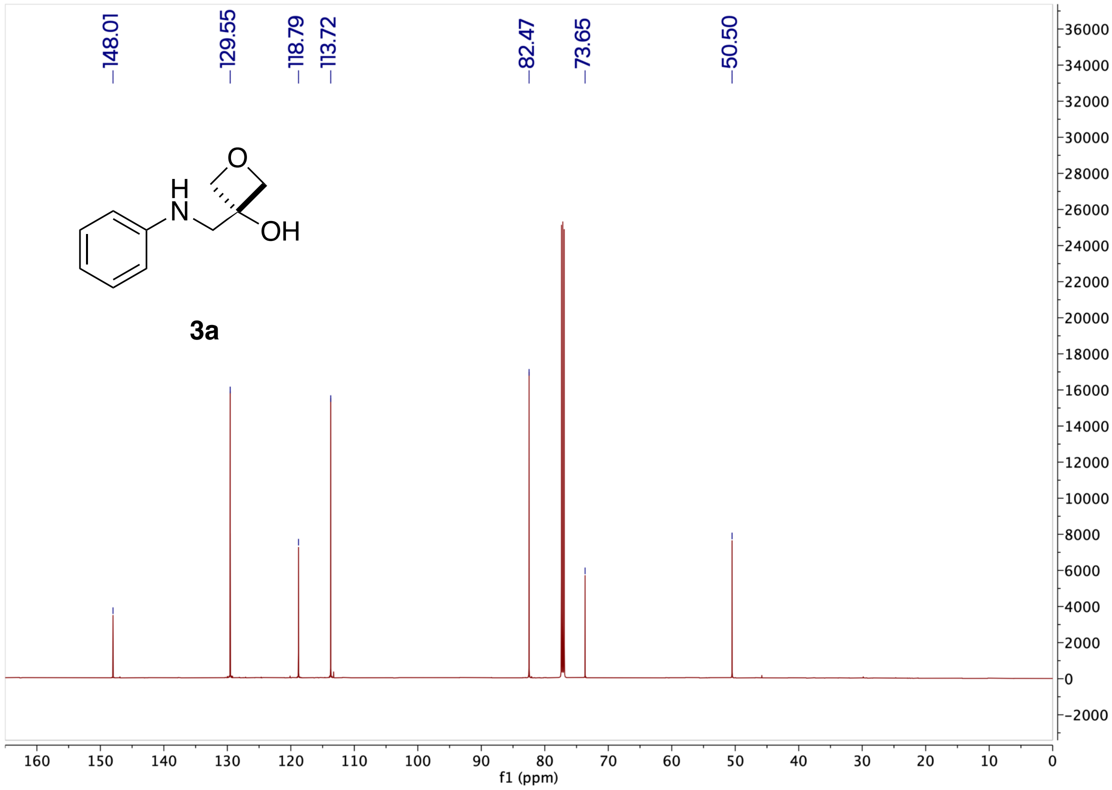
148.01
129.55
118.79
113.72
82.47
73.65
50.50
H
N
O
OH
3a
f1 (ppm)

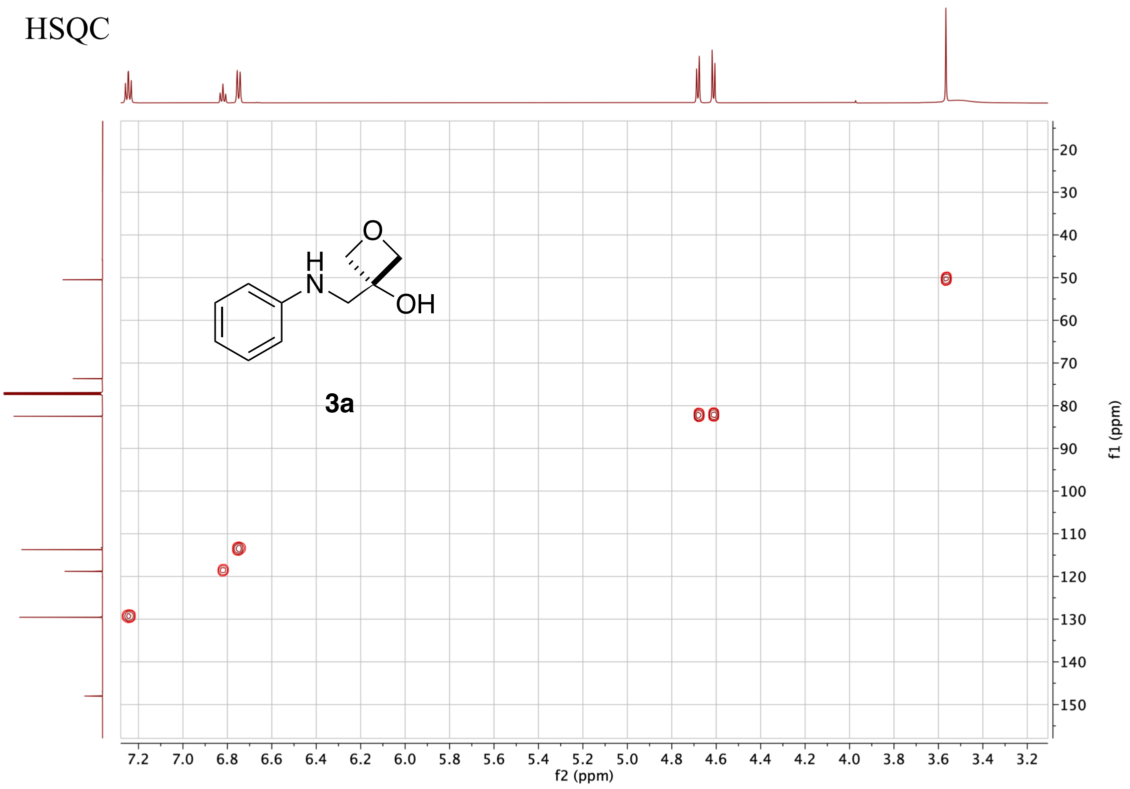
HSQC
3a
OH
O
H
N
f2 (ppm)
f1 (ppm)

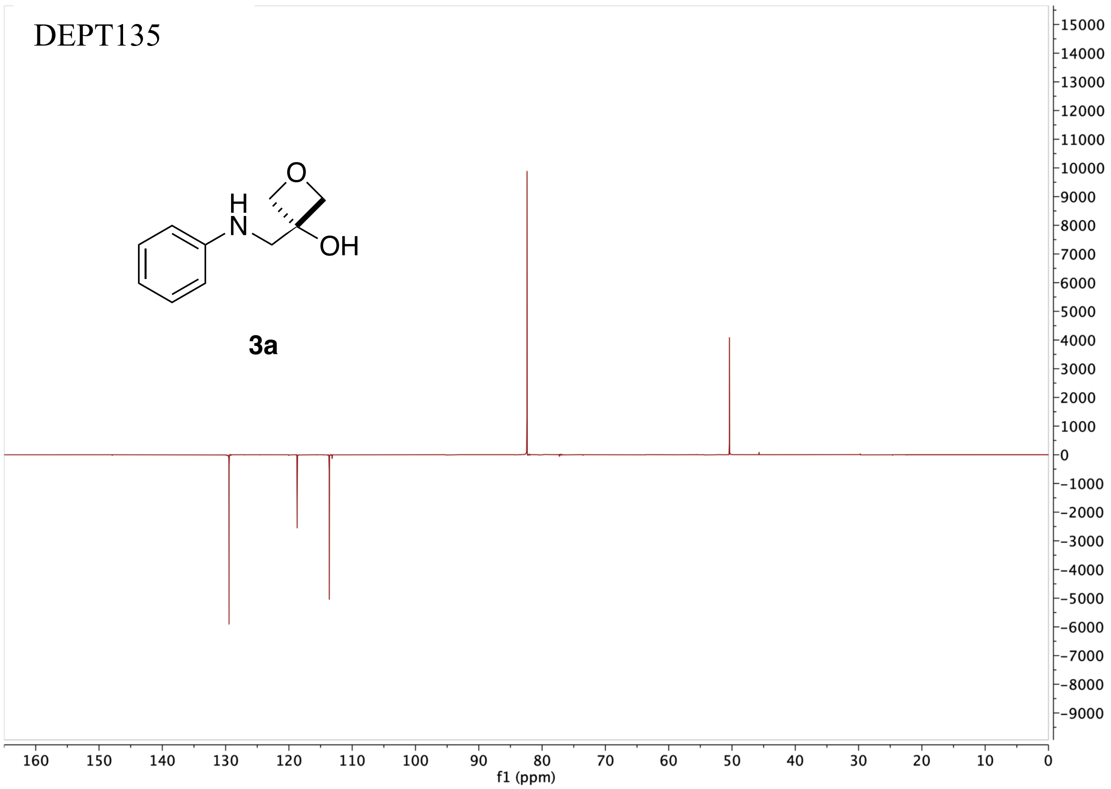
DEPT135
3a
OH
O
H
N
f1 (ppm)

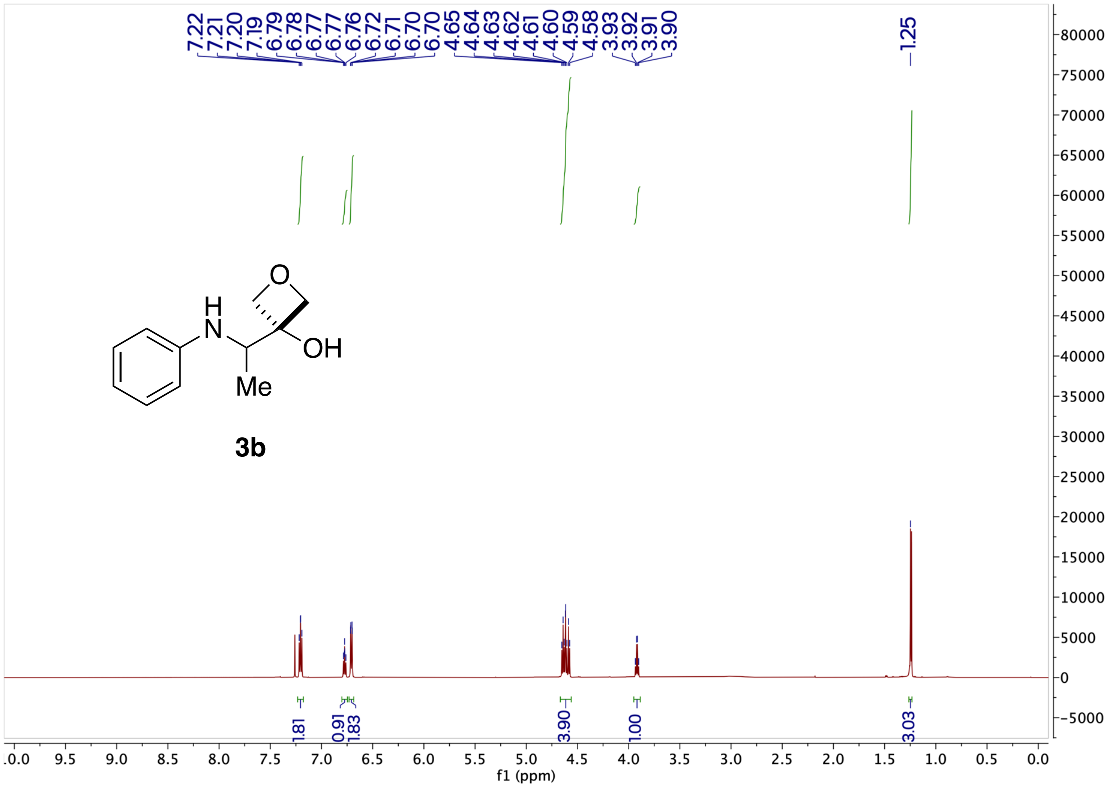
7.22
7.21
7.20
7.19
6.79
6.78
6.77
6.77
6.76
6.72
6.71
6.70
6.70
4.65
4.64
4.63
4.62
4.61
4.60
4.59
4.58
3.93
3.92
3.91
3.90
1.25
H
N
O
OH
Me
3b
1.81
0.91
1.83
3.90
1.00
3.03
f1 (ppm)

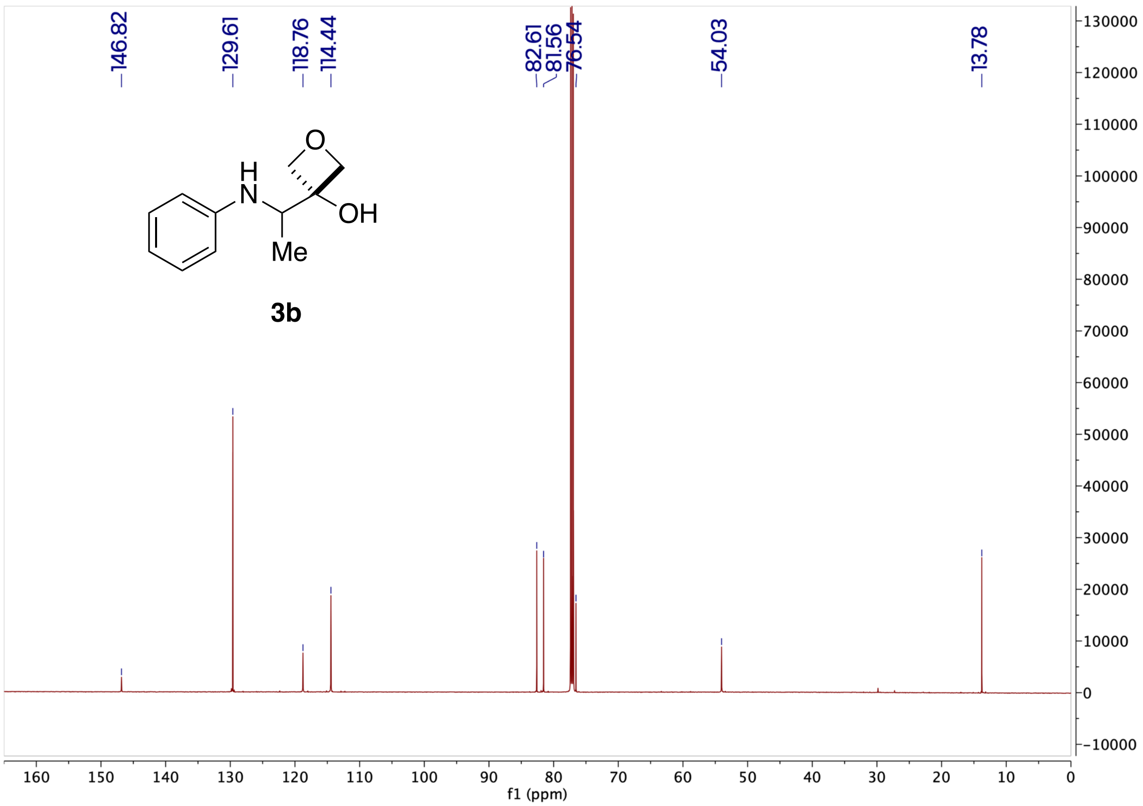
146.82
129.61
118.76
114.44
82.61
81.56
76.54
54.03
13.78
H
N
O
OH
Me
3b
f1 (ppm)

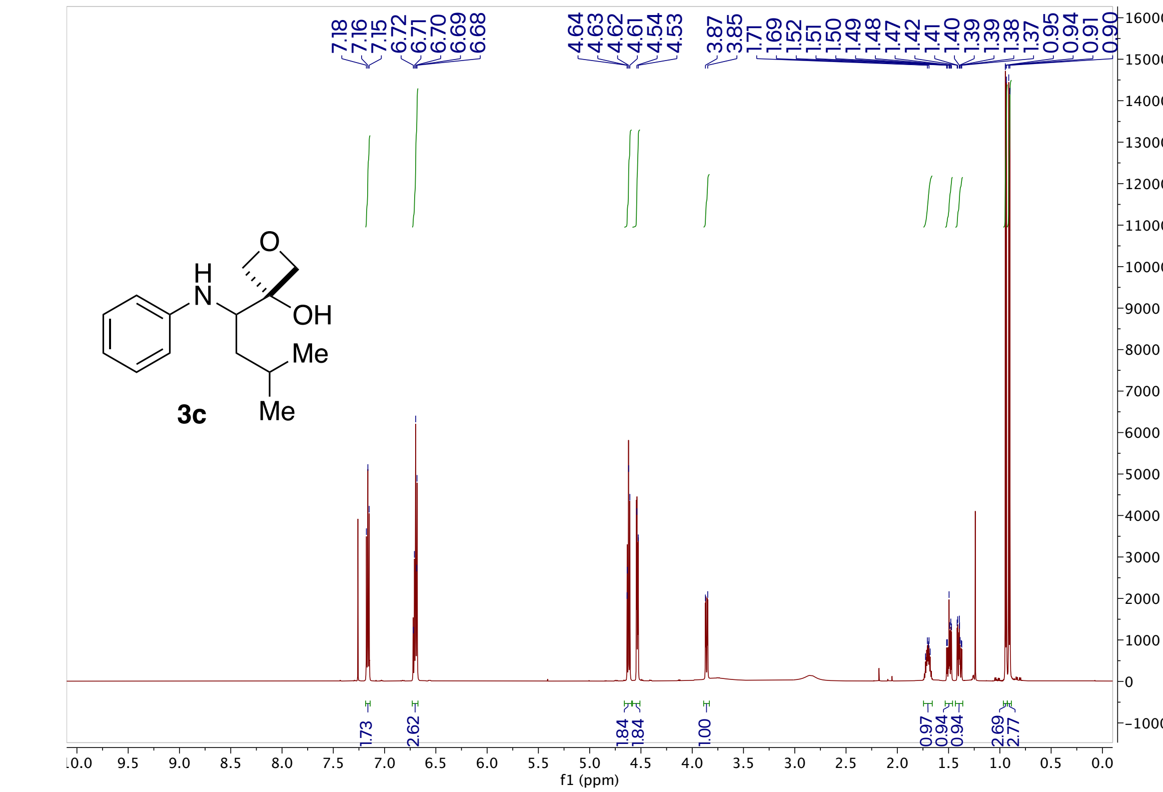
7.18
7.16
7.15
6.72
6.71
6.70
6.69
6.68
4.64
4.63
4.62
4.61
4.54
4.53
3.87
3.85
1.71
1.69
1.52
1.51
1.50
1.49
1.48
1.47
1.42
1.41
1.40
1.39
1.38
1.37
0.95
0.94
0.91
0.90
H
N
O
OH
Me
Me
3c
1.73
2.62
1.84
1.84
1.00
0.97
0.94
0.94
2.69
2.77
f1 (ppm)

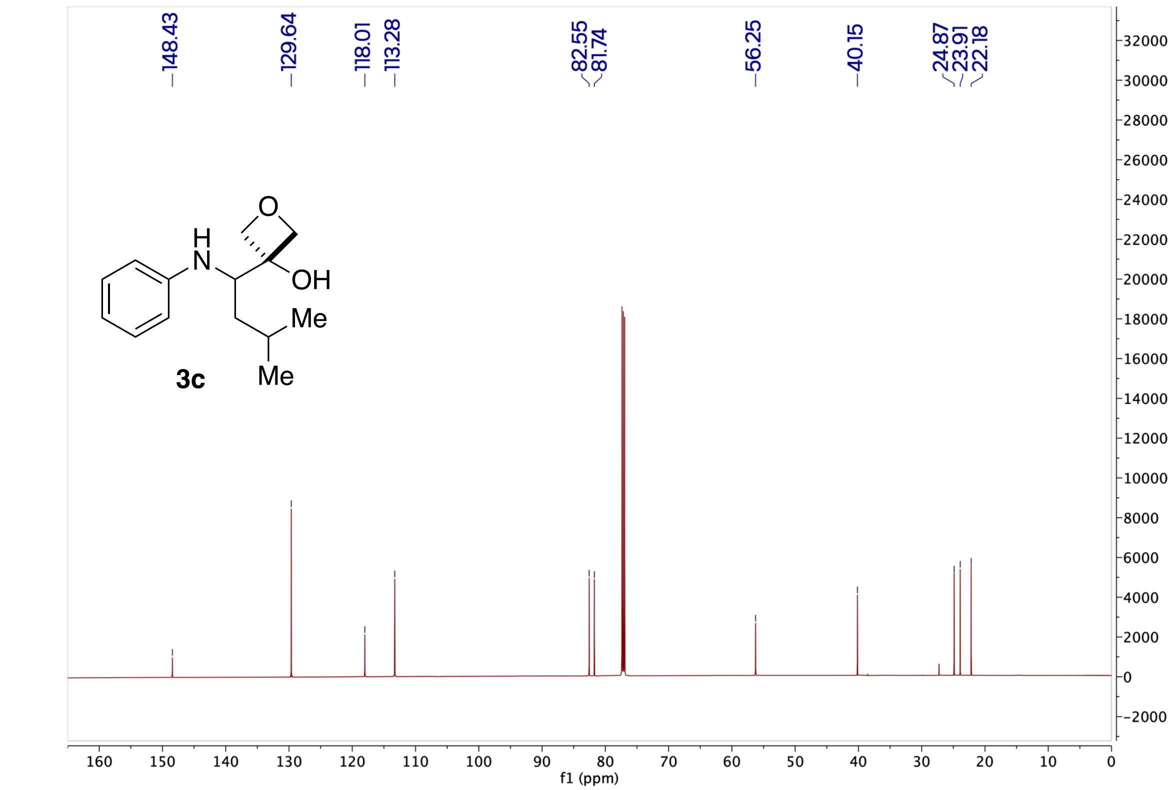
148.43
129.64
118.01
113.28
82.55
81.74
56.25
40.15
24.87
23.91
22.18
H
N
O
OH
Me
Me
3c
f1 (ppm)

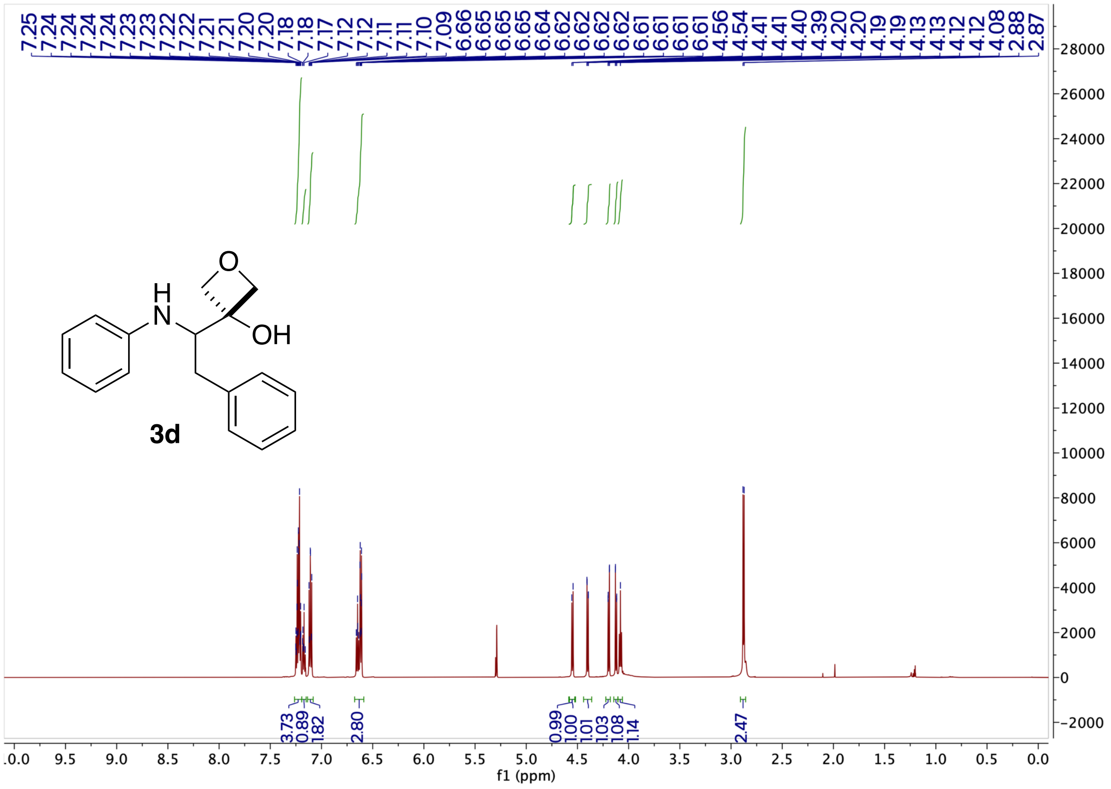
7.25 7.24 7.24 7.24 7.24 7.23 7.23 7.22 7.22 7.21 7.21 7.20 7.20 7.18 7.18 7.17 7.12 7.12 7.11 7.11 7.10 7.09 6.66 6.65 6.65 6.65 6.64 6.62 6.62 6.62 6.62 6.61 6.61 6.61 6.61 4.56 4.54 4.41 4.41 4.40 4.39 4.20 4.20 4.19 4.19 4.13 4.13 4.12 4.12 4.08 2.88 2.87
3.73 0.89 1.82 2.80 0.99 1.00 1.01 1.03 1.08 1.14 2.47
3d
f1 (ppm)

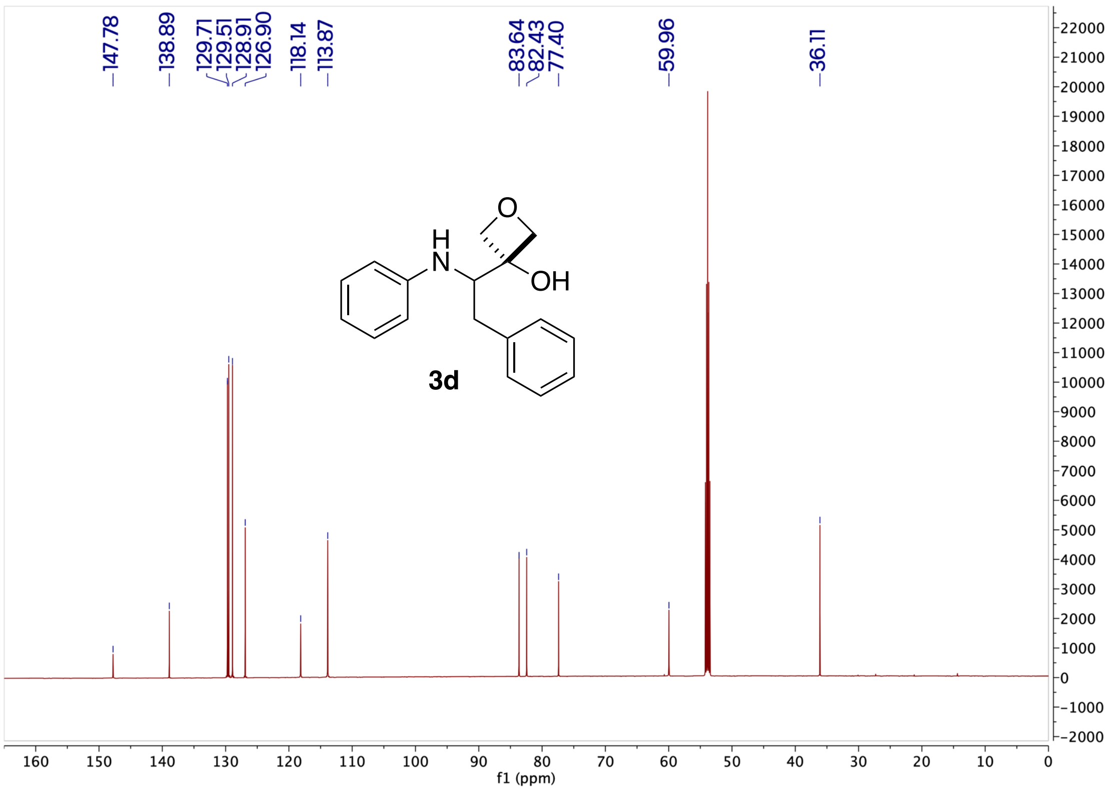
147.78 138.89 129.71 129.51 128.91 126.90 118.14 113.87 83.64 82.43 77.40 59.96 36.11
3d
f1 (ppm)

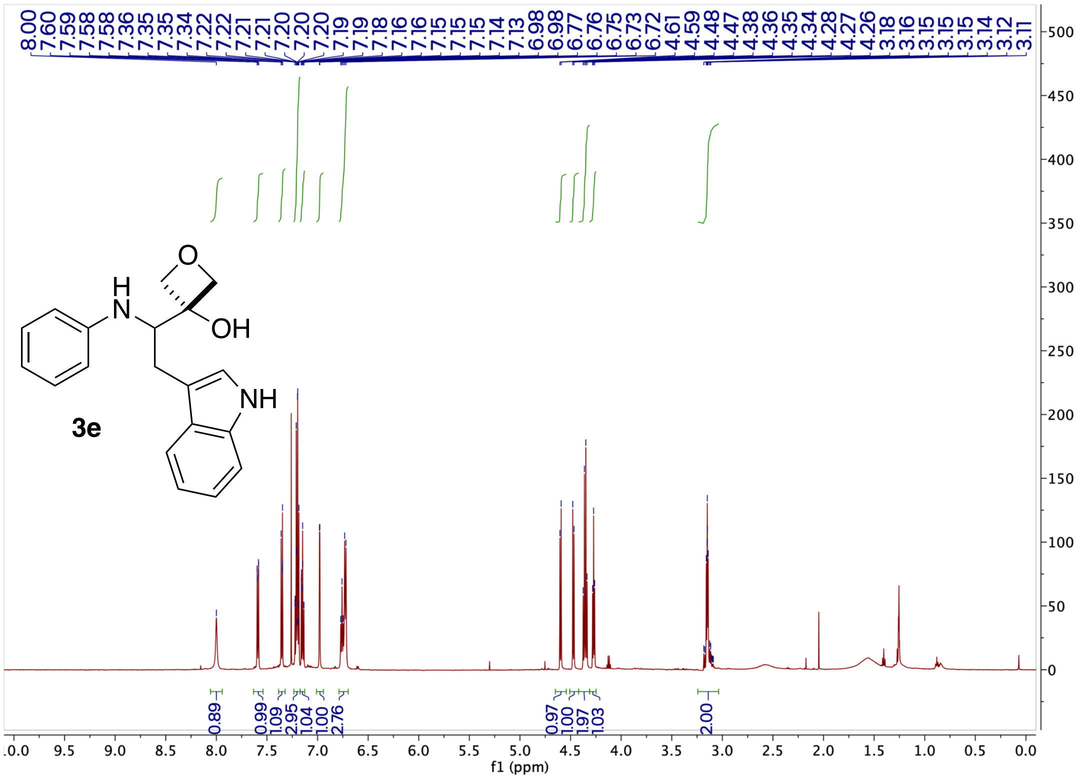
3e
NH
OH
O
H
N
0.89
0.99
1.09
2.95
1.04
1.00
2.76
0.97
1.00
1.97
1.03
2.00
f1 (ppm)

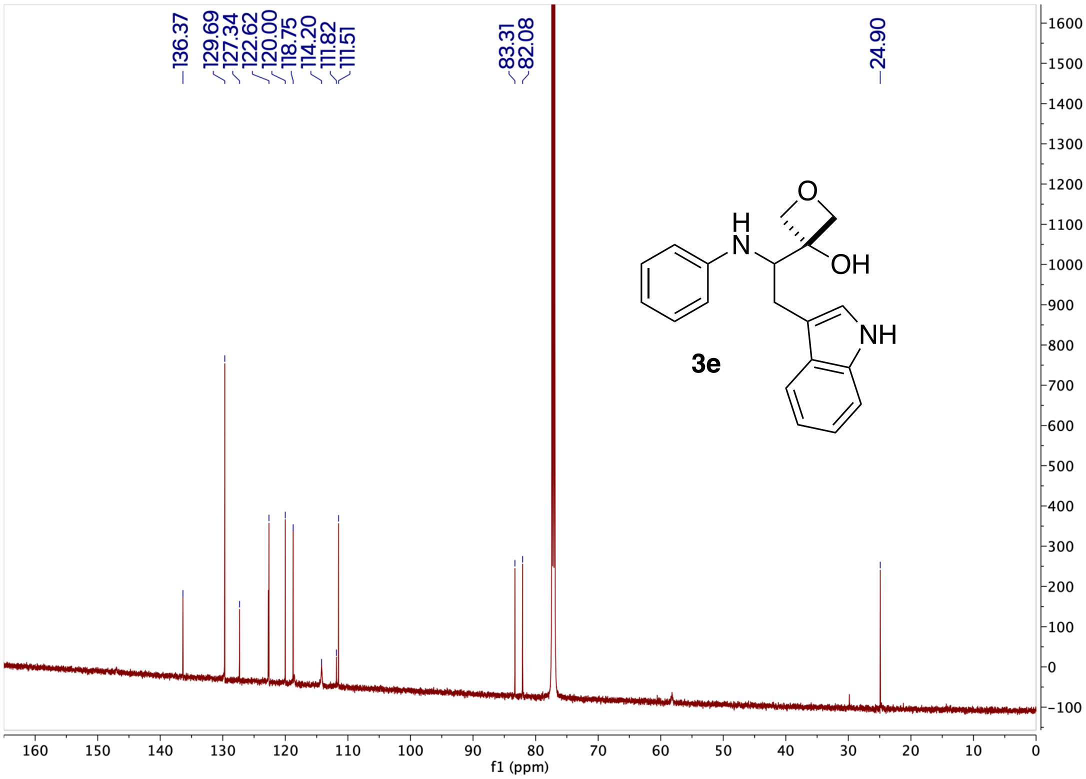
136.37
129.69
127.34
122.62
120.00
118.75
114.20
111.82
111.51
83.31
82.08
24.90
3e
f1 (ppm)

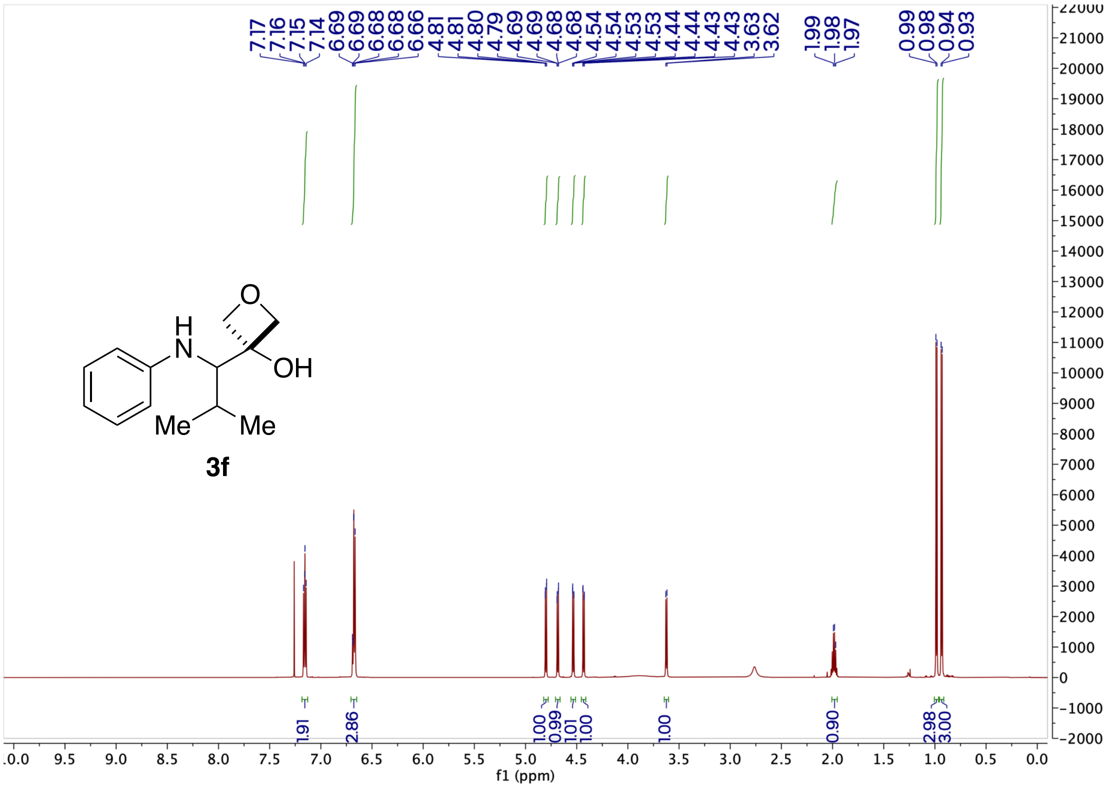
7.17
7.16
7.15
7.14
6.69
6.69
6.68
6.68
6.66
4.81
4.81
4.80
4.79
4.69
4.69
4.68
4.68
4.54
4.54
4.53
4.53
4.44
4.44
4.43
4.43
3.63
3.62
1.99
1.98
1.97
0.99
0.98
0.94
0.93
H
N
O
OH
Me
Me
3f
1.91
2.86
1.00
0.99
1.01
1.00
1.00
0.90
2.98
3.00
f1 (ppm)

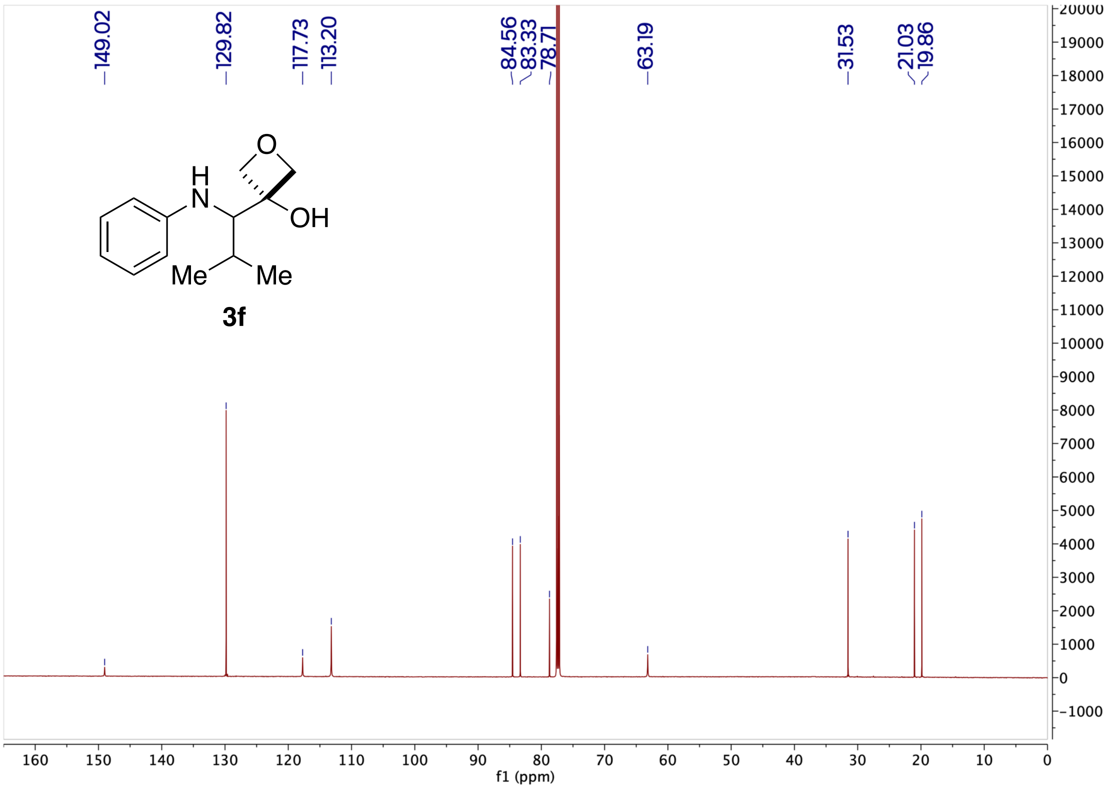
149.02
129.82
117.73
113.20
84.56
83.33
78.71
63.19
31.53
21.03
19.86
H
N
O
OH
Me
Me
3f
f1 (ppm)

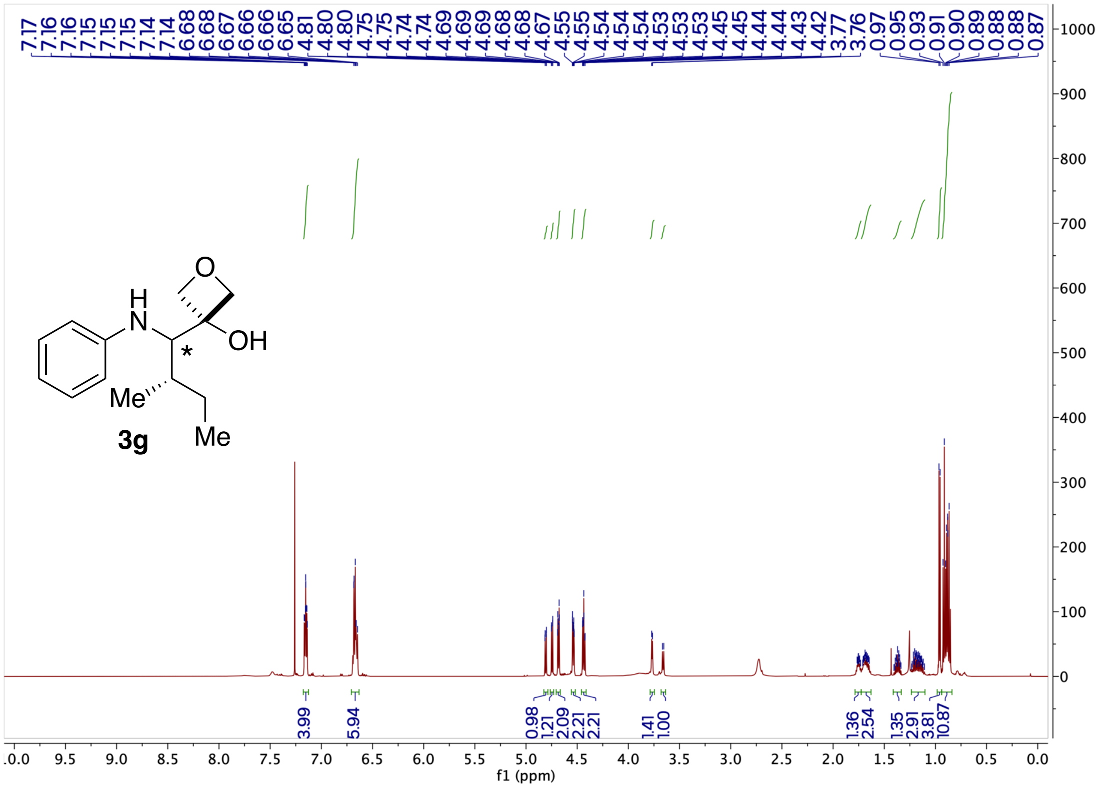
3g
f1 (ppm)

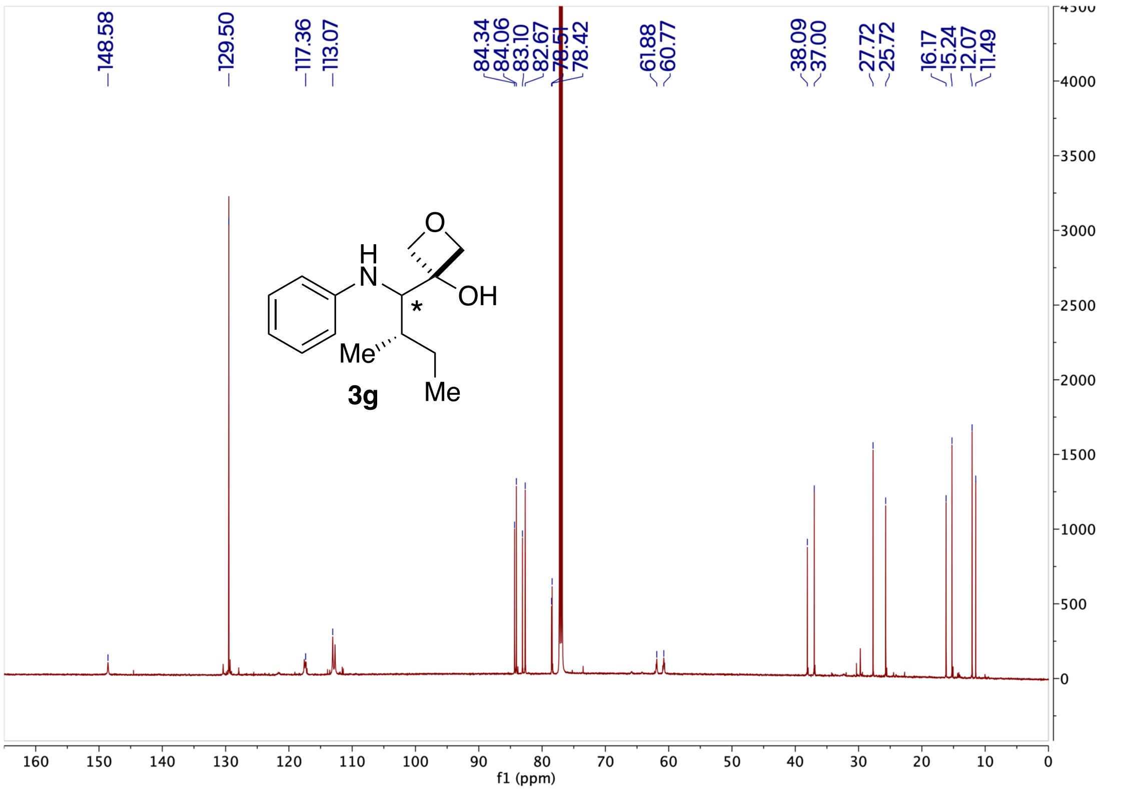
3g
f1 (ppm)

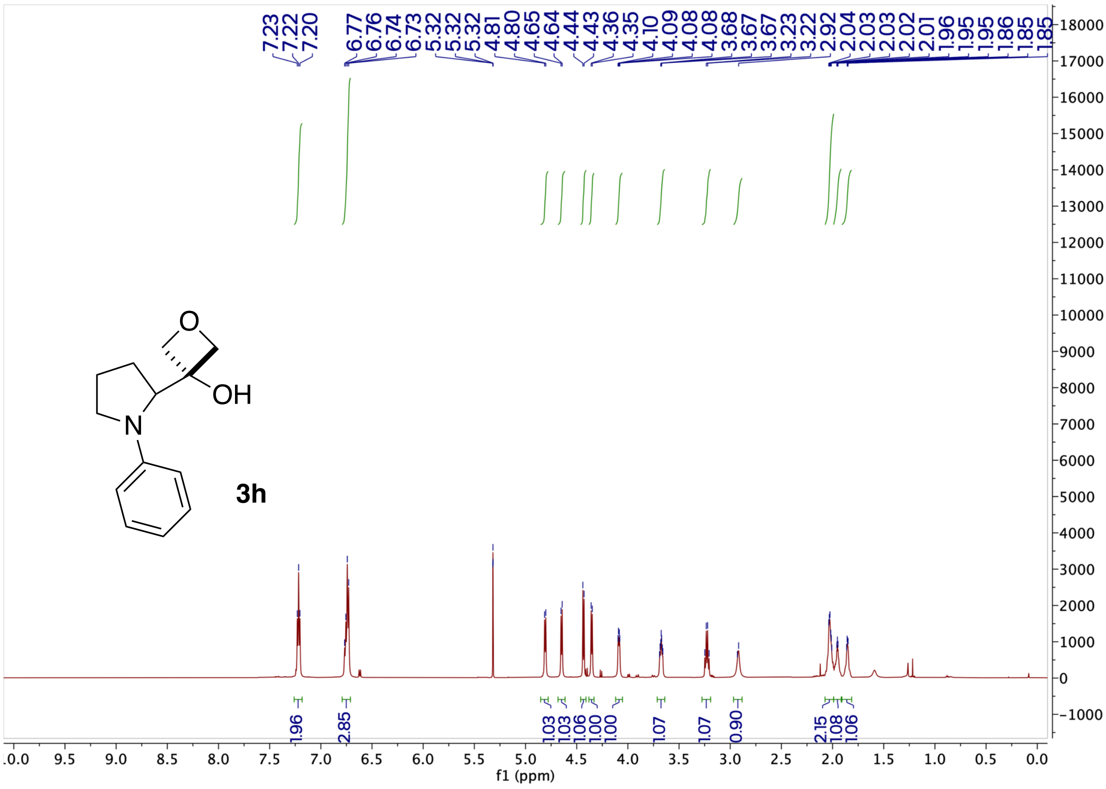
7.23
7.22
7.20
6.77
6.76
6.74
6.73
5.32
5.32
5.32
4.81
4.80
4.65
4.64
4.44
4.43
4.36
4.35
4.10
4.09
4.08
4.08
3.68
3.67
3.67
3.23
3.22
2.92
2.04
2.03
2.03
2.02
2.01
1.96
1.95
1.95
1.86
1.85
1.85
OH
N
O
3h
1.96
2.85
1.03
1.03
1.06
1.00
1.00
1.07
1.07
0.90
2.15
1.08
1.06
f1 (ppm)

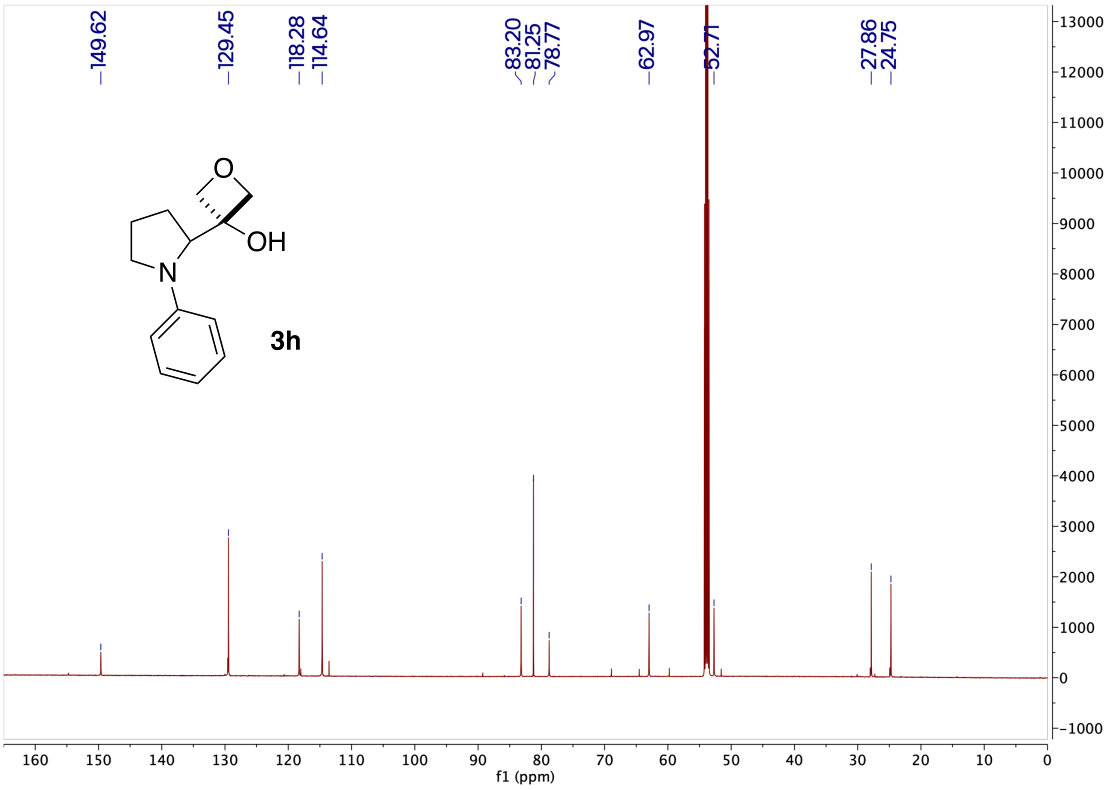
149.62
129.45
118.28
114.64
83.20
81.25
78.77
62.97
52.71
27.86
24.75
O
OH
N
3h
f1 (ppm)

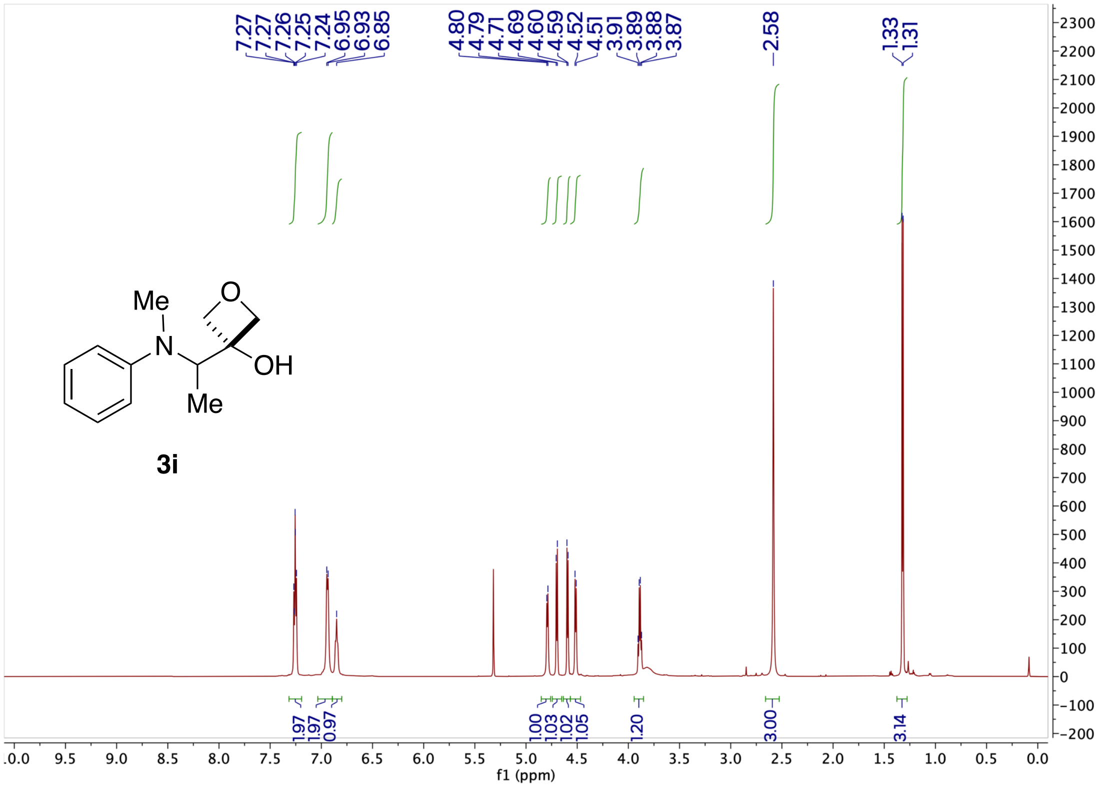

7.27
7.27
7.26
7.25
7.24
6.95
6.93
6.85
4.80
4.79
4.71
4.69
4.60
4.59
4.52
4.51
3.91
3.89
3.88
3.87
2.58
1.33
1.31
Me
N
O
OH
Me
3i
1.97
1.97
0.97
1.00
1.03
1.02
1.05
1.20
3.00
3.14
f1 (ppm)

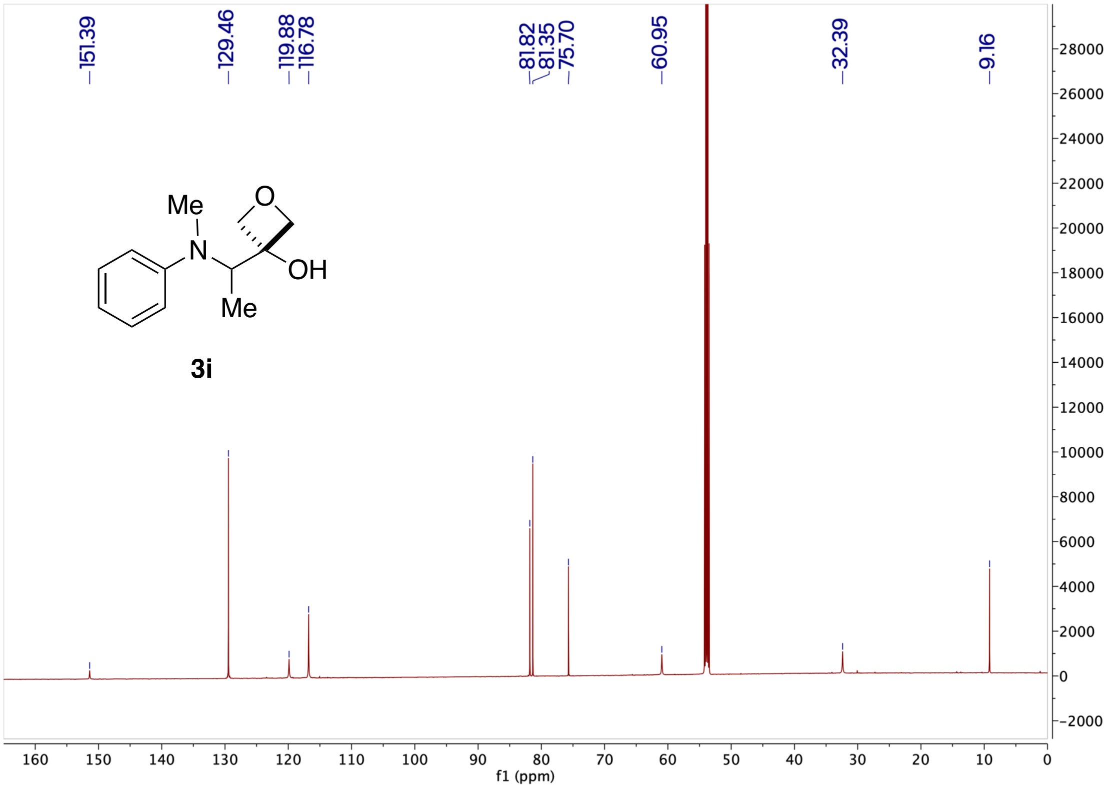

151.39
129.46
119.88
116.78
81.82
81.35
75.70
60.95
32.39
9.16
Me
N
O
OH
Me
3i
f1 (ppm)

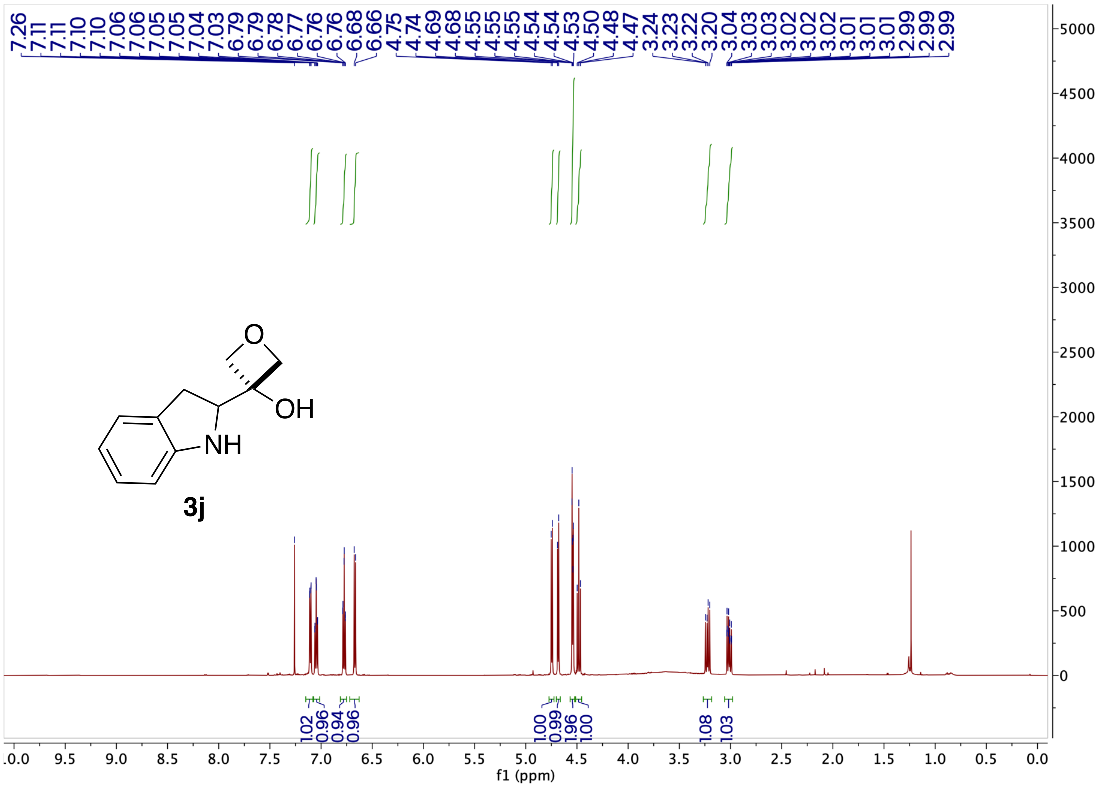

7.26
7.11
7.11
7.10
7.10
7.06
7.06
7.05
7.05
7.04
7.03
6.79
6.79
6.78
6.77
6.76
6.76
6.68
6.66
4.75
4.74
4.69
4.68
4.55
4.55
4.55
4.54
4.54
4.53
4.50
4.48
4.47
3.24
3.23
3.22
3.20
3.04
3.03
3.03
3.02
3.02
3.02
3.01
3.01
3.01
2.99
2.99
OH
NH
3j
1.02
0.96
0.94
0.96
1.00
0.99
1.96
1.00
1.08
1.03
f1 (ppm)

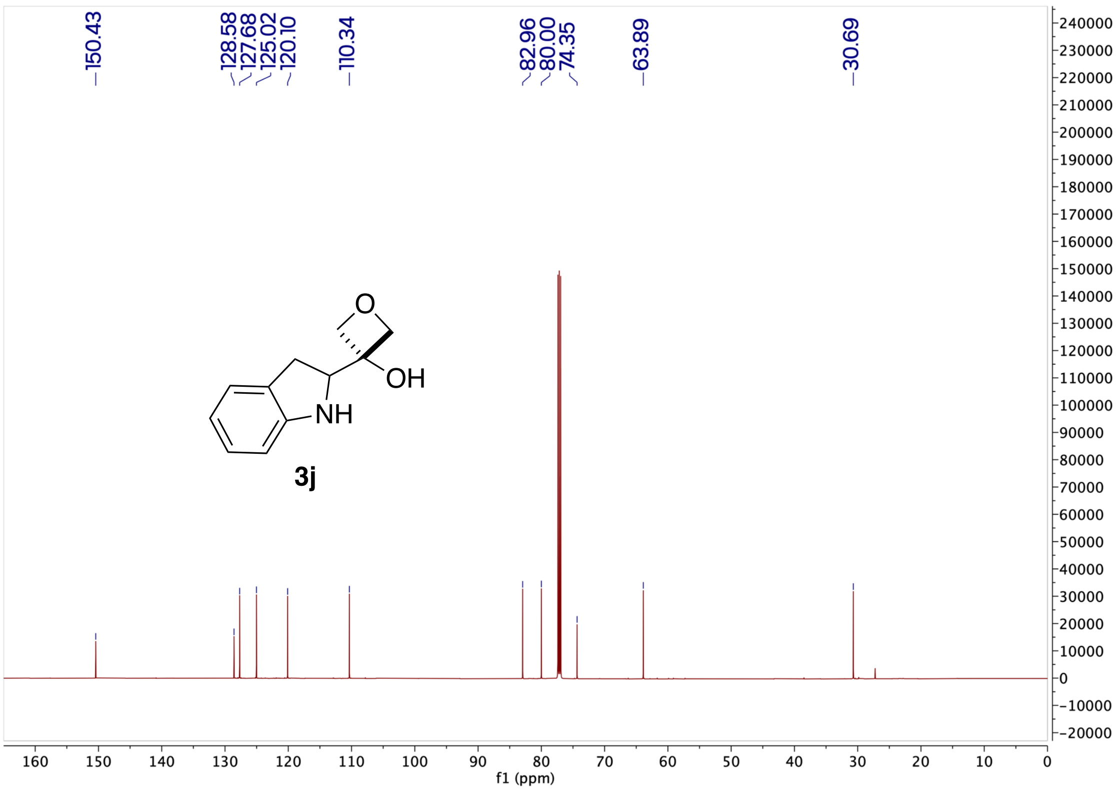

150.43
128.58
127.68
125.02
120.10
110.34
82.96
80.00
74.35
63.89
30.69
O
OH
NH
3j
f1 (ppm)

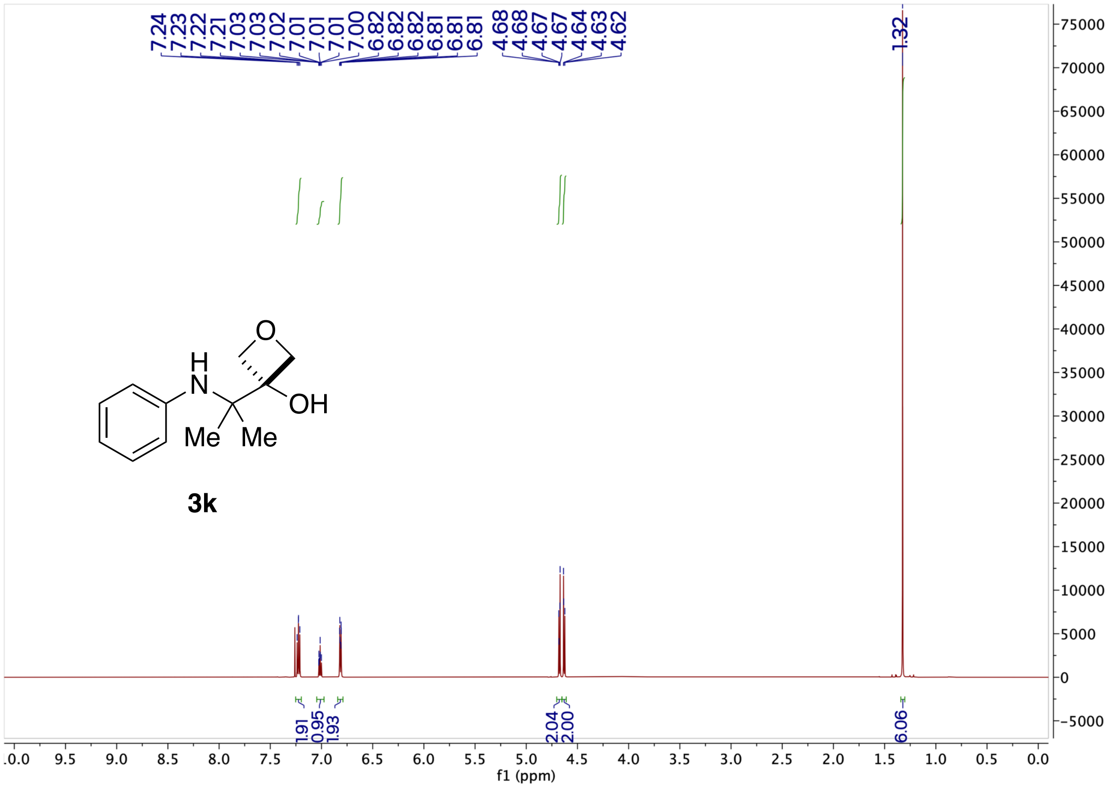
7.24
7.23
7.22
7.21
7.03
7.03
7.02
7.01
7.01
7.01
7.00
6.82
6.82
6.82
6.81
6.81
6.81
4.68
4.68
4.67
4.67
4.64
4.63
4.62
1.32
H
N
O
OH
Me
Me
3k
1.91
0.95
1.93
2.04
2.00
6.06
f1 (ppm)

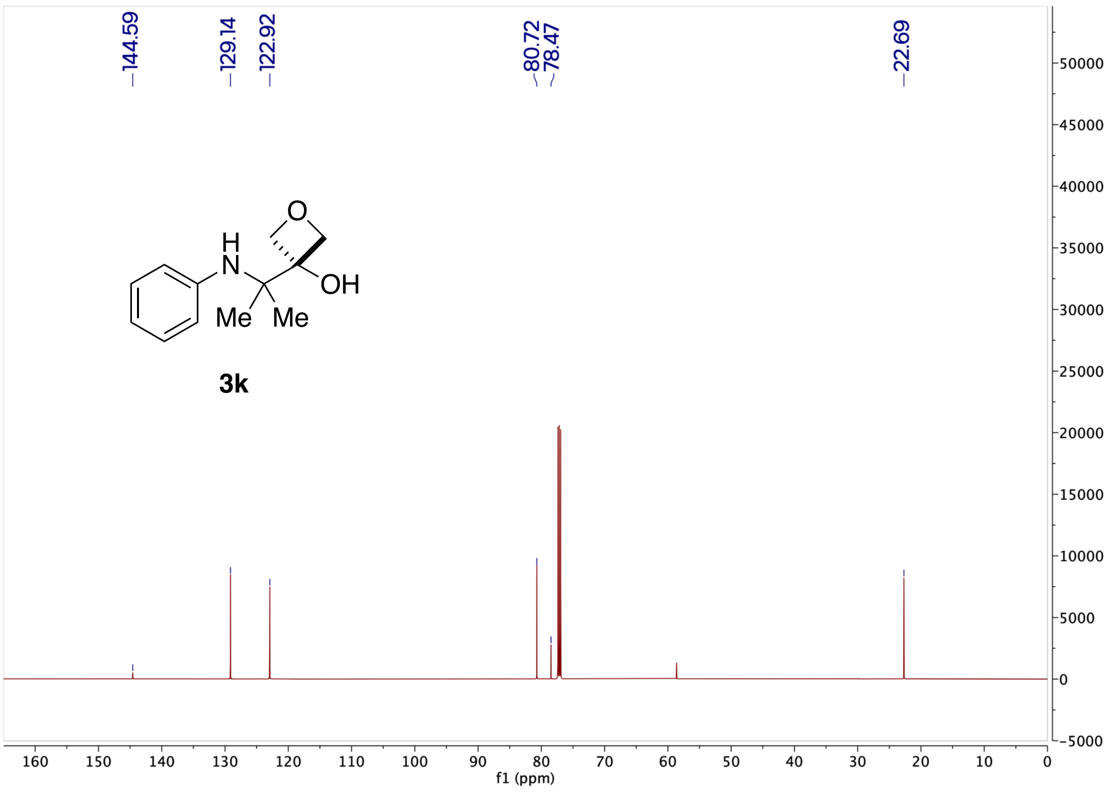
144.59
129.14
122.92
80.72
78.47
22.69
H
N
O
OH
Me
Me
3k
f1 (ppm)

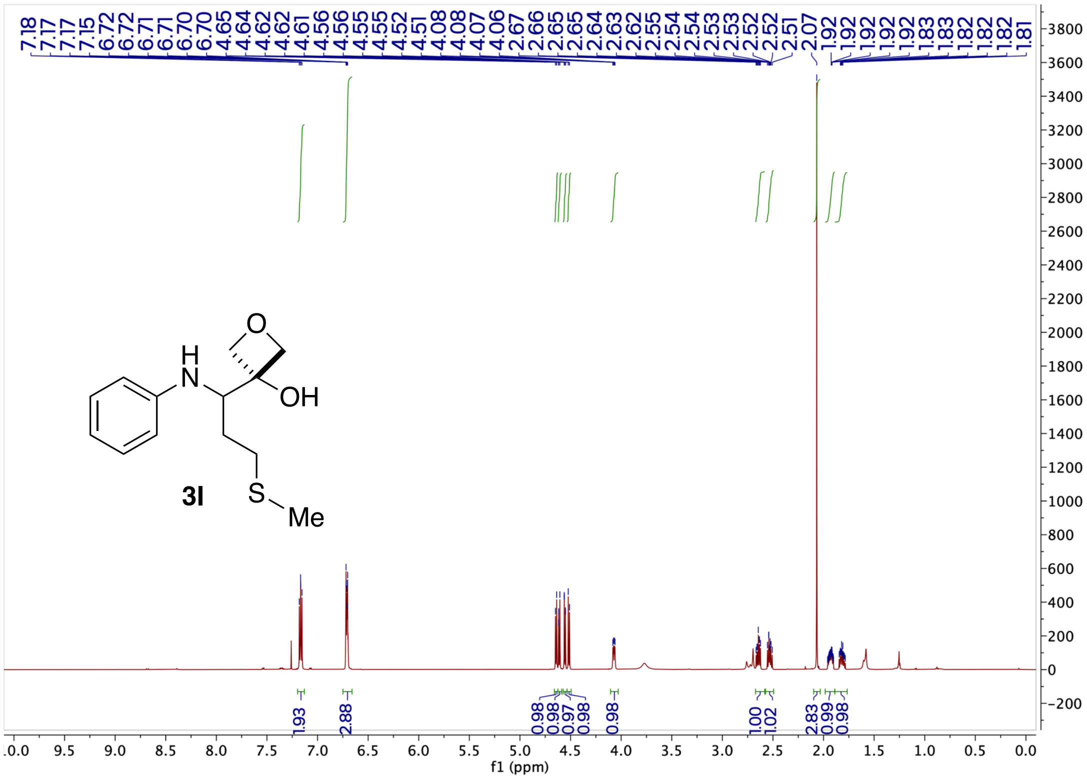
7.18
7.17
7.17
7.15
6.72
6.72
6.71
6.71
6.70
6.70
4.65
4.64
4.62
4.62
4.61
4.56
4.56
4.55
4.55
4.52
4.51
4.08
4.08
4.07
4.06
2.67
2.66
2.65
2.65
2.64
2.63
2.62
2.55
2.54
2.54
2.53
2.53
2.52
2.52
2.51
2.07
1.92
1.92
1.92
1.92
1.92
1.83
1.83
1.82
1.82
1.82
1.81
H
N
O
OH
S
Me
3l
1.93
2.88
0.98
0.98
0.97
0.98
0.98
1.00
1.02
2.83
0.99
0.98
f1 (ppm)

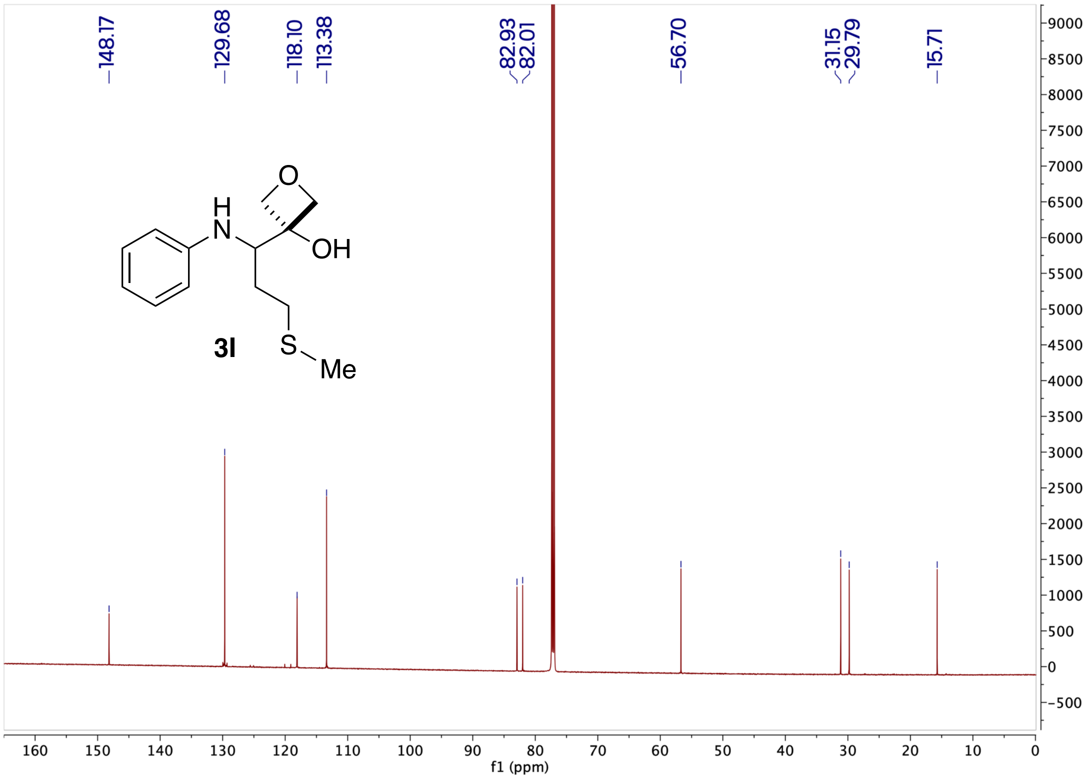
148.17
129.68
118.10
113.38
82.93
82.01
56.70
31.15
29.79
15.71
H
N
O
OH
S
Me
3l
f1 (ppm)

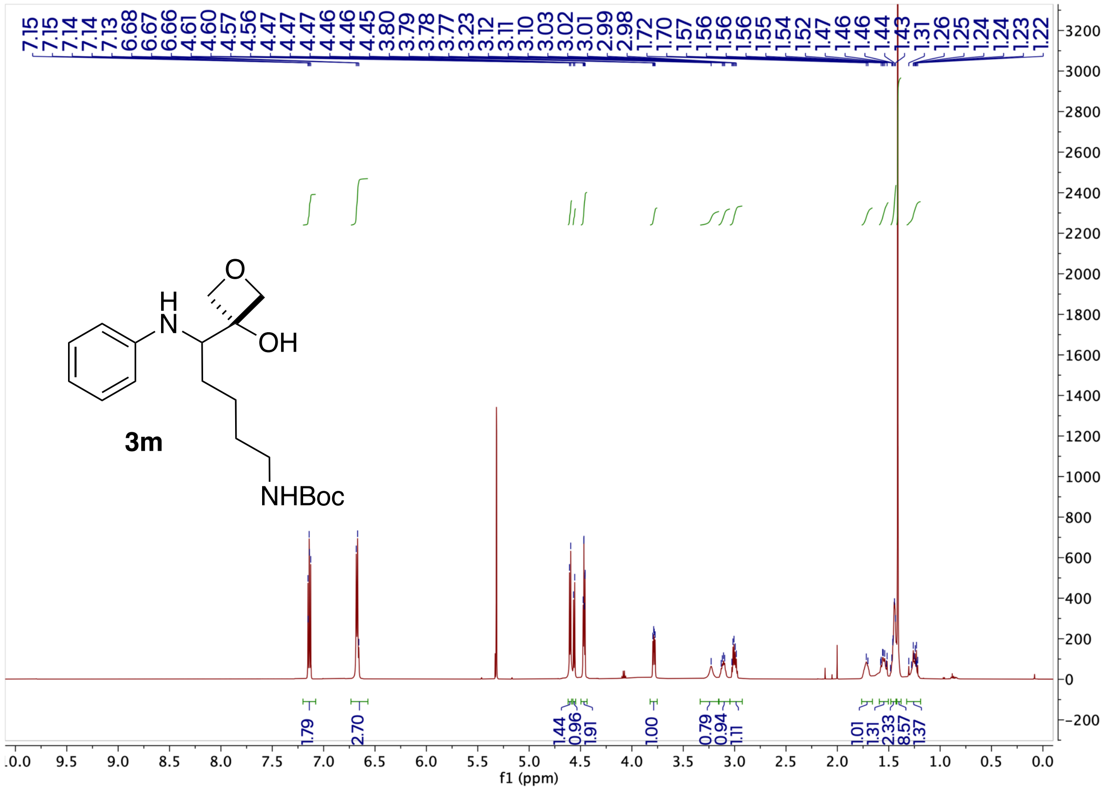

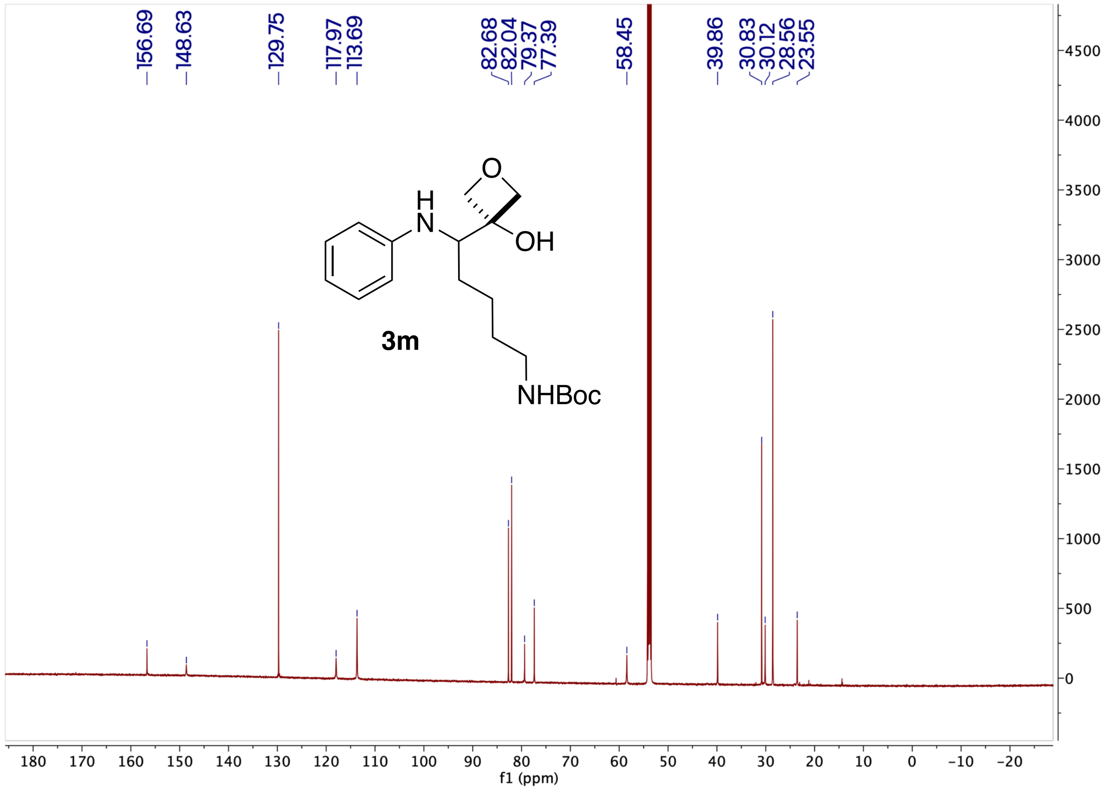

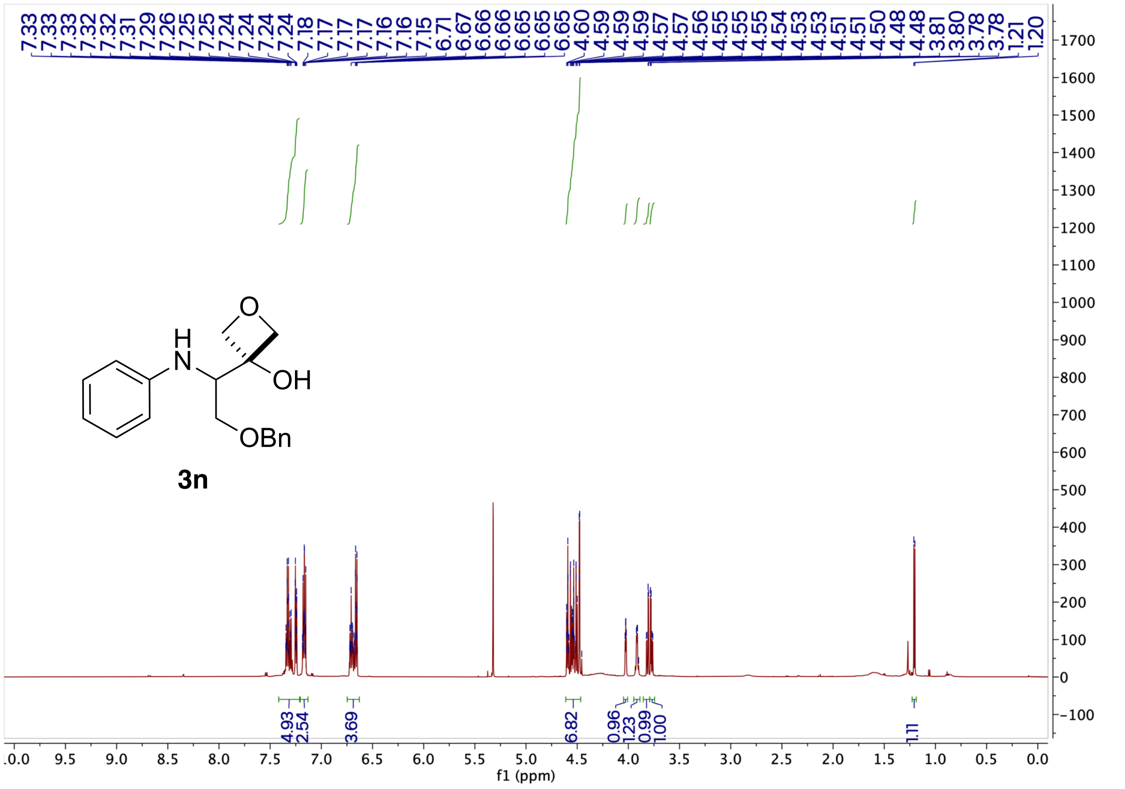
7.33 7.33 7.33 7.32 7.32 7.31 7.29 7.26 7.25 7.25 7.24 7.24 7.24 7.24 7.18 7.17 7.17 7.17 7.16 7.16 7.15 6.71 6.67 6.66 6.66 6.65 6.65 6.65 4.60 4.59 4.59 4.59 4.57 4.56 4.55 4.55 4.54 4.53 4.53 4.51 4.51 4.50 4.48 4.48 3.81 3.80 3.78 3.78 1.21 1.20
H
N
O
OH
OBn
3n
4.93
2.54
3.69
6.82
0.96
1.23
0.99
1.00
1.11
f1 (ppm)

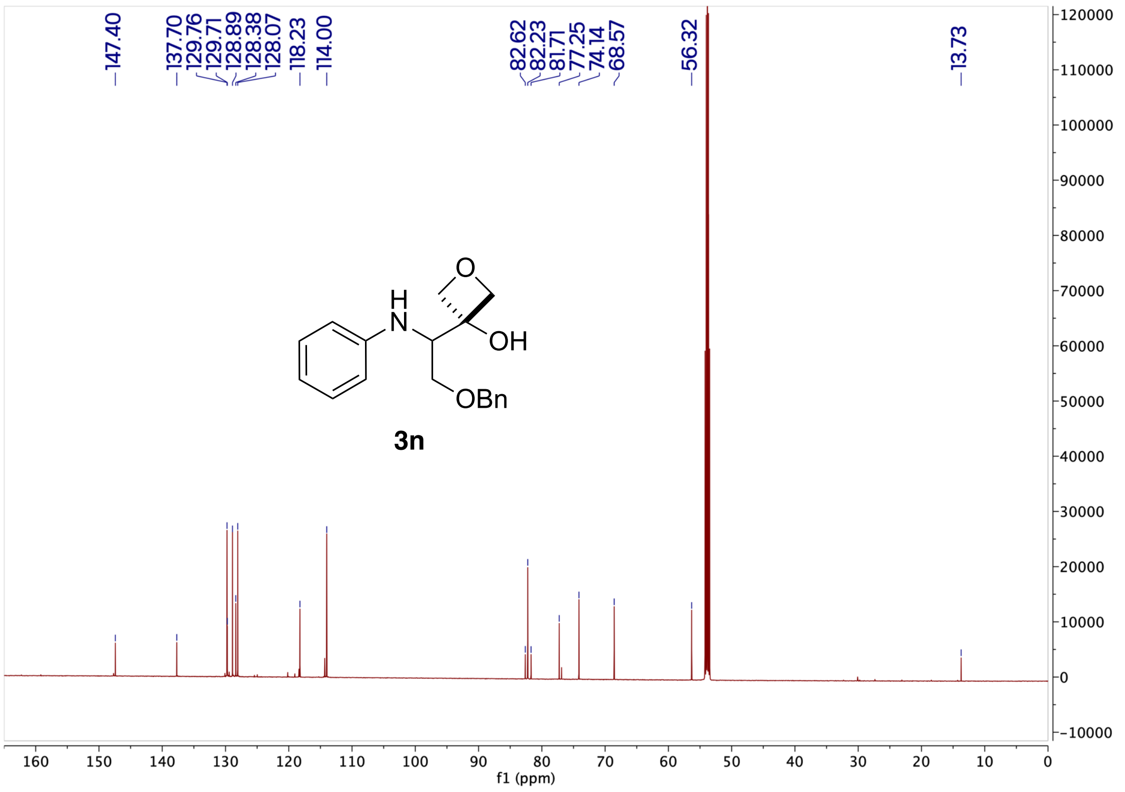
147.40
137.70 129.76 129.71 128.89 128.38 128.07
118.23
114.00
82.62 82.23 81.71 77.25 74.14 68.57
56.32
13.73
H
N
O
OH
OBn
3n
f1 (ppm)

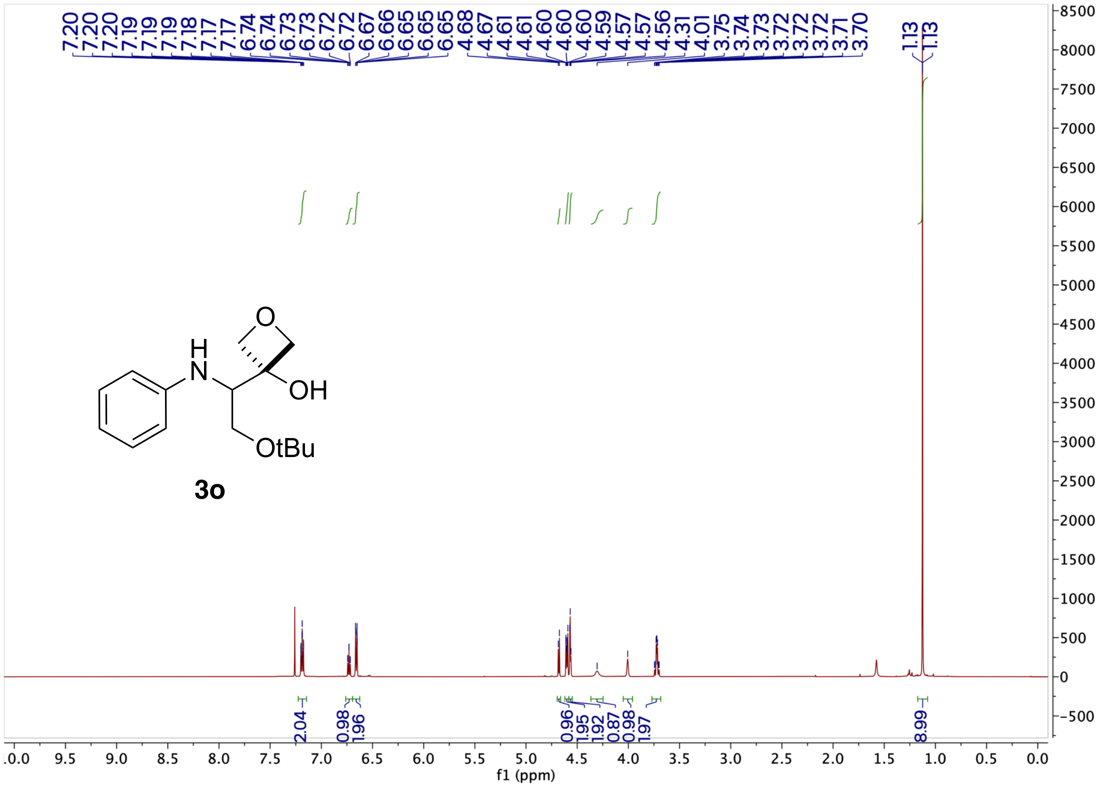
7.20 7.20 7.20 7.19 7.19 7.19 7.18 7.17 7.17 6.74 6.74 6.73 6.72 6.72 6.67 6.66 6.65 6.65 6.65 4.68 4.67 4.61 4.60 4.60 4.60 4.59 4.57 4.57 4.56 4.31 4.01 3.75 3.74 3.73 3.72 3.72 3.72 3.71 3.70
1.13 1.13
H
N
O
OH
OtBu
3o
2.04 0.98 1.96 0.96 1.95 1.92 0.87 0.98 1.97 8.99
f1 (ppm)

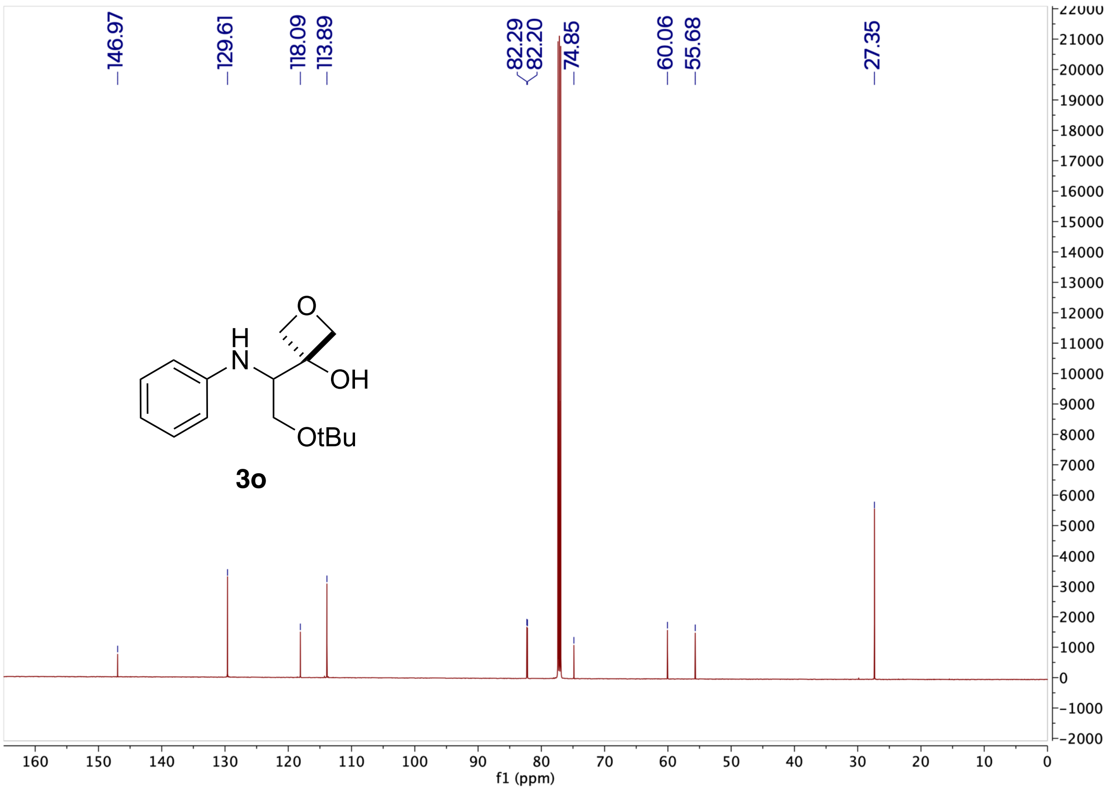
146.97 129.61 118.09 113.89 82.29 82.20 74.85 60.06 55.68 27.35
H
N
O
OH
OtBu
3o
f1 (ppm)

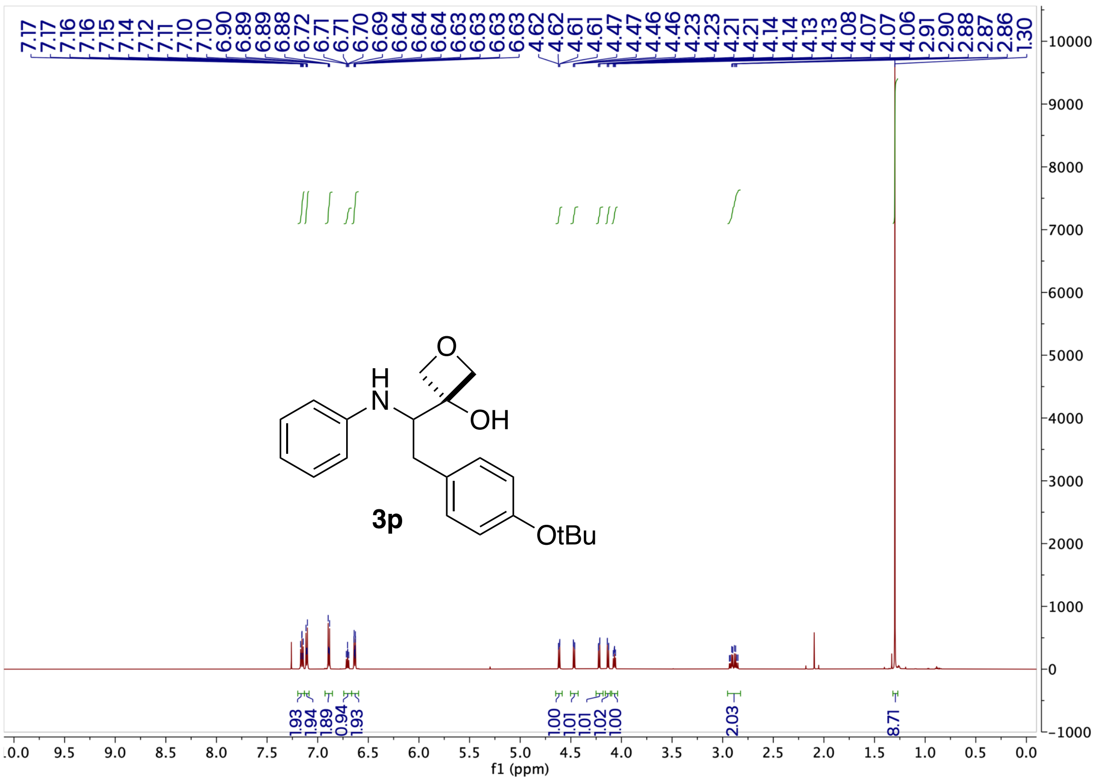

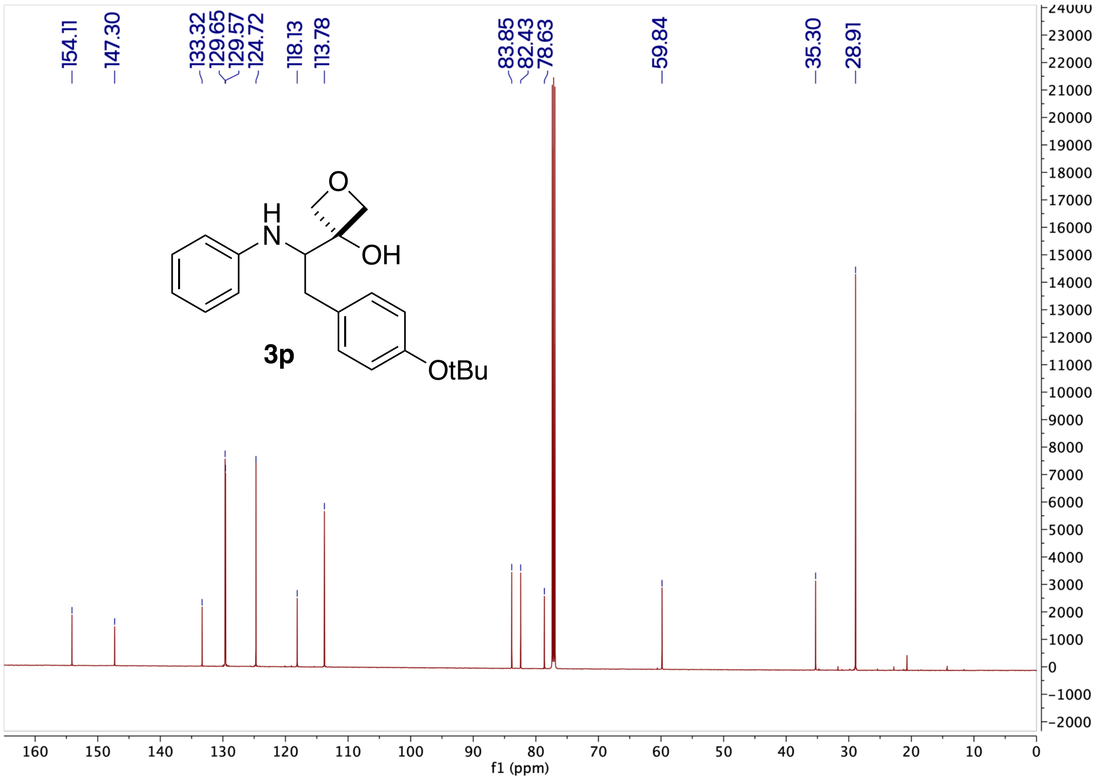

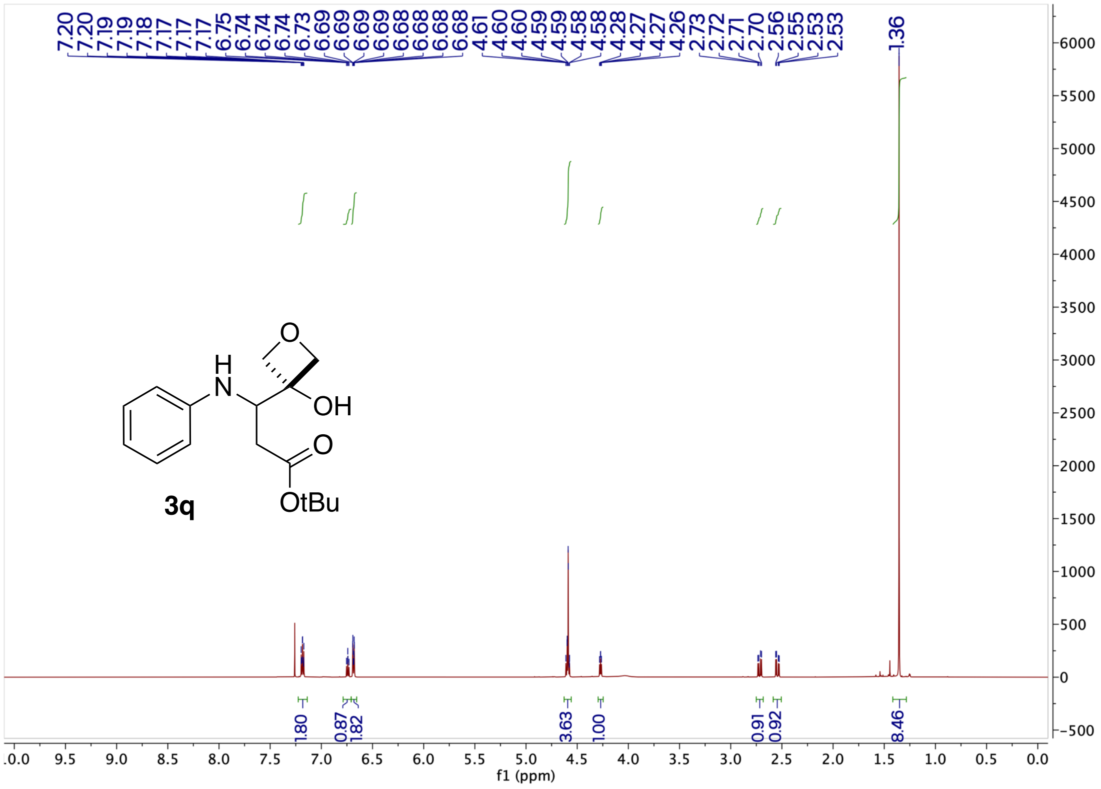
7.20
7.20
7.19
7.19
7.18
7.17
7.17
7.17
6.75
6.74
6.74
6.74
6.73
6.69
6.69
6.69
6.69
6.68
6.68
6.68
6.68
4.61
4.60
4.60
4.59
4.59
4.58
4.58
4.28
4.27
4.27
4.26
2.73
2.72
2.71
2.70
2.56
2.55
2.53
2.53
1.36
3q
OtBu
OH
1.80
0.87
1.82
3.63
1.00
0.91
0.92
8.46
f1 (ppm)

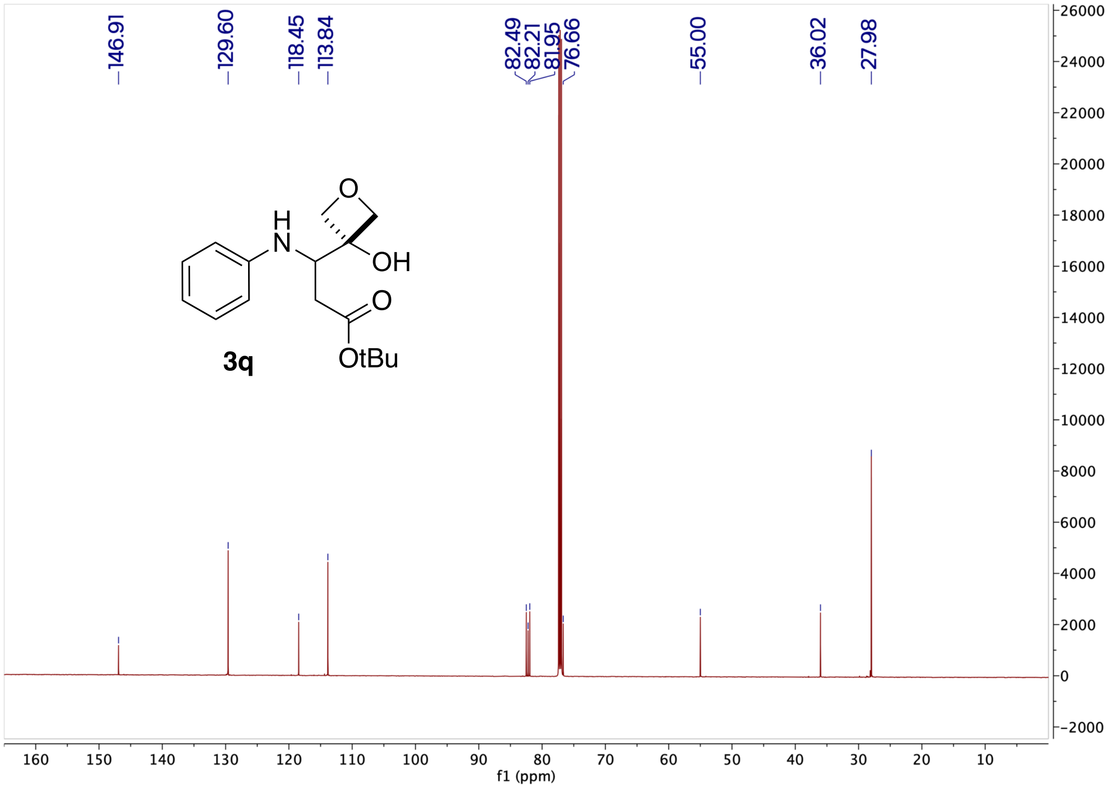
146.91
129.60
118.45
113.84
82.49
82.21
81.95
76.66
55.00
36.02
27.98
3q
OtBu
OH
f1 (ppm)

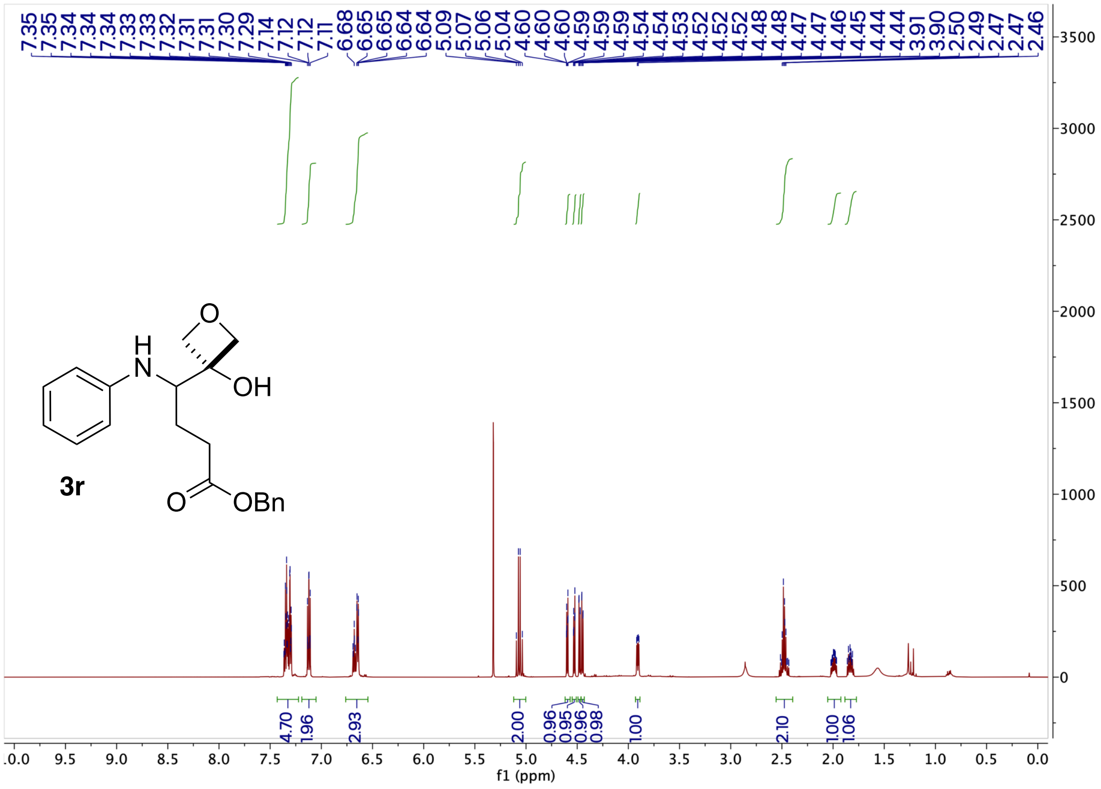
3r
OBn
OH
f1 (ppm)

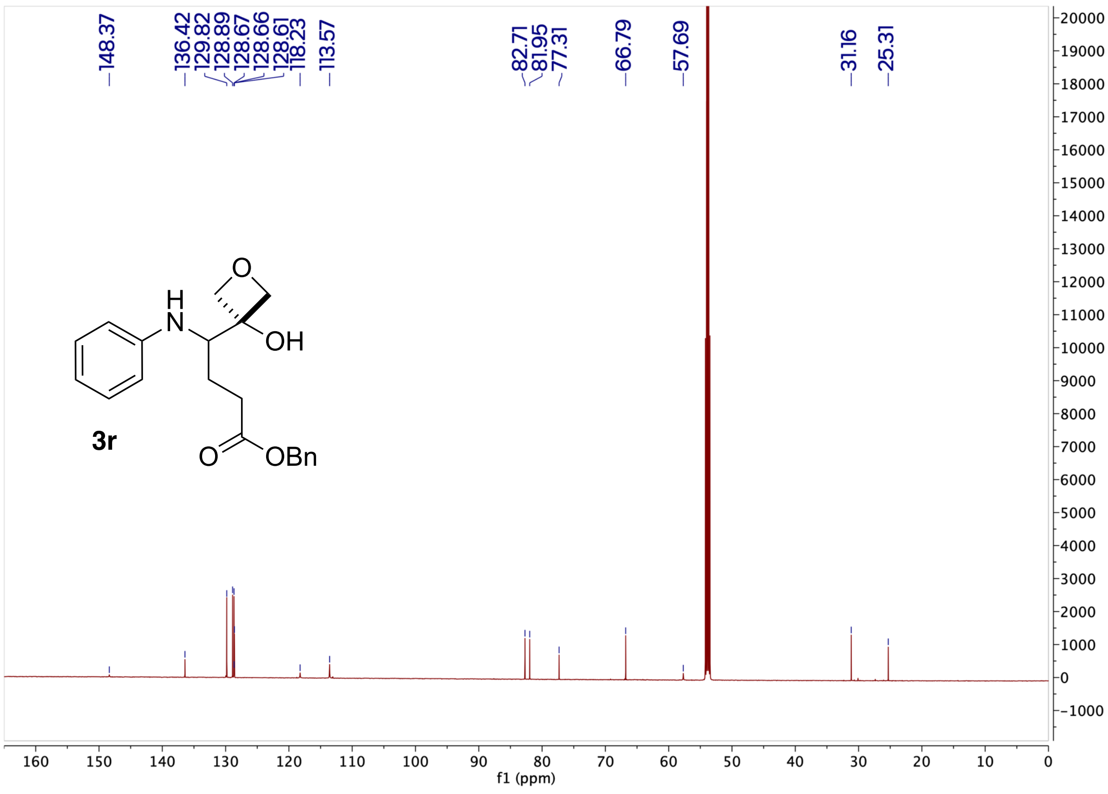
3r
OBn
OH
148.37
136.42
129.82
128.89
128.67
128.66
128.61
118.23
113.57
82.71
81.95
77.31
66.79
57.69
31.16
25.31
f1 (ppm)

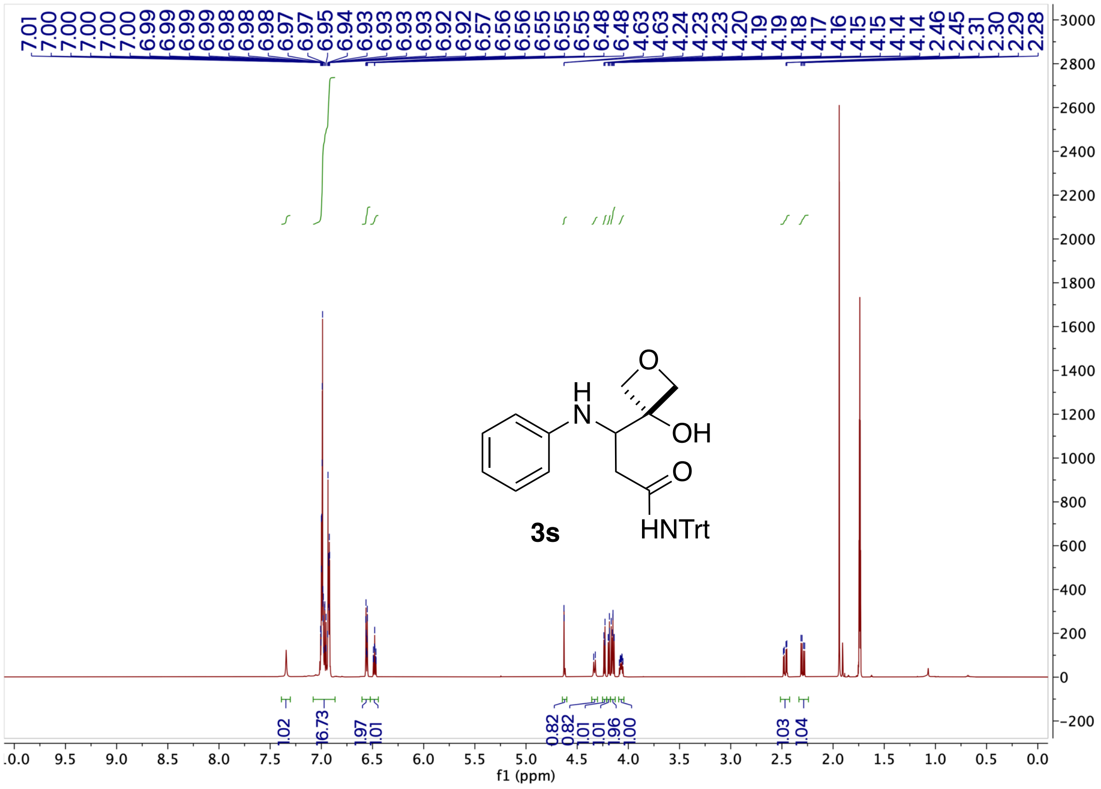
3s
HNTrt
OH
f1 (ppm)

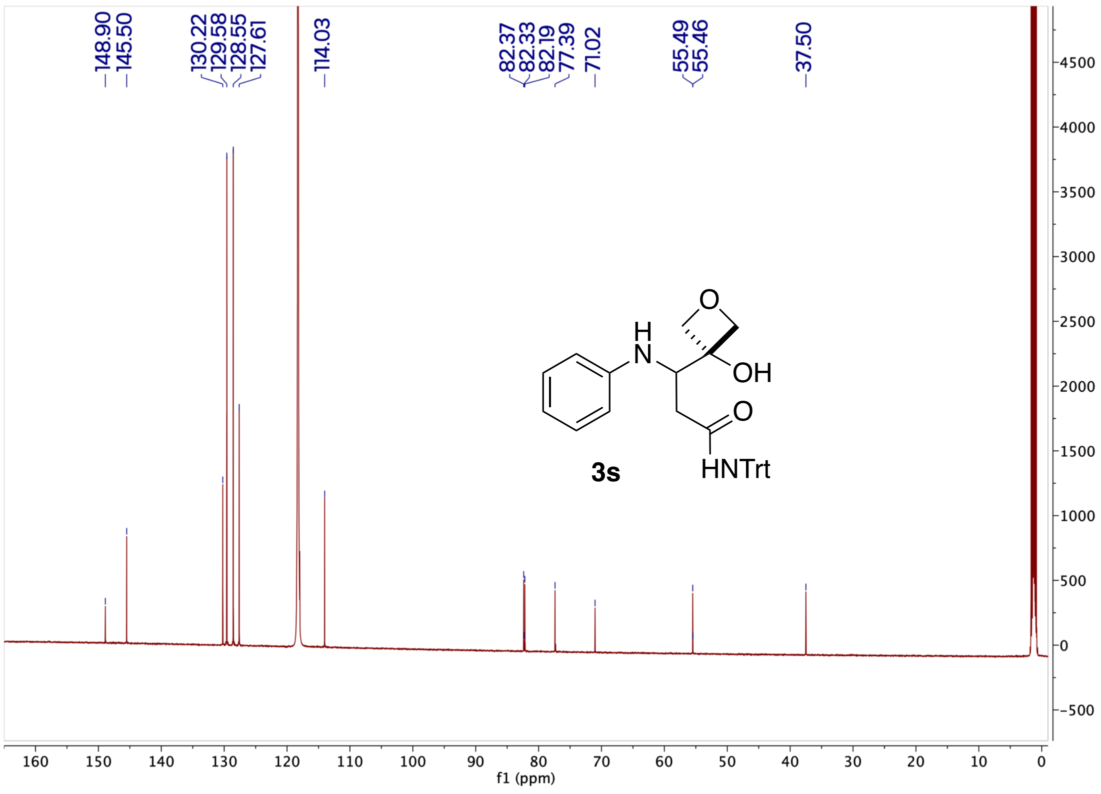
148.90
145.50
130.22
129.58
128.55
127.61
114.03
82.37
82.33
82.19
77.39
71.02
55.49
55.46
37.50
3s
HNTrt
OH
f1 (ppm)

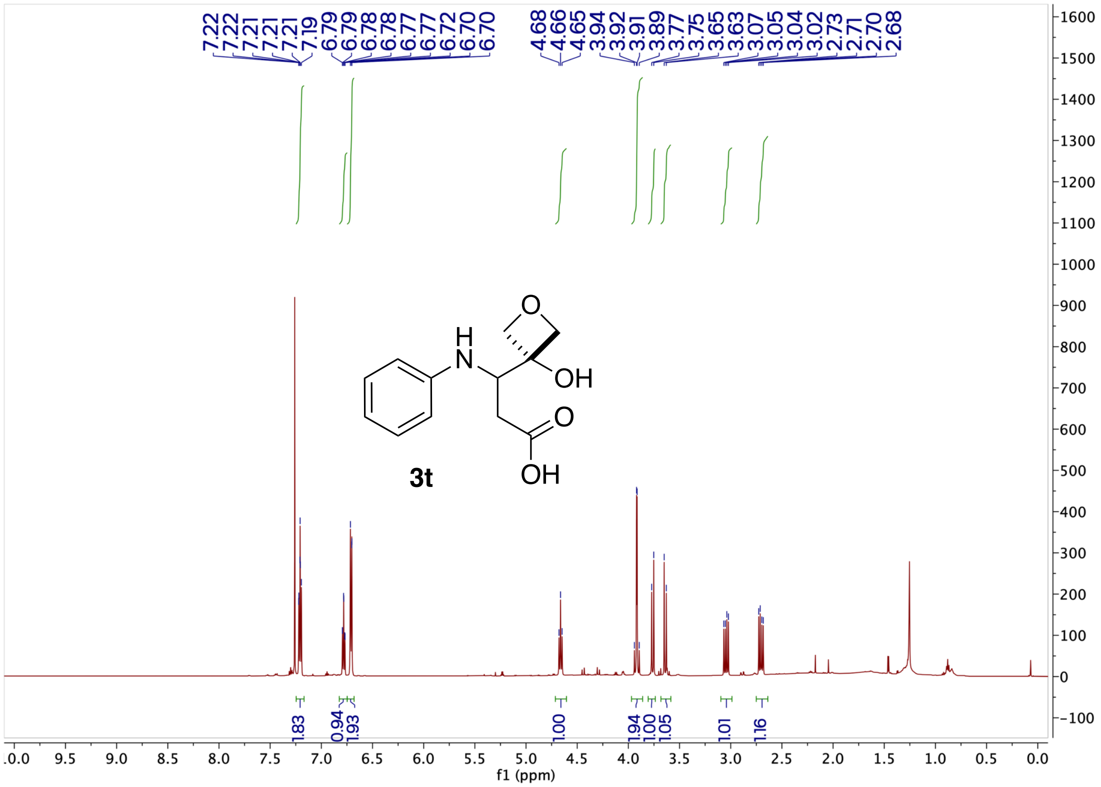
7.22
7.22
7.21
7.21
7.21
7.19
6.79
6.79
6.78
6.78
6.77
6.77
6.72
6.70
6.70
4.68
4.66
4.65
3.94
3.92
3.91
3.89
3.77
3.75
3.65
3.63
3.07
3.05
3.04
3.02
2.73
2.71
2.70
2.68
H
N
O
OH
O
OH
3t
1.83
0.94
1.93
1.00
1.94
1.00
1.05
1.01
1.16
f1 (ppm)

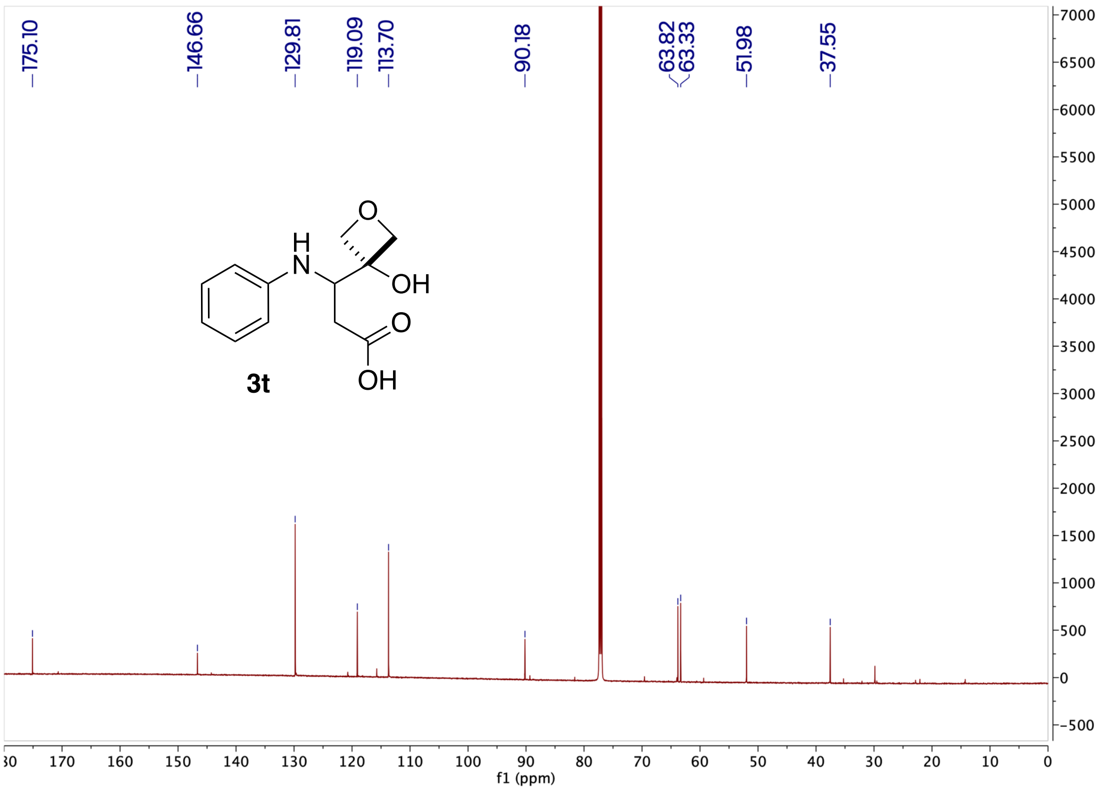
175.10
146.66
129.81
119.09
113.70
90.18
63.82
63.33
51.98
37.55
H
N
O
OH
O
OH
3t
f1 (ppm)

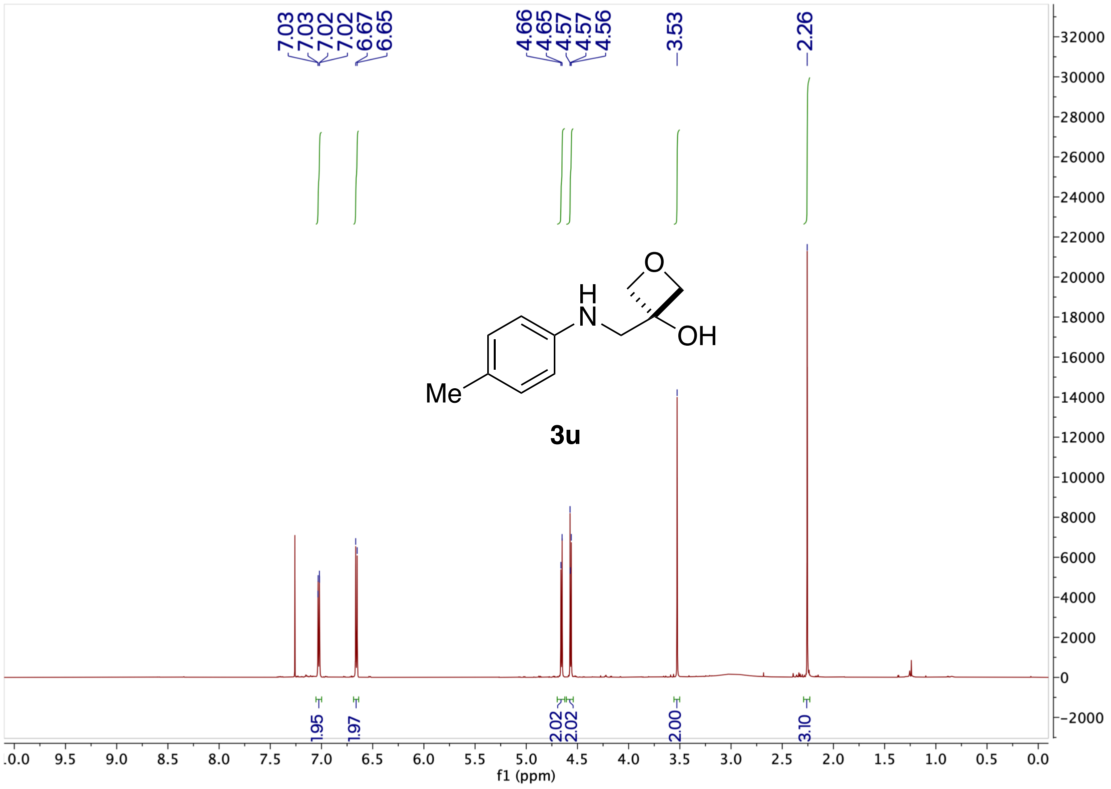
7.03
7.03
7.02
7.02
6.67
6.65
4.66
4.65
4.57
4.57
4.56
3.53
2.26
Me
H
N
O
OH
3u
1.95
1.97
2.02
2.02
2.00
3.10
f1 (ppm)

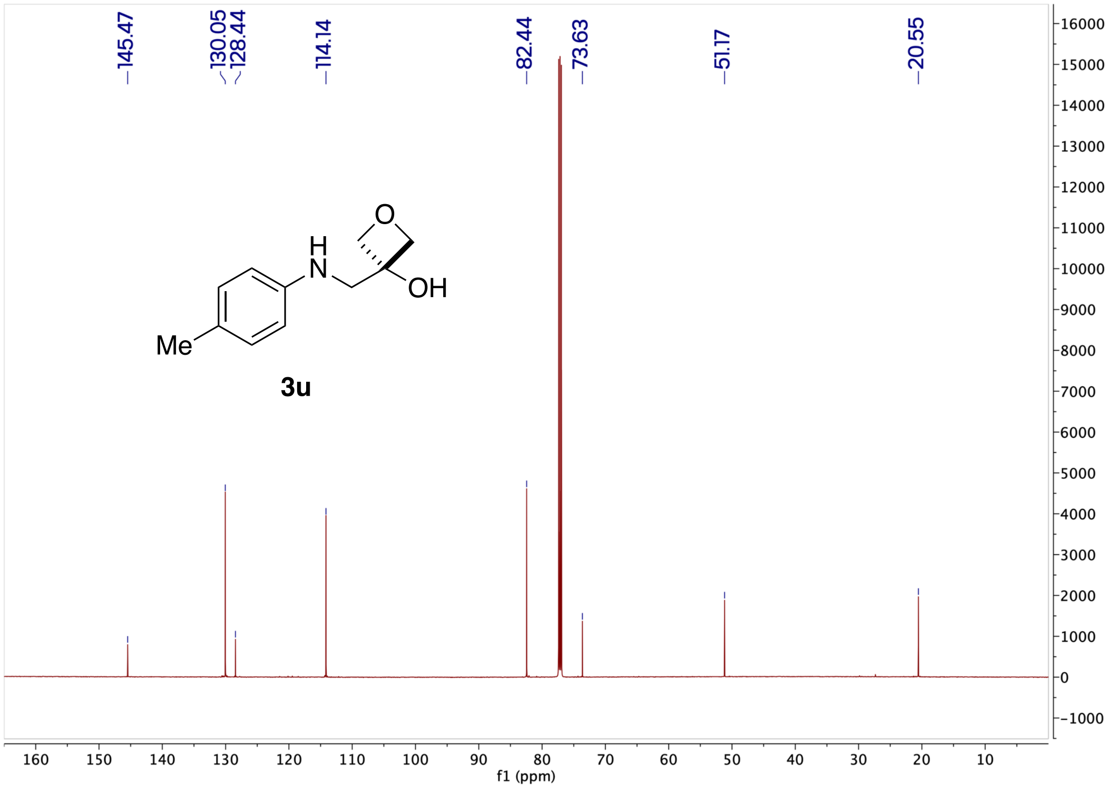
145.47
130.05
128.44
114.14
82.44
73.63
51.17
20.55
Me
H
N
O
OH
3u
f1 (ppm)

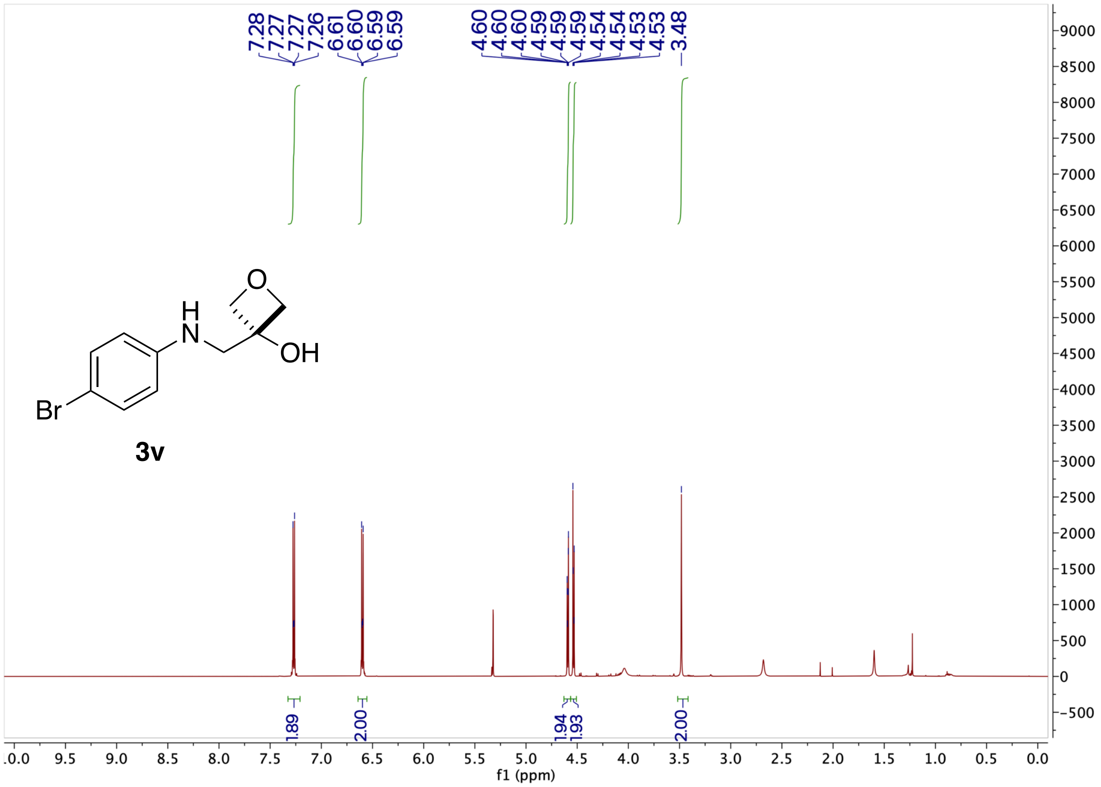
7.28
7.27
7.27
7.26
6.61
6.60
6.59
6.59
4.60
4.60
4.60
4.59
4.59
4.59
4.54
4.54
4.53
4.53
3.48
Br
3v
1.89
2.00
1.94
1.93
2.00
f1 (ppm)

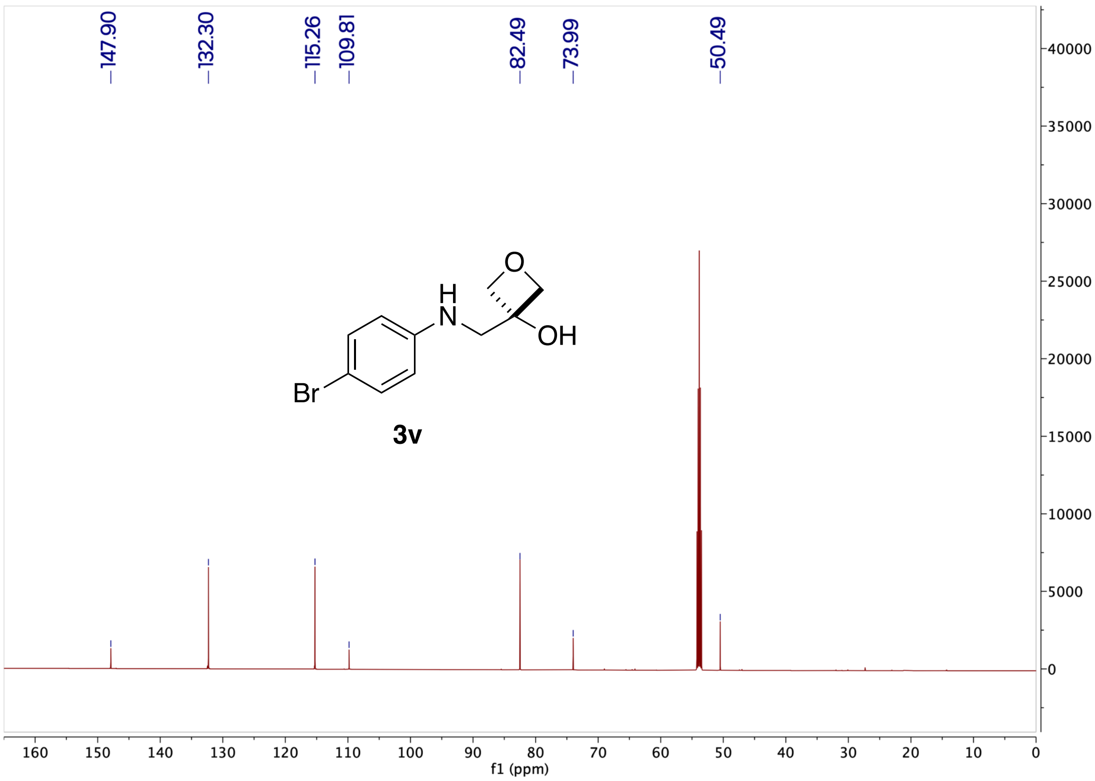
147.90
132.30
115.26
109.81
82.49
73.99
50.49
Br
3v
f1 (ppm)

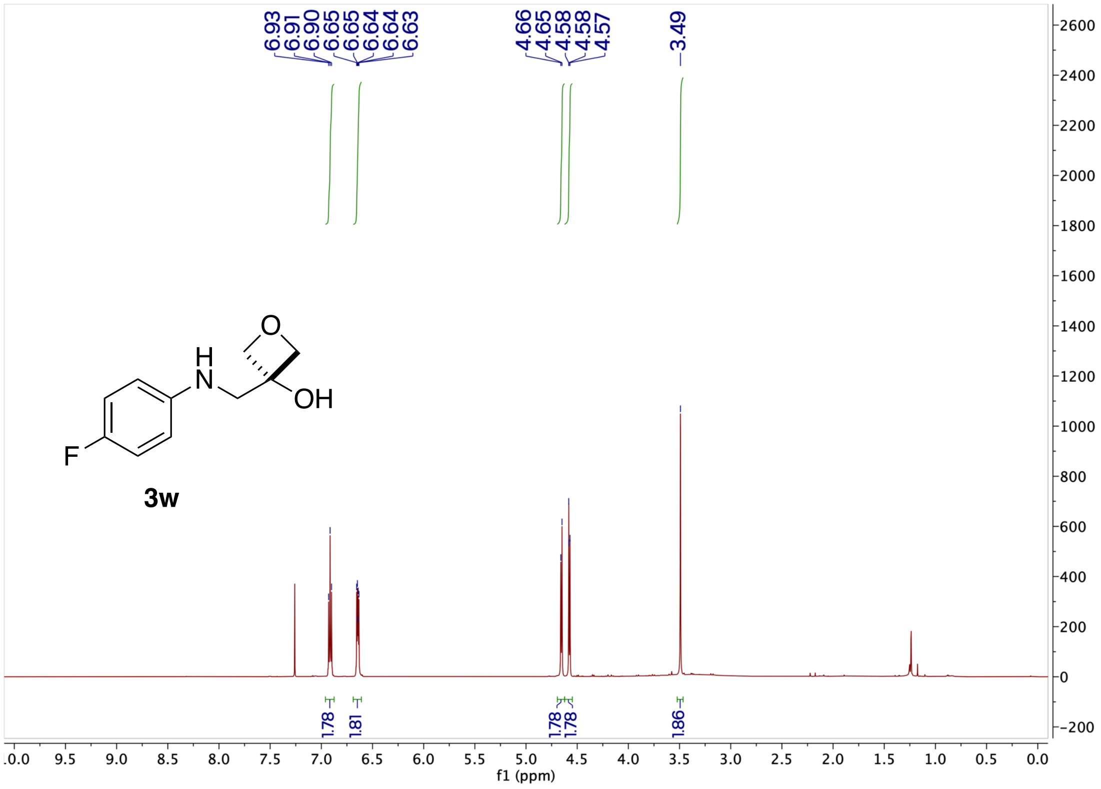
6.93
6.91
6.90
6.65
6.65
6.64
6.64
6.63
4.66
4.65
4.58
4.58
4.57
3.49
H
N
O
OH
F
3w
1.78
1.81
1.78
1.78
1.86
f1 (ppm)

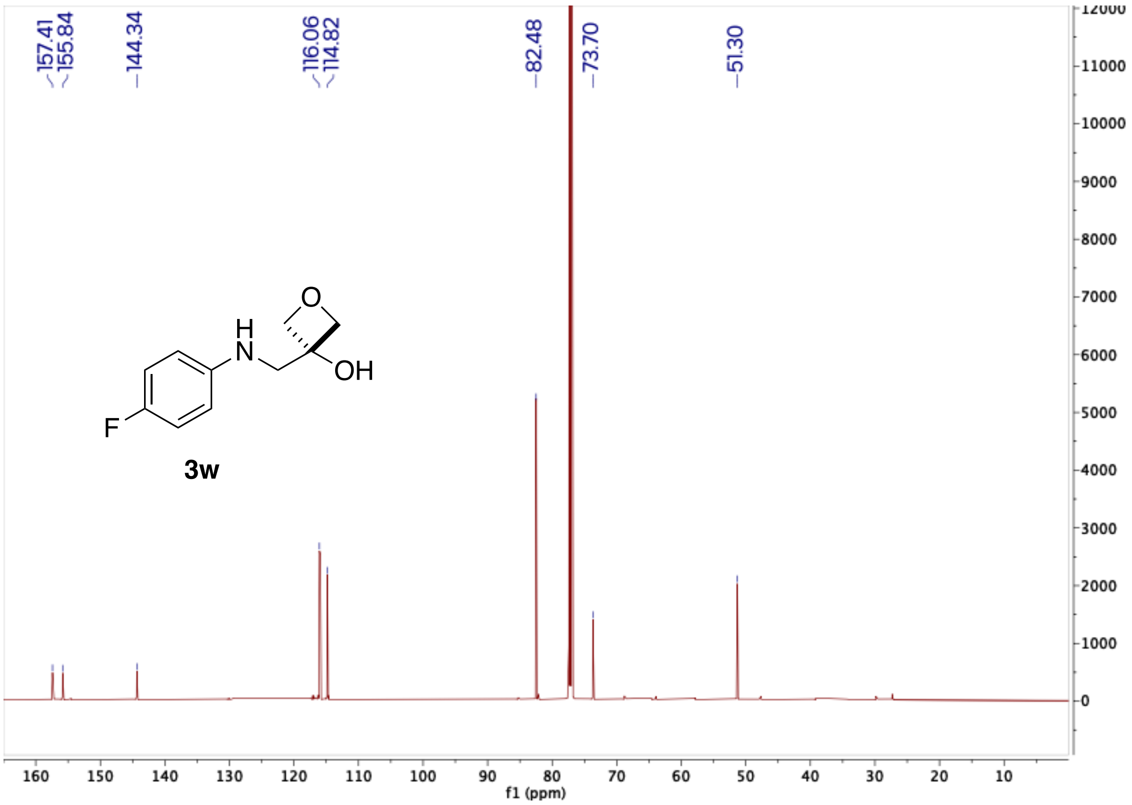
157.41
155.84
144.34
116.06
114.82
82.48
73.70
51.30
H
N
O
OH
F
3w
f1 (ppm)

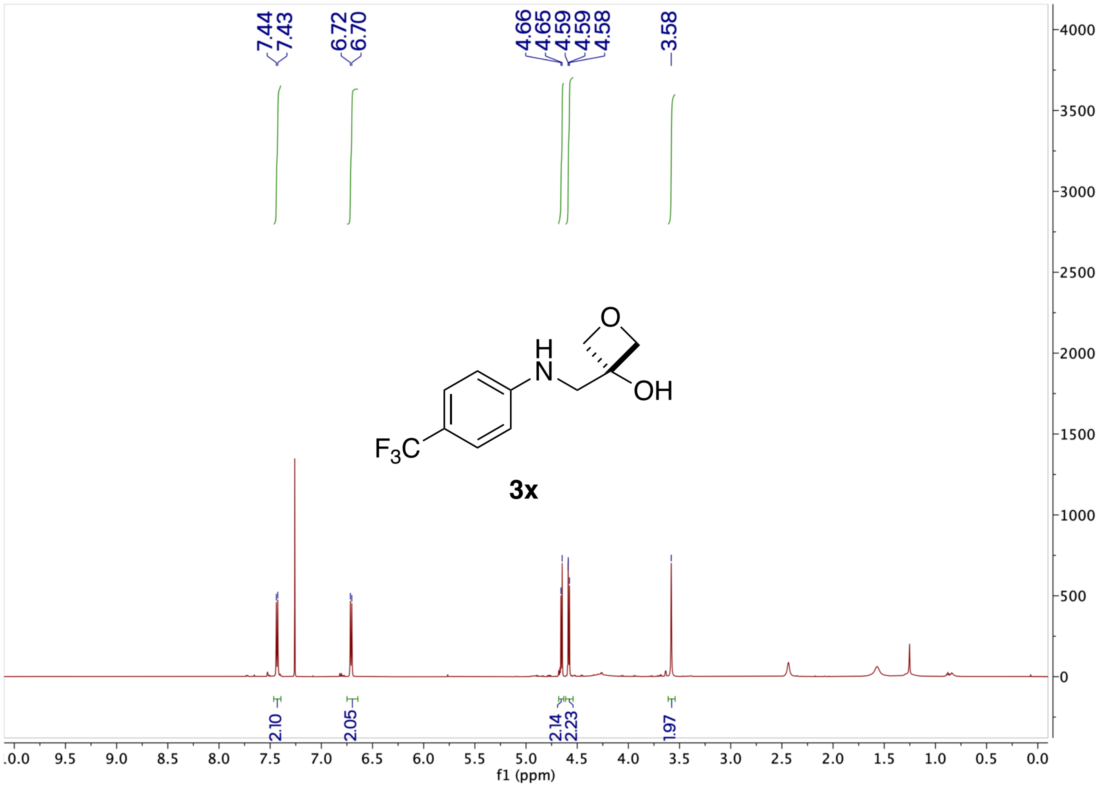
7.44
7.43
6.72
6.70
4.66
4.65
4.59
4.59
4.58
3.58
3x
2.10
2.05
2.14
2.23
1.97
f1 (ppm)

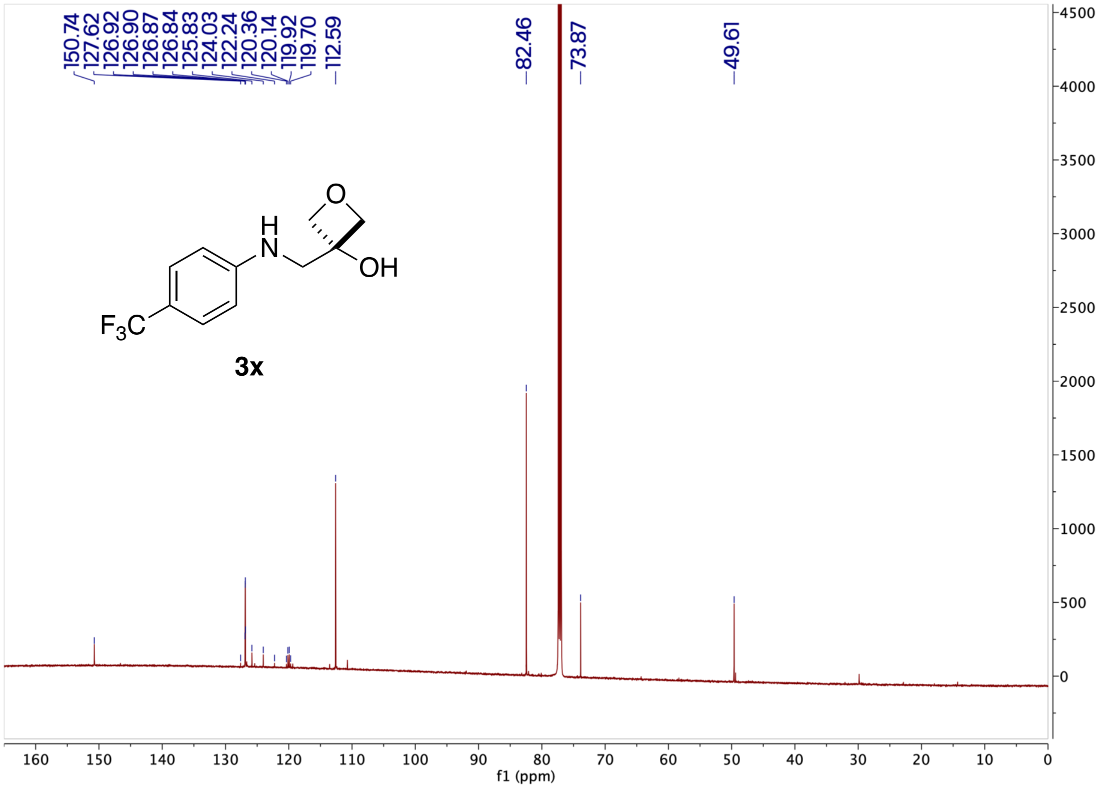
150.74
127.62
126.92
126.90
126.87
126.84
125.83
124.03
122.24
120.36
120.14
119.92
119.70
112.59
82.46
73.87
49.61
3x
f1 (ppm)

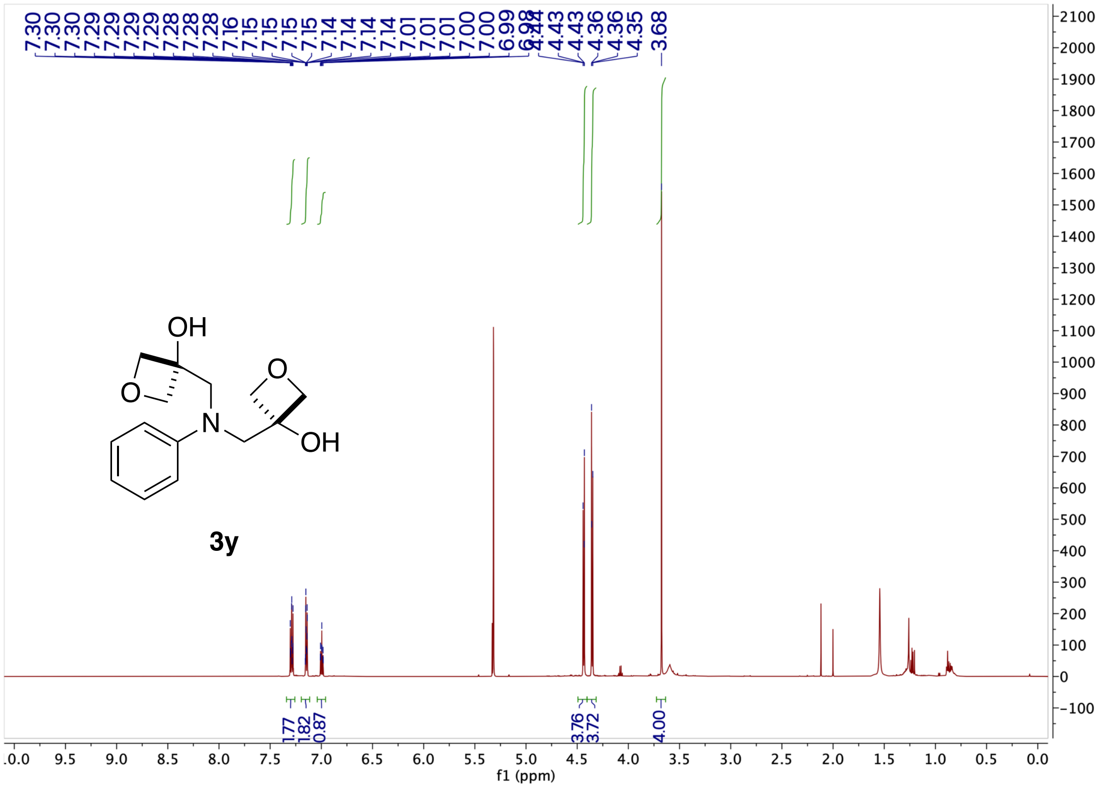

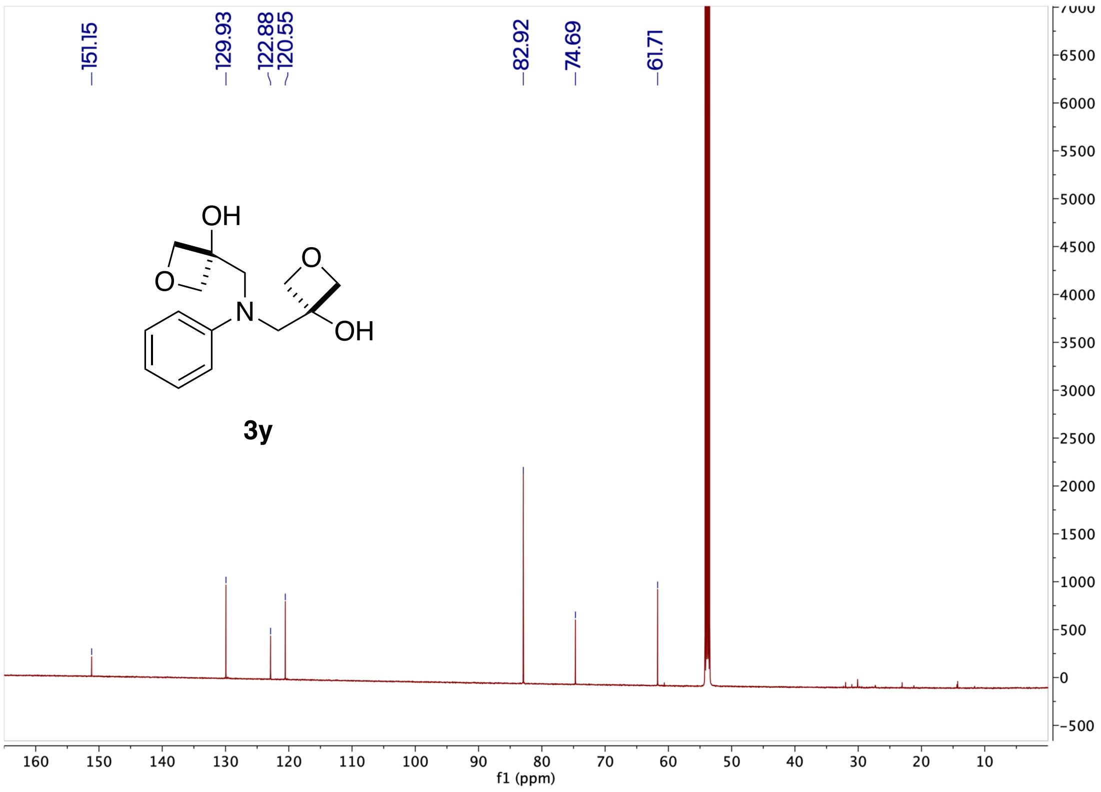

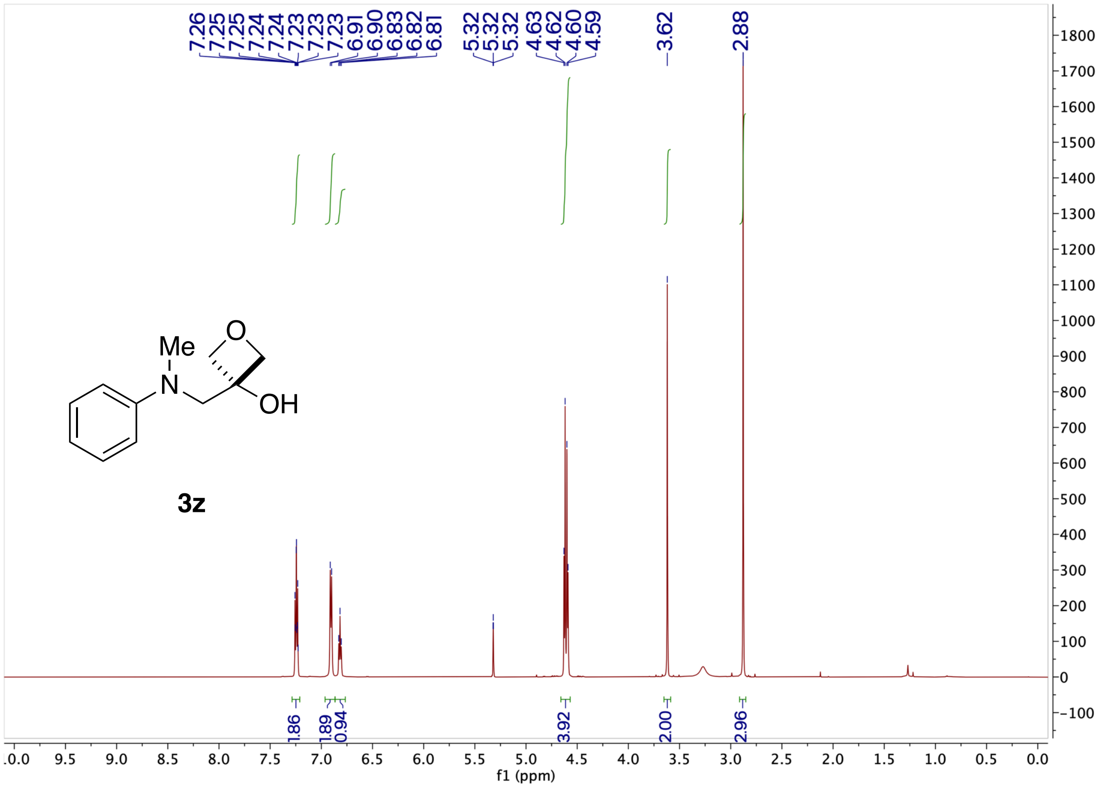
7.26
7.25
7.25
7.24
7.24
7.23
7.23
7.23
6.91
6.90
6.83
6.82
6.81
5.32
5.32
5.32
4.63
4.62
4.60
4.59
3.62
2.88
1.86
1.89
0.94
3.92
2.00
2.96
Me
N
O
OH
3z
f1 (ppm)

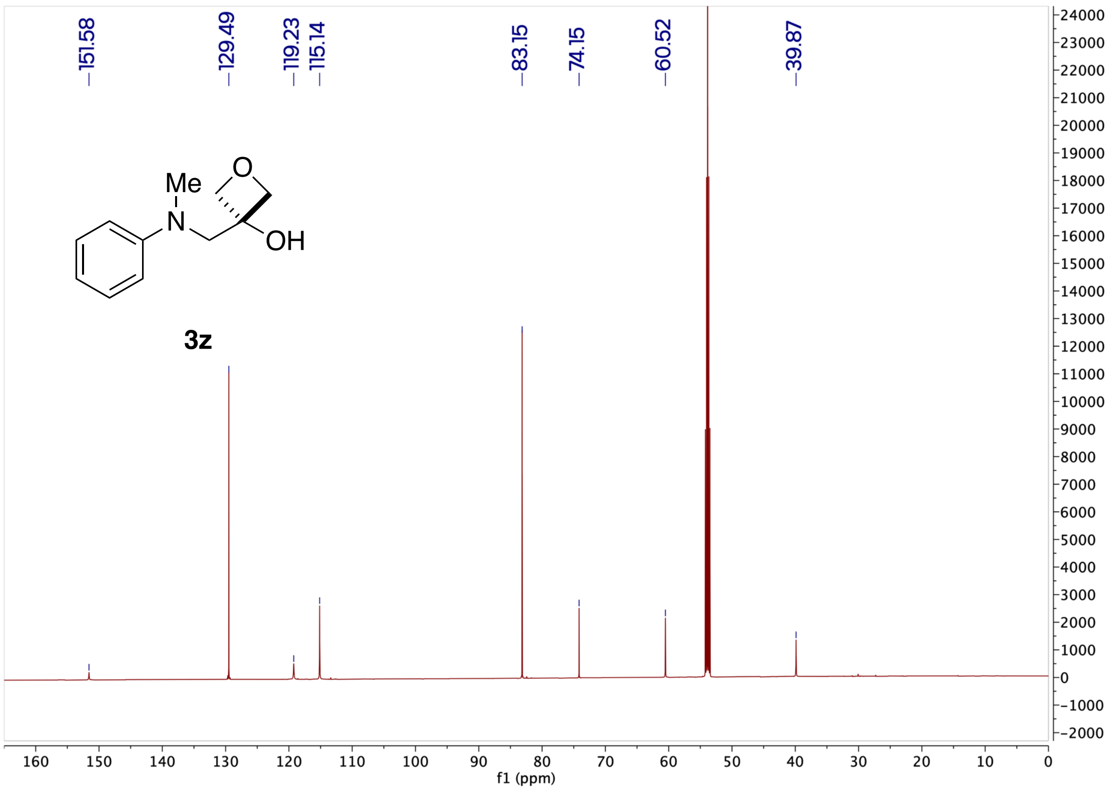
151.58
129.49
119.23
115.14
83.15
74.15
60.52
39.87
Me
N
O
OH
3z
f1 (ppm)

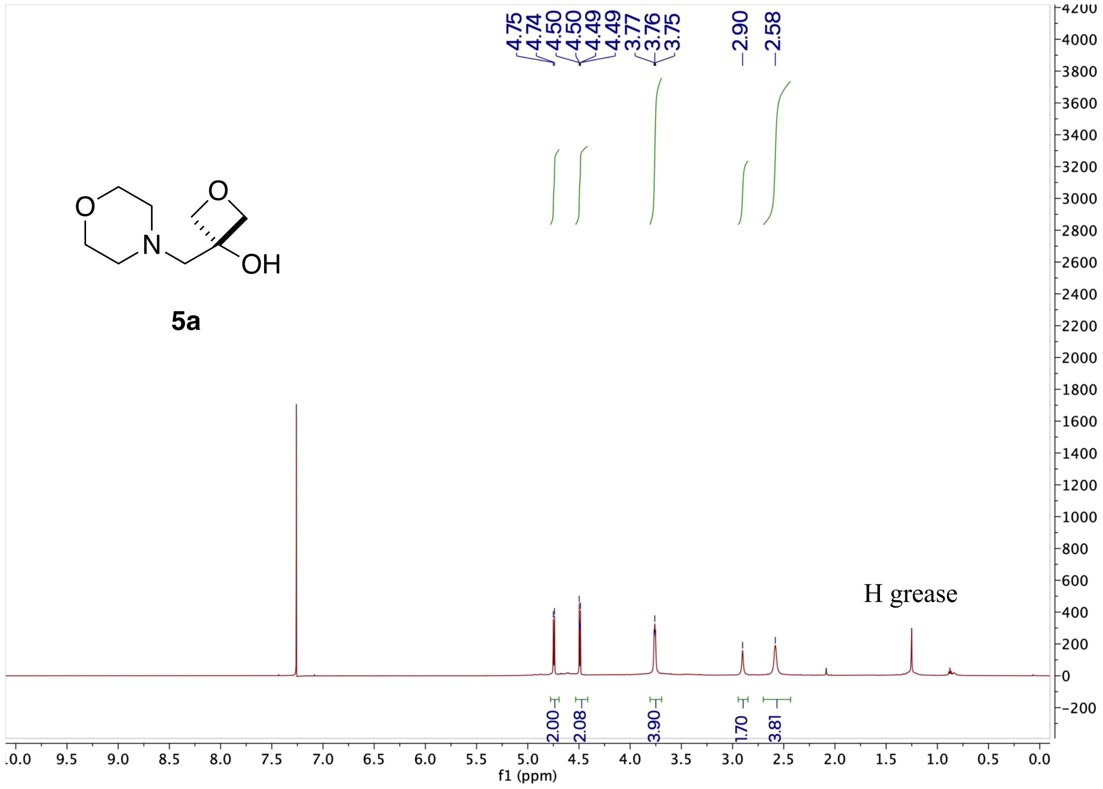

5a
4.75
4.74
4.50
4.50
4.49
4.49
3.77
3.76
3.75
2.90
2.58
H grease
2.00
2.08
3.90
1.70
3.81
f1 (ppm)

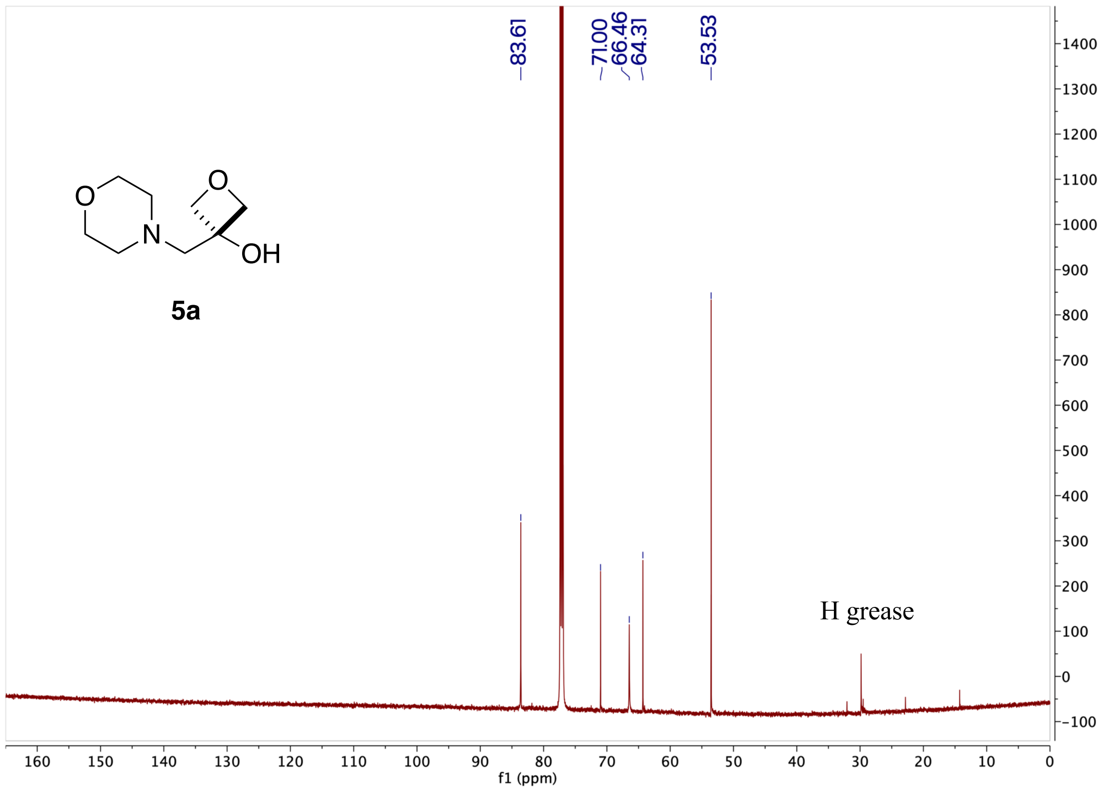

5a
83.61
71.00
66.46
64.31
53.53
H grease
f1 (ppm)

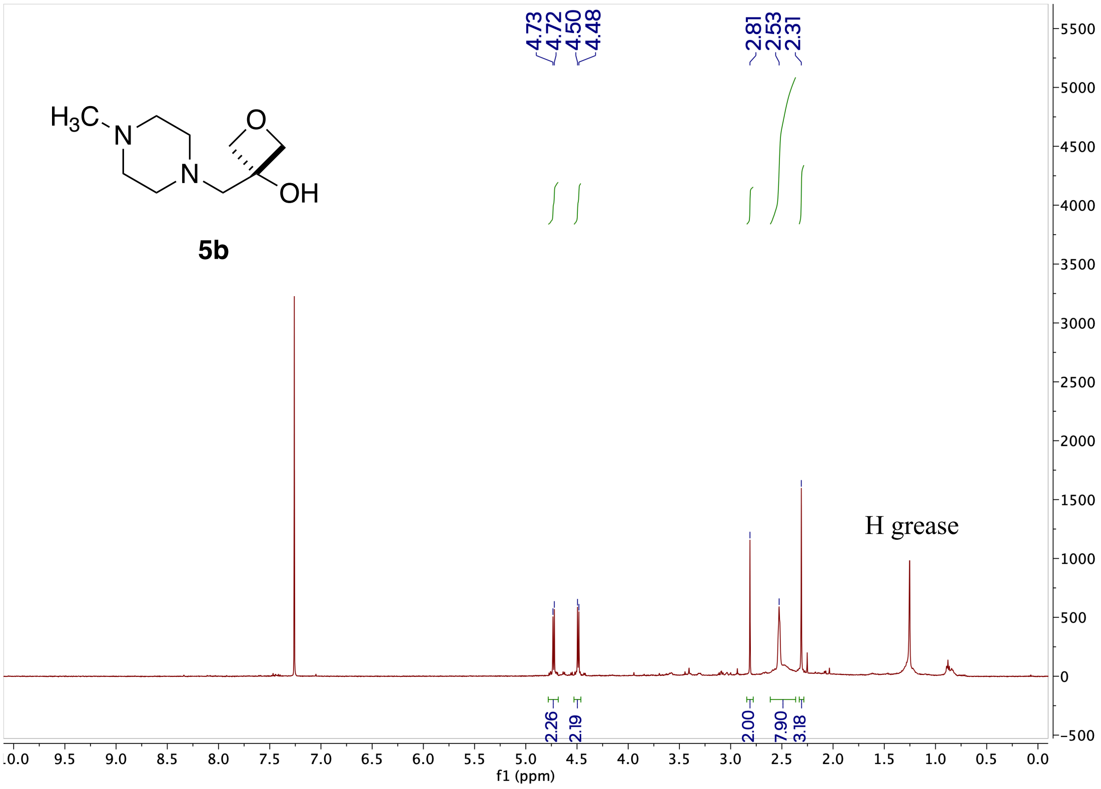
4.73
4.72
4.50
4.48
2.81
2.53
2.31
H grease
2.26
2.19
2.00
7.90
3.18
f1 (ppm)
5b

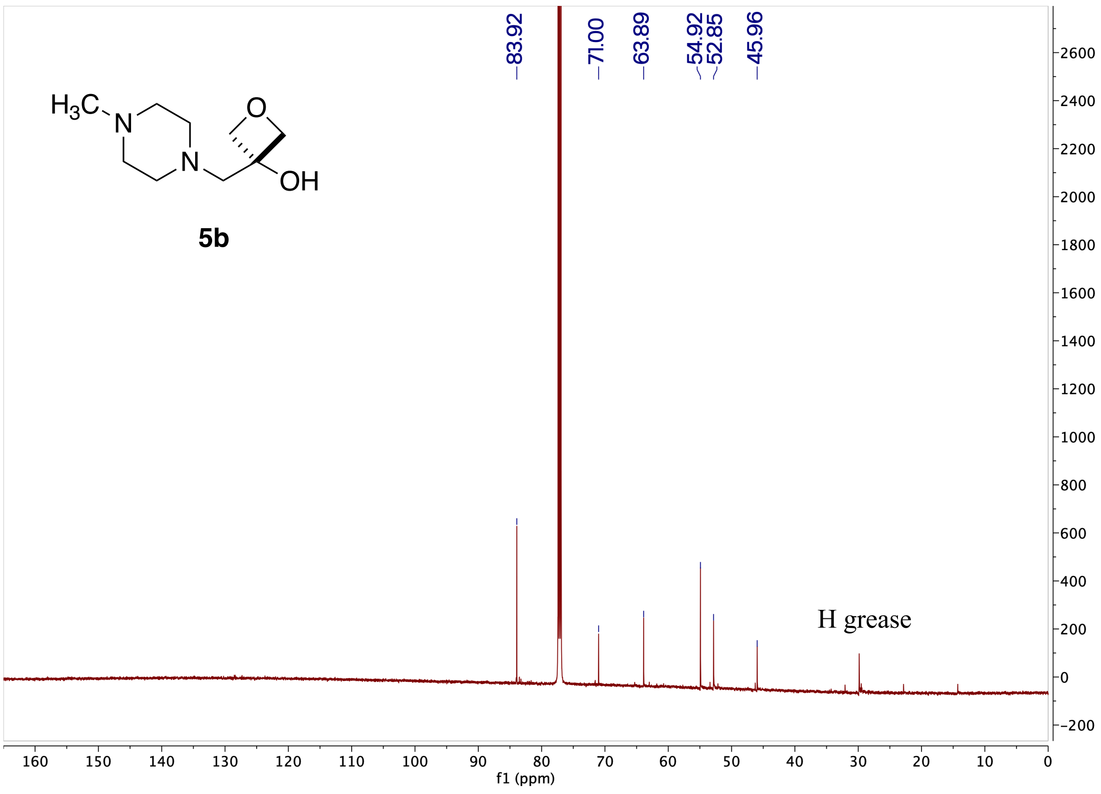
83.92
71.00
63.89
54.92
52.85
45.96
H grease
f1 (ppm)
5b

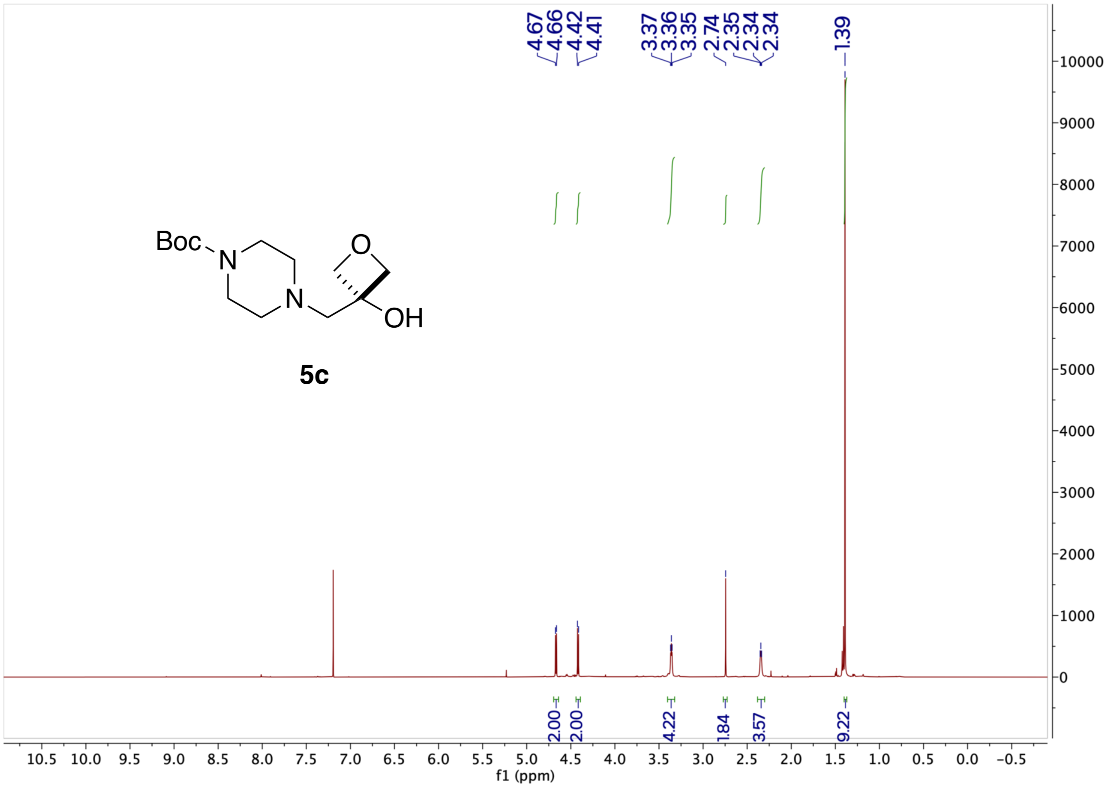

4.67
4.66
4.42
4.41
3.37
3.36
3.35
2.74
2.35
2.34
2.34
1.39
Boc
N
N
O
OH
5c
2.00
2.00
4.22
1.84
3.57
9.22
f1 (ppm)

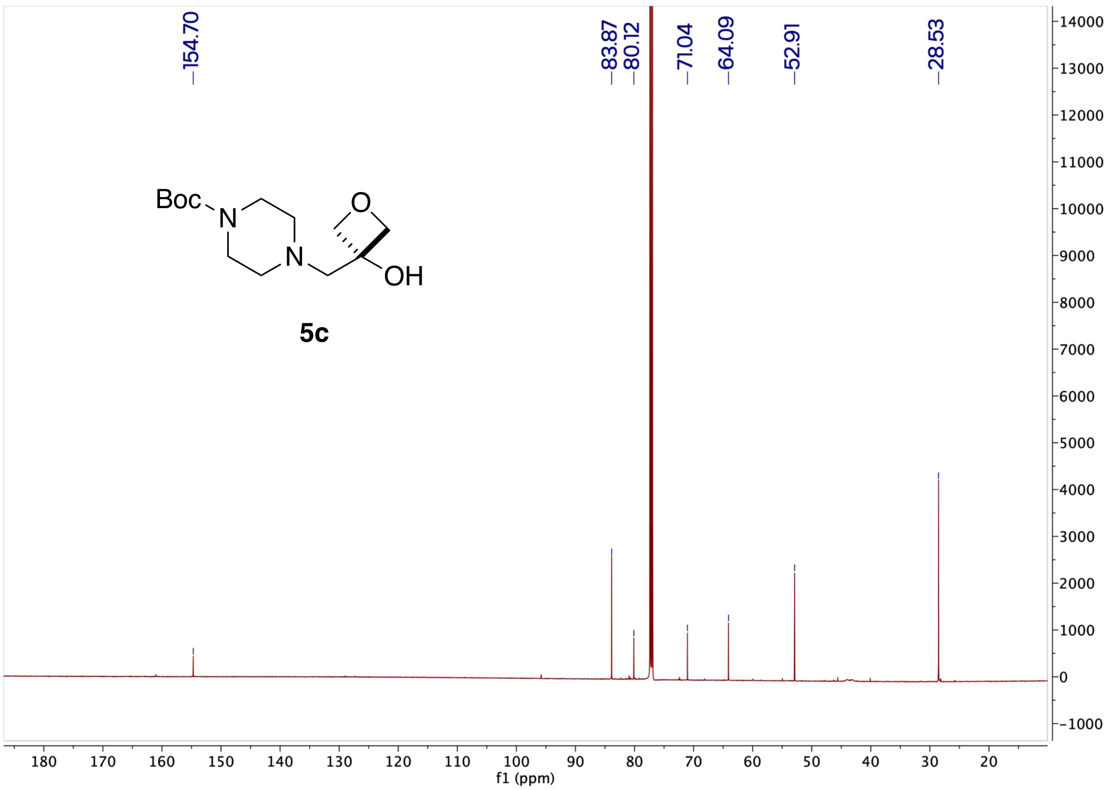

154.70
83.87
80.12
71.04
64.09
52.91
28.53
Boc
N
N
O
OH
5c
f1 (ppm)

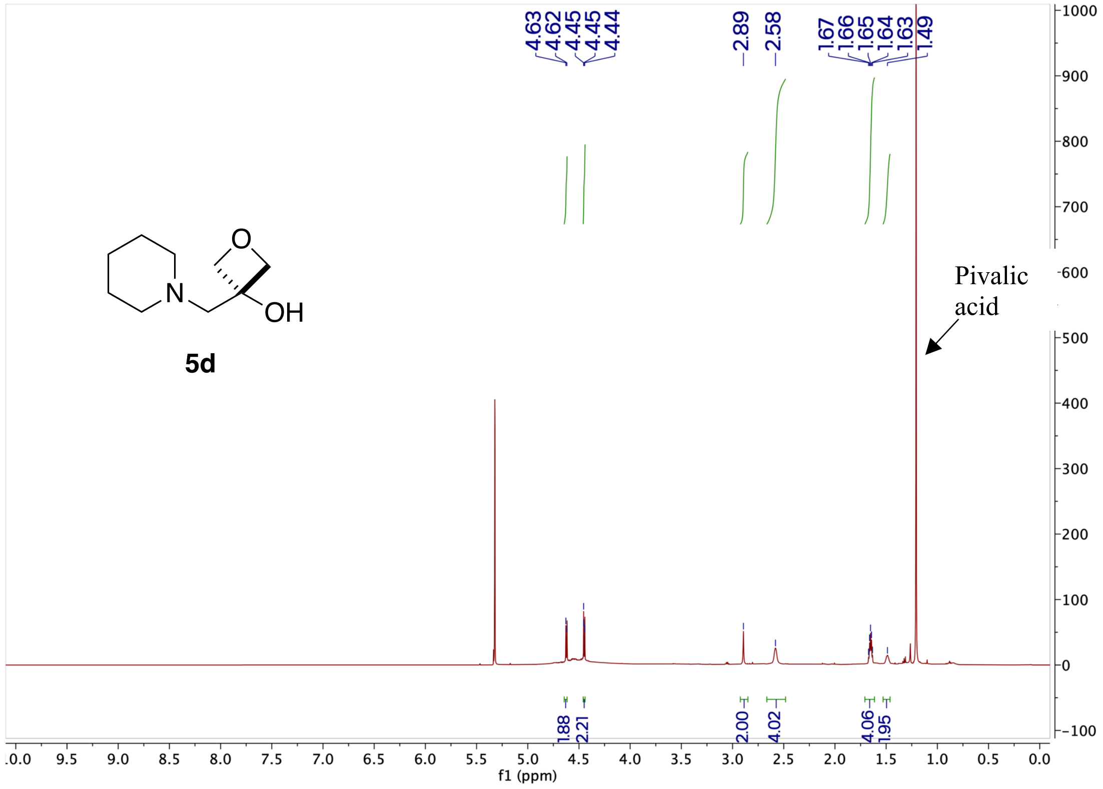
4.63
4.62
4.45
4.45
4.44
2.89
2.58
1.67
1.66
1.65
1.64
1.63
1.49
Pivalic acid
5d
OH
N
O
1.88
2.21
2.00
4.02
4.06
1.95
f1 (ppm)

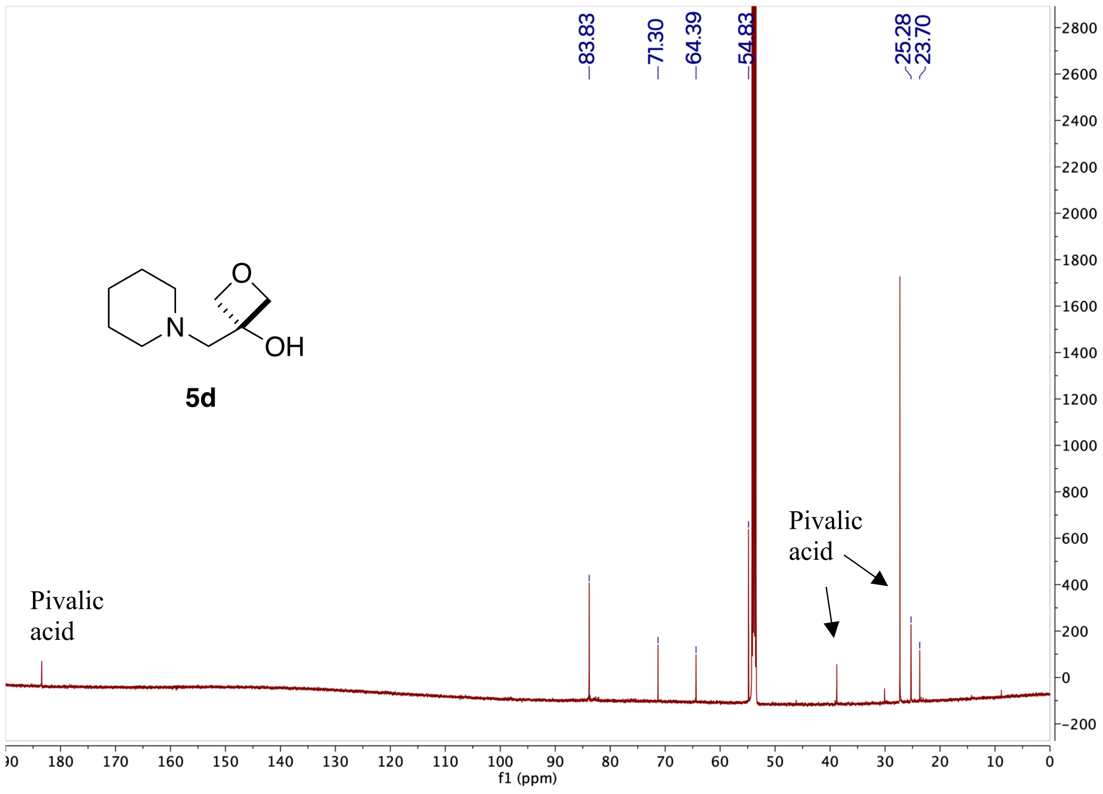
83.83
71.30
64.39
54.83
25.28
23.70
5d
OH
N
O
Pivalic acid
Pivalic acid
f1 (ppm)

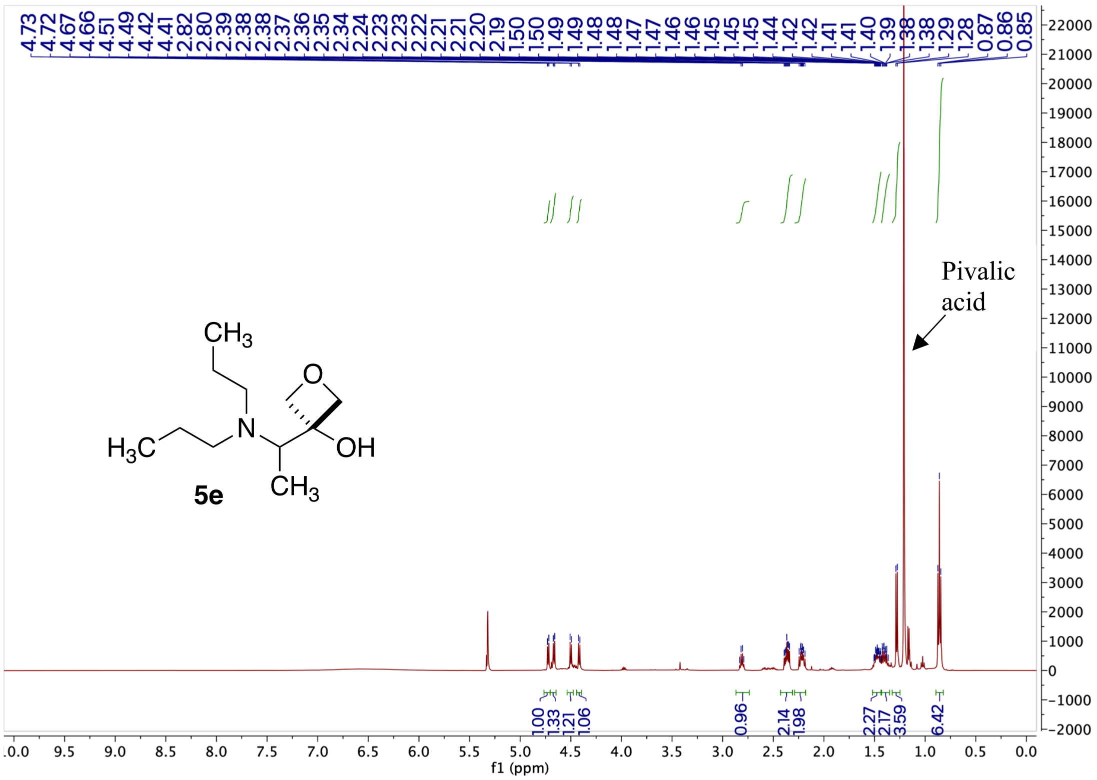
4.73 4.72 4.67 4.66 4.51 4.49 4.42 4.41 2.82 2.80 2.39 2.38 2.38 2.37 2.36 2.35 2.34 2.24 2.23 2.23 2.22 2.21 2.21 2.20 2.19 1.50 1.50 1.49 1.49 1.48 1.48 1.47 1.47 1.46 1.46 1.45 1.45 1.45 1.44 1.42 1.42 1.41 1.41 1.40 1.39 1.38 1.38 1.29 1.28 0.87 0.86 0.85
Pivalic acid
CH_3
O
H_3C
N
OH
5e
CH_3
1.00 1.33 1.21 1.06 0.96 2.14 1.98 2.27 2.17 3.59 6.42
f1 (ppm)

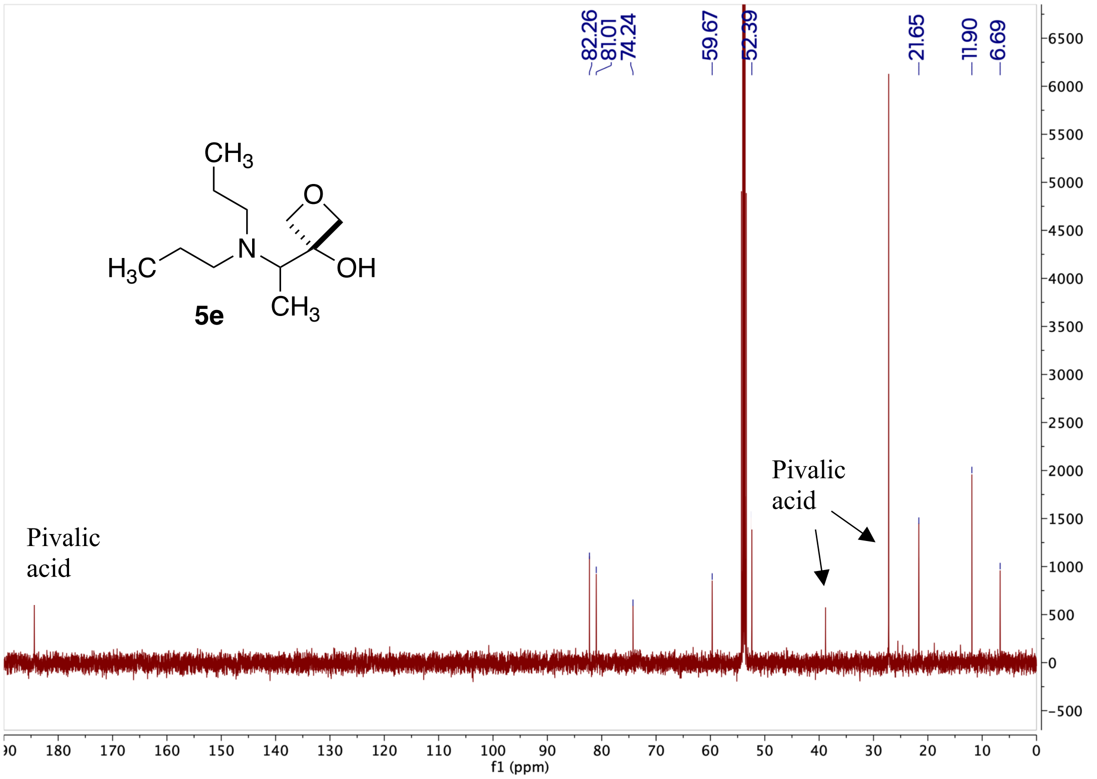
82.26 81.01 74.24 59.67 52.39 21.65 11.90 6.69
CH_3
O
H_3C
N
OH
5e
CH_3
Pivalic acid
Pivalic acid
Pivalic acid
f1 (ppm)
